# Mechanistic investigations on multiproduct β-himachalene synthase from *Cryptosporangium arvum*

**DOI:** 10.3762/bjoc.15.99

**Published:** 2019-05-02

**Authors:** Jan Rinkel, Jeroen S Dickschat

**Affiliations:** 1 Kekulé-Institute for Organic Chemistry and Biochemistry, University of Bonn, Gerhard-Domagk-Str. 1, 53121 Bonn, Germany

**Keywords:** enzyme mechanisms, isotopes, mass spectrometry, promiscuity, terpenes

## Abstract

A bacterial terpene synthase from *Cryptosporangium arvum* was characterised as a multiproduct β-himachalene synthase. In vitro studies showed not only a high promiscuity with respect to its numerous sesquiterpene products, including the structurally demanding terpenes longicyclene, longifolene and α-longipinene, but also to its substrates, as additional activity was observed with geranyl- and geranylgeranyl diphosphate. In-depth mechanistic investigations using isotopically labelled precursors regarding the stereochemical course of both 1,11-cyclisation and 1,3-hydride shift furnished a detailed catalytic model suggesting the molecular basis of the observed low product selectivity. The enzyme’s synthetic potential was also exploited in the preparation of sesquiterpene isotopomers, which provided insights into their EIMS fragmentation mechanisms.

## Introduction

The organic chemist usually prefers to work with pure compounds which lead to high requirements for the selectivity of reactions and often to tedious purification procedures, but encountering a pure compound in nature is quite rare. This does not result in reduced requirements for enzyme selectivity. The very opposite is mostly true, because proteins working in a compound mixture need to be precise [1]. However, in some cases, compound mixtures have proven to be superior to the properties of the single compounds by evolution. Examples demonstrating this principle can be found in pheromone chemistry, like the bark beetle aggregation blend of ipsdienol, ipsenol and verbenol, for which synergistic effects were observed compared to the single compounds [2]. Also the sex pheromone of the cranberry white grub *Phyllophaga anxia* was identified as a compound mixture, consisting of L-valine methyl ester and L-isoleucine methyl ester at a 3:1 ratio [3]. Moreover, if there is a single enzyme that can produce a beneficial mixture, the advantage for the producing organism is even higher. Therefore, selectivity is not in every case the highest goal for evolution. An enzyme class, which is highly prone to a regulation of product selectivity for the production of either one or multiple compounds, are terpene synthases (TSs). These enzymes are able to guide complex cascade reactions from structurally simple oligoprenyl diphosphates to often complex, polycyclic products [4–6] circumventing the low selectivity observed for carbocationic reactions by a defined active-site architecture. Although these enzymes are mostly highlighted for their great product selectivity, TSs producing only one compound are by far not the general case. Mostly, the main product is accompanied by several side products. Prominent examples are the TS identified from the plant *Medicago truncatula* with at least 27 products [7], γ-humulene synthase from *Abies grandis* with 52 products [8], and also the long known trichodiene synthase from *Fusarium sporotrichioides* produces at least 15 sesquiterpenes [9]. Some TSs can even accept multiple chain length substrates [10], a concept which seems to occur frequently in plants [11]. Whether the reduced selectivity of TSs both for substrates and for products can be attributed to imperfect catalysis, or if this function is even beneficial for the producing organism, remains elusive in most cases. Also the structural basis of promiscuous catalysis by TSs is largely unknown [12]. In this study, we present the characterisation of a bacterial TS with a reduced selectivity both for substrates and for products together with the challenging investigation of its cyclisation mechanism by labelling experiments.

## Results and Discussion

### A bacterial β-himachalene synthase produces numerous side products

**Figure 1:**
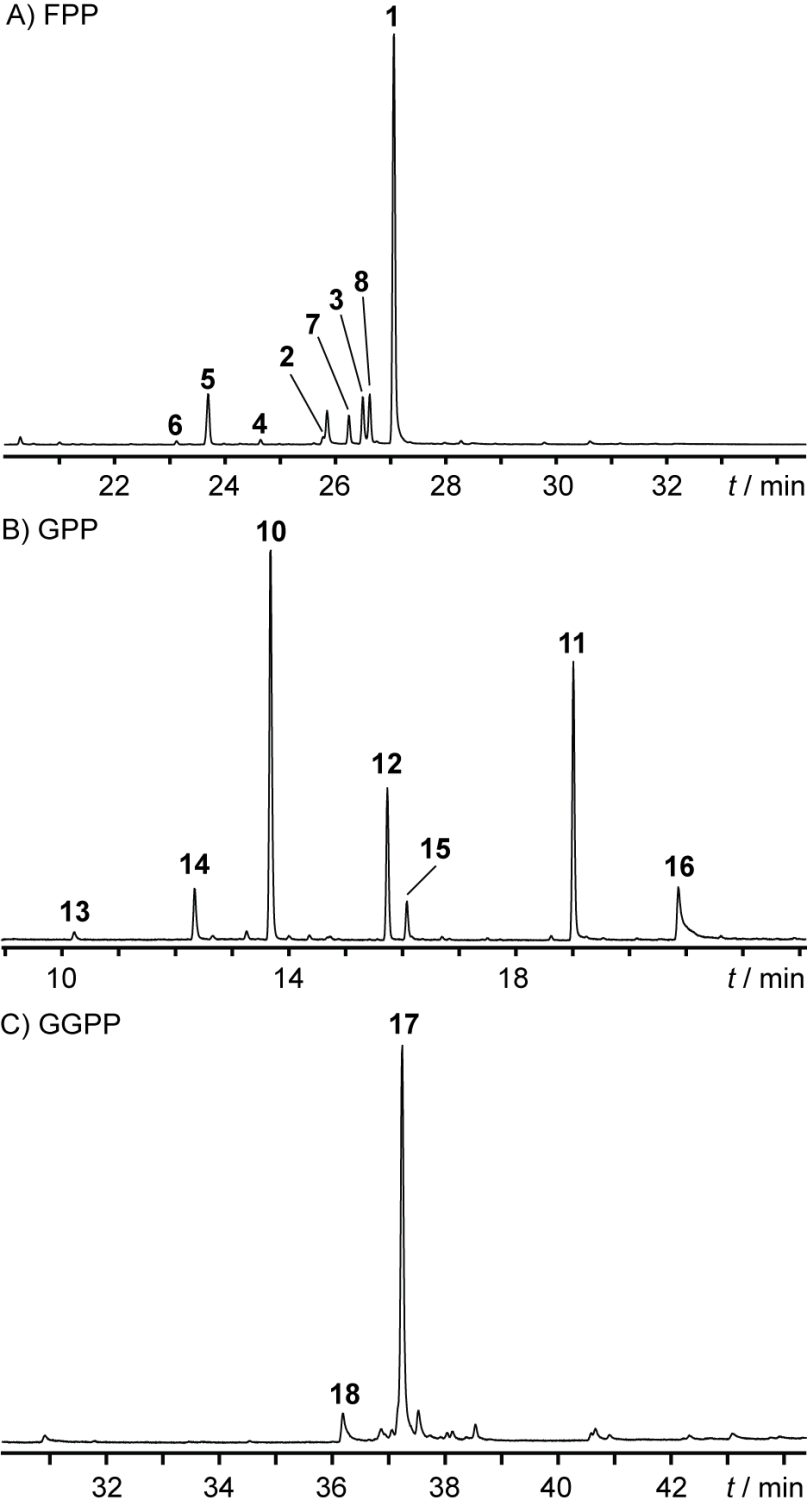
Total ion chromatograms of hexane extracts from the incubations of HcS with A) FPP, B) GPP and C) GGPP. Peak numbering refers to the compounds shown in Figure 2 and listed in Table 1.

**Figure 2:**
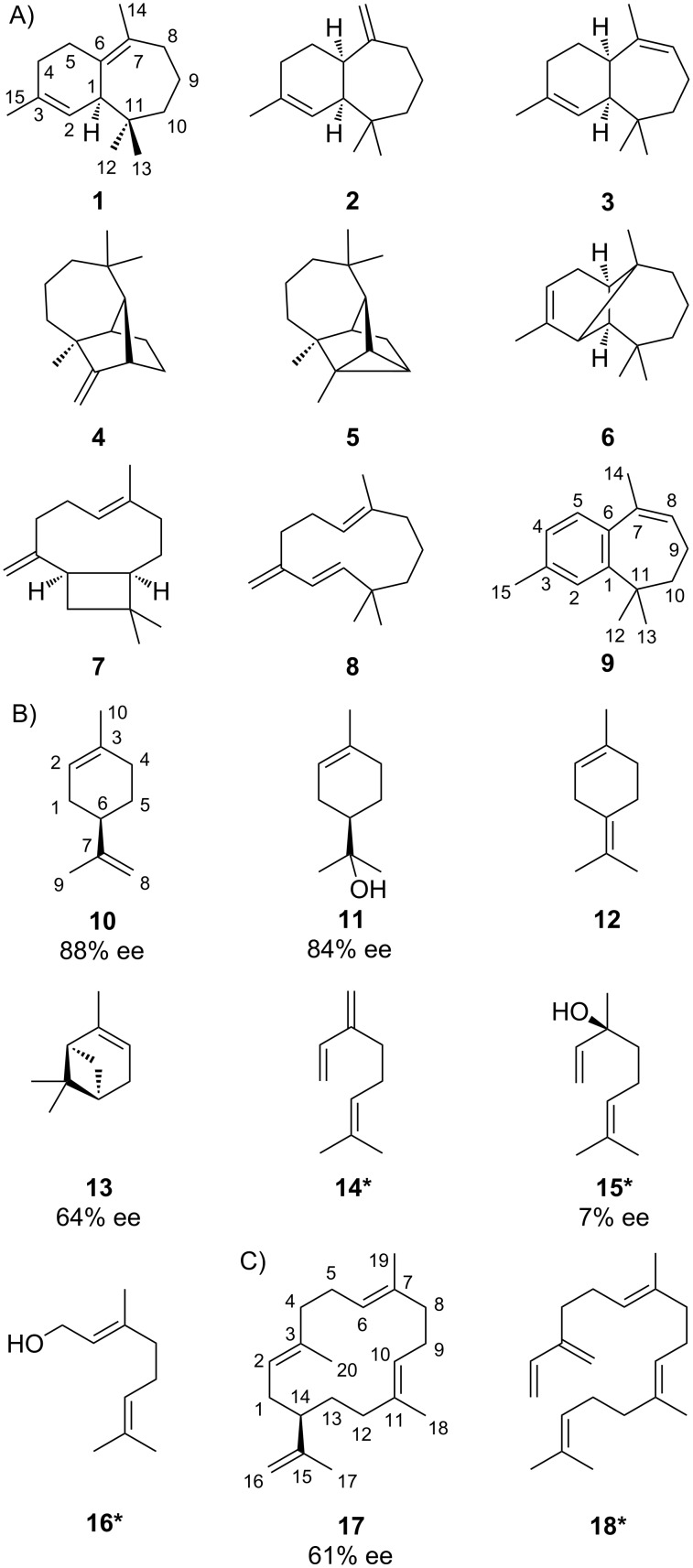
Structures of HcS products arising A) from FPP together with related oxidation product **9**, B) from GPP and C) from GGPP. The carbon numberings of **1** and **9** refer to the carbon positions of FPP as shown in Scheme 3, numberings of **10** and **17** are derived from that of GPP and GGPP, respectively. Compounds known to also originate from non-enzymatic hydrolysis are labelled with an asterisk. The enantiomeric excess values were determined based on GC analysis on a chiral phase.

Apart from the recently assigned (*Z*)-γ-bisabolene synthase (BbS) [13], the soil-dwelling actinomycete *Cryptosporangium arvum* DSM 44712 also possesses a second TS gene (accession no. WP_035852539). Its encoded amino acid sequence (Figure S1, Supporting Information File 1) shares conserved motifs for TSs, but is phylogenetically distant to BbS and does not possess a close characterised relative among other bacterial TSs (Figure S2, Supporting Information File 1). Therefore, its gene was cloned into the *E. coli* expression vector pYE-Express [14] for functional characterisation (Table S1, Supporting Information File 1). The purified recombinant protein (Figure S3, Supporting Information File 1) was incubated with the common TS substrates geranyl- (GPP, C_10_), farnesyl- (FPP, C_15_), geranylgeranyl- (GGPP, C_20_) and geranylfarnesyl (GFPP, C_25_) diphosphate. Whereas the latter diphosphate did not lead to any terpene product, the incubation with FPP showed a smooth conversion into several sesquiterpenes (Figure 1A) with compound **1** as the major peak after GC–MS analysis. However, also the incubations with GPP (Figure 1B) and GGPP (Figure 1C) led to several less complex terpene products, demonstrating a broadened substrate range for this enzyme. The annotated peaks were correlated by mass spectral libraries and retention indices (Table 1) to the known natural products **1**–**8** and **10**–**18** (Figure 2).

**Table 1:** HcS product identification by retention indices.

compound	*I*^a^	*I* (lit.)

from GPP		

α-pinene (**13**)	934	934 [15]
β-myrcene (**14**)	992	992 [16]
limonene (**10**)	1029	1031 [17]
α-terpinolene (**12**)	1089	1088 [18]
linalool (**15**)	1099	1098 [19]
α-terpineol (**11**)	1191	1190 [19]
geraniol (**16**)	1250	1253 [20]

from FPP		

α-longipinene (**6**)	1355	1356 [21]
longicyclene (**5**)	1376	1377 [21]
longifolene (**4**)	1412	1413 [21]
α-himachalene (**2**)	1461	1461 [22]
9-*epi*-β-caryophyllene (**7**)	1475	1471 [23]
γ-humulene (**8**)	1489	1487 [24]
γ-himachalene (**3**)	1490	1489 [22]
β-himachalene (**1**)	1507	1503 [25]

from GGPP		

β-springene (**18**)	1921	1918 [26]
cembrene A (**17**)	1974	1979 [27]

^a^Retention index *I* on a HP-5MS column.

In a large scale incubation, β-himachalene (**1**) was isolated, accompanied by smaller amounts of the double oxidation product γ-dehydro-*ar*-himachalene (**9**). Since **9** was only observed after prolonged incubation times, an auto-oxidation mechanism involving oxygen is assumed. Both compounds were analysed by one- and two-dimensional NMR spectroscopy (Tables S2 and S3, Supporting Information File 1). The absolute configuration of **1** was determined as the (+)-enantiomer, unanimously by optical rotary power measurement and an isotopic labelling strategy, which involved conversion of stereoselectively deuterated and at the same position ^13^C-labelled FPPs by the TS to yield labelled **1** with incorporation of deuterium into diastereotopic hydrogen positions. Together with the relative configuration of the targeted methylene group as deduced by NOESY, the stereochemical outcome of these experiments, which can be easily monitored by sensitive HSQC, infers the absolute configuration of **1**. For **1**, C-5 was targeted by (1*R*)- and (1*S*)-(1-^13^C,1-^2^H)GPP [28], which were enzymatically elongated with isopentenyl diphosphate (IPP) by farnesyl diphosphate synthase (FPPS) from *Streptomyces coelicolor* [29] with a known stereochemical course [30] (Figure S4, Supporting Information File 1). This principle was also applied to C-4 and C-8 utilising (*Z*)- and (*E*)-(4-^13^C,4-^2^H)IPP [31] for the elongation of dimethylallyl diphosphate (DMAPP) catalysed by FPPS in a known course [32]. Since both hydrogens at C-4 possess the same chemical shift, only C-8 could be used to solidify the absolute configuration (Figure S5, Supporting Information File 1).

**Scheme 1:**
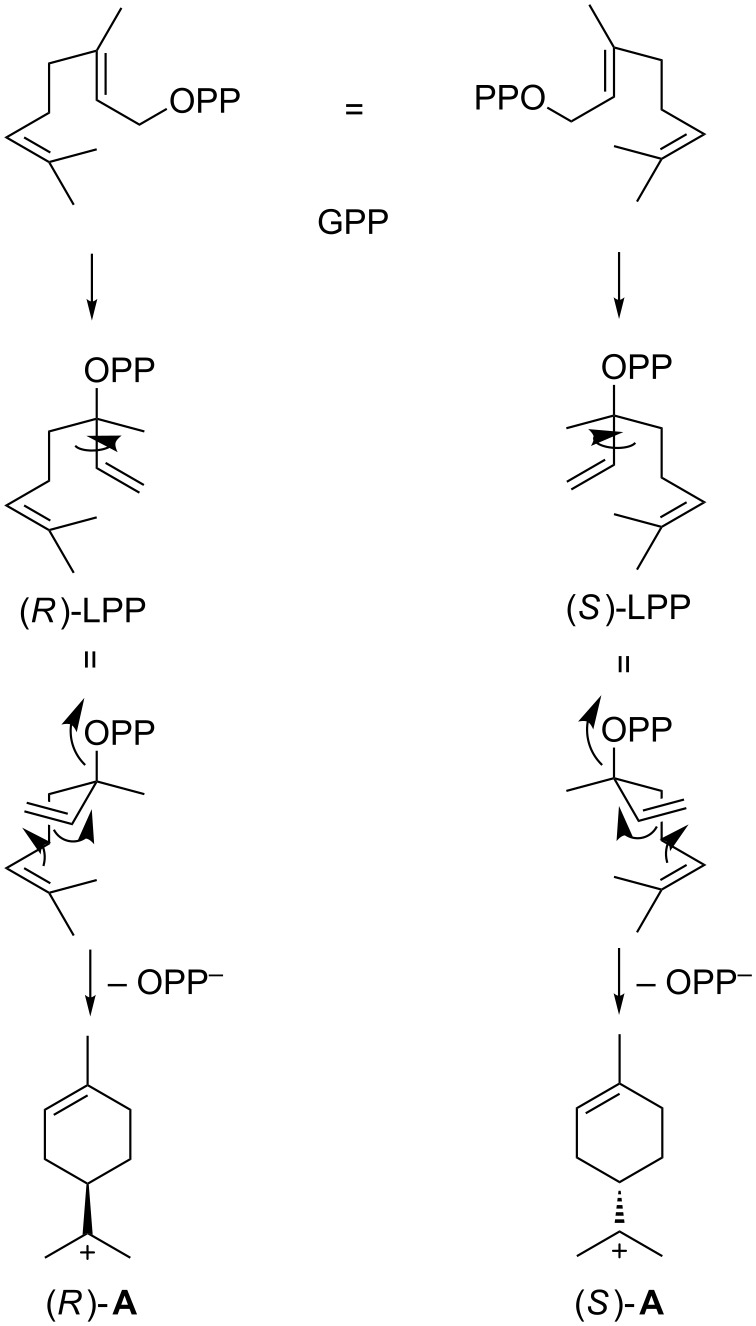
Initial steps of the cyclisation of GPP towards monoterpene products [34]. Both pathways are likely co-occurring in the TS to explain the formation of both enantiomers of **10**, **11** and **13**, with the major enantiomer in all cases being derived from (*R*)-**A**.

GC analysis on a homochiral stationary phase was used to assign the absolute configurations of the observed chiral monoterpenes (*R*)-(+)-limonene (**10**), (*R*)-(+)-α-terpineol (**11**), (+)-α-pinene (**13**) and (*S*)-(+)-linalool (**15**) as shown in Figure 2 by comparison with commercially available standards (Figures S6–S9, Supporting Information File 1). The non-enzymatic degradation of GPP as a background reaction to **15** resulted in a substantial loss of stereoinformation for this compound (7% ee). Also the cyclised products **10**, **11** and **13** where not obtained in enantiomerically pure form (ee values were varying between 64% and 88%, as judged by integration), which may point to different possible binding and folding modes within the TS’s active site for GPP involving both enantiomers of linalyl diphosphate (LPP, Scheme 1) and the terpinyl cation (**A**). Other TSs producing an enantiomeric mixture of monoterpenes are also known, e g., from *Pinus taeda* [33]. However, the major enantiomer of each cyclised monoterpene product described herein was found to be derived from (*R*)-**A**.

Compound **17** was isolated from a large scale incubation of the TS with GGPP and identified by NMR as cembrene A. Chiral phase GC analysis showed also in this case a mixture of enantiomers with the major one being (–)-cembrene A (61% ee), the enantiomer of the product obtained from a cembrene A synthase (CAS) from *Allokutzneria albata* [27], which was used for comparison (Figure S10, Supporting Information File 1).

Taken together, the overall more sluggish conversion of GPP and GGPP by the TS leading to enantiomeric mixtures, the higher biosynthetic complexity of the obtained sesquiterpenes and the absence of spontaneous hydrolysis products in the incubation with FPP compared to the appearance of **14** and **15** in the incubation with GPP and **18** in the experiment with GGPP, this TS from *C. arvum* is characterised as a multiproduct (+)-β-himachalene synthase (HcS) possessing additional mono- and diterpene cyclase activity.

### The structures of its minor products reveal the cyclisation mechanism of HcS

**Scheme 2:**
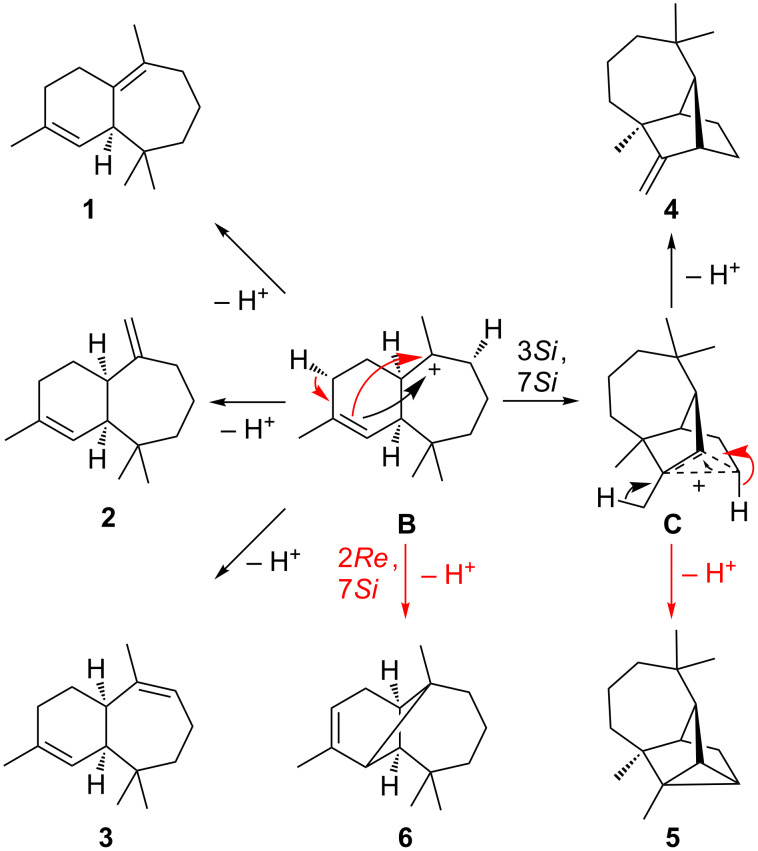
Late stage cyclisations of the himachalyl cation **B** to HcS products **1**–**6**. Alternative mechanistic and reaction arrows belonging to branching points are shown in red.

Since **17** is a simple 1,14-cyclisation product, and all cyclised monoterpenes are derived from the extensively studied terpinyl cation [35–36], this work focusses on elucidating the more interesting sesquiterpene cyclase mechanism of HcS. Most sesquiterpene products **1**–**6** of HcS including the main product **1**, but also α-himachalene (**2**), γ-himachalene (**3**), longifolene (**4**), longicyclene (**5**), and α-longipinene (**6**) are traditionally considered to be derived from the himachalyl cation (**B**) [8,37] (Scheme 2).

**Figure 3:**
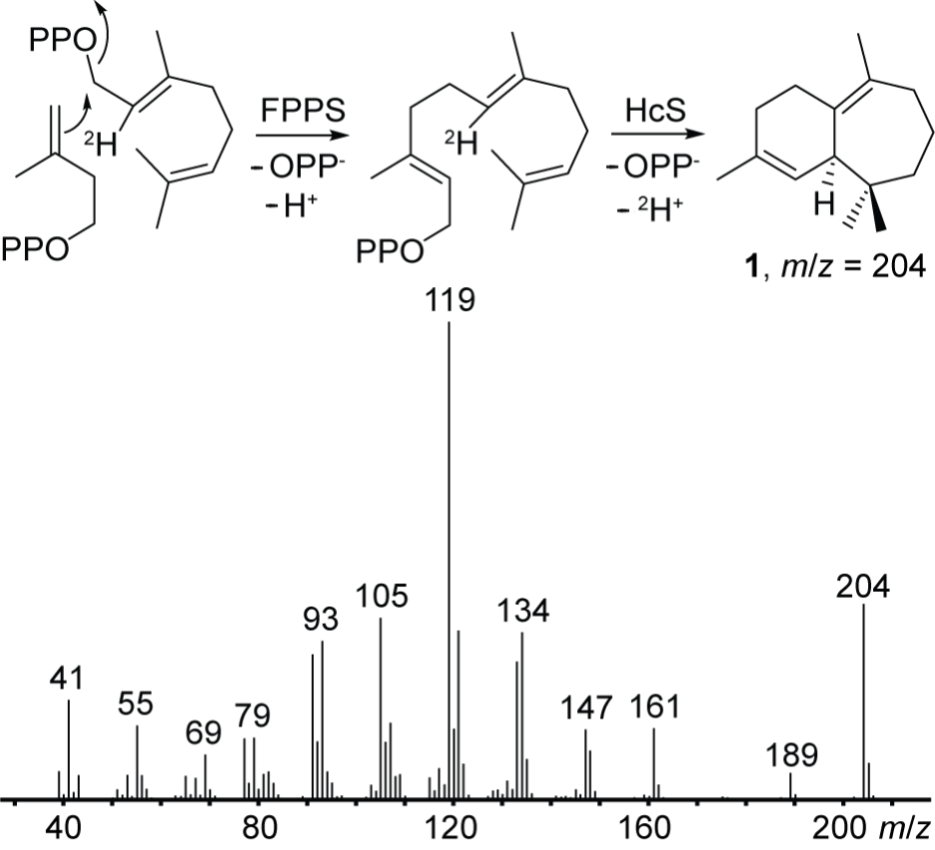
EI mass spectrum of **1** arising from an incubation of (2-^2^H)GPP and IPP with FPPS and HcS showing a loss of label during deprotonation.

Whereas **1**–**3** are simple deprotonation products of **B**, **4** and **5** require a further 3,7-ring closure, leading to the non-classical cation **C**, which is a derivative of the 2-norbornyl cation [38]. This system either collapses by deprotonation at the methyl group to longifolene (**4**), or by deprotonation at C-4 with formation of a cyclopropane ring to longicyclene (**5**). Starting from **B**, a 2,7-ring closure and deprotonation at the same carbon atom gives α-longipinene (**6**). For the main product **1**, the deprotonation was followed by an incubation of HcS and FPPS with (2-^2^H)GPP [39] and IPP, which resulted in unlabelled **1** as observed by GC–MS (Figure 3).

**Figure 4:**
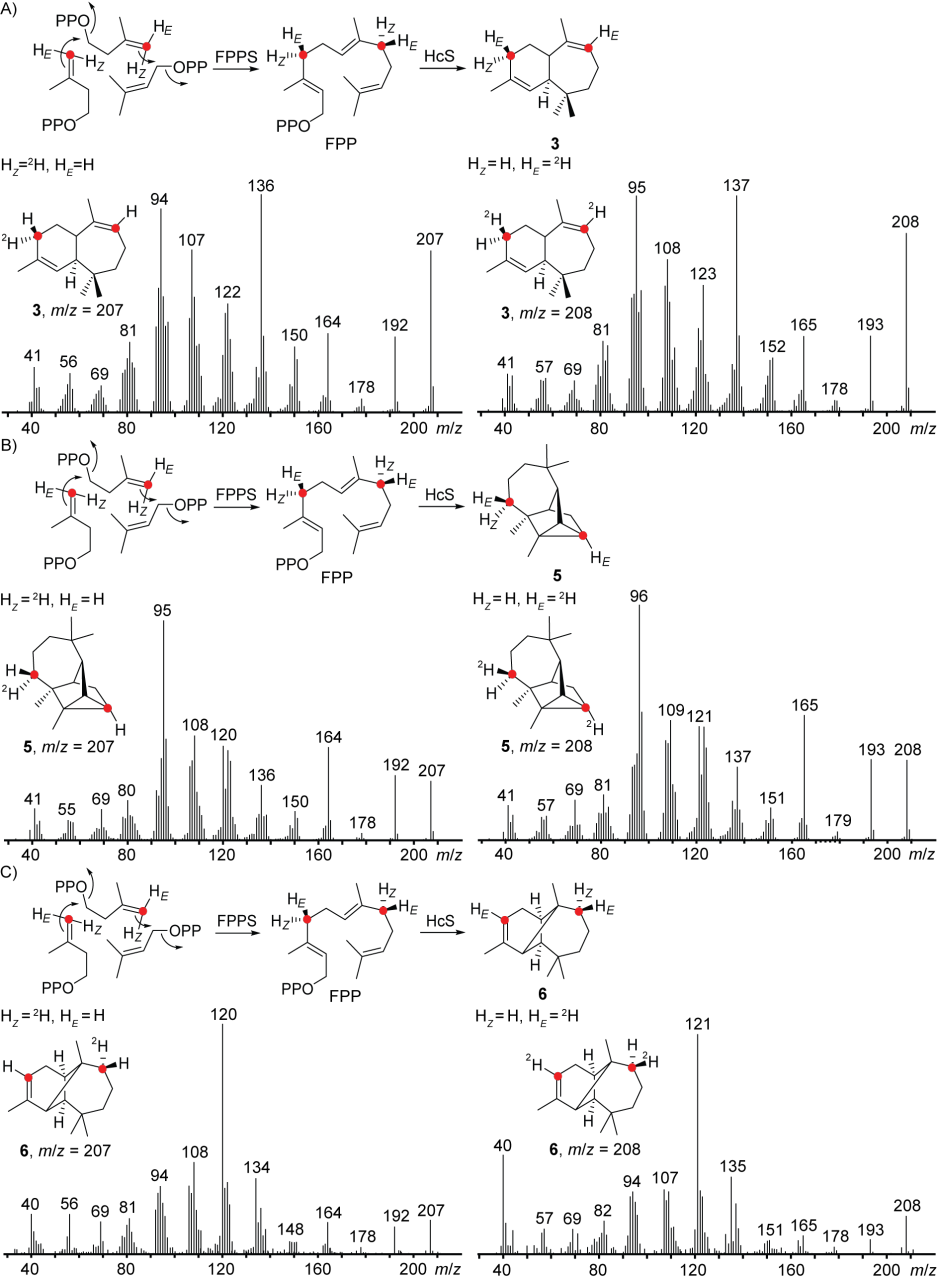
Stereochemical course of the final deprotonation step towards **3**, **5** and **6** investigated by GC–MS. EI mass spectra of labelled A) **3**, B) **5** and C) **6** obtained from the incubation of HcS and FPPS with DMAPP and (*Z*)-(4-^13^C,4-^2^H)IPP (left) or (*E*)-(4-^13^C,4-^2^H)IPP (right) indicating stereospecific loss of one hydrogen atom. ^13^C-Labellings are indicated by red dots.

In case of a deprotonation at a methylene group, relevant for the formation of compounds **3**, **5** and **6**, the stereochemical course of these final steps could be followed by stereoselective deuterations. GC–MS analysis of the products obtained from the incubations with HcS, FPPS, DMAPP and (*Z*)- or (*E*)-(4-^13^C,4-^2^H)IPP showed a specific loss of H*_Z_* in all cases (Figure 4).

**Scheme 3:**
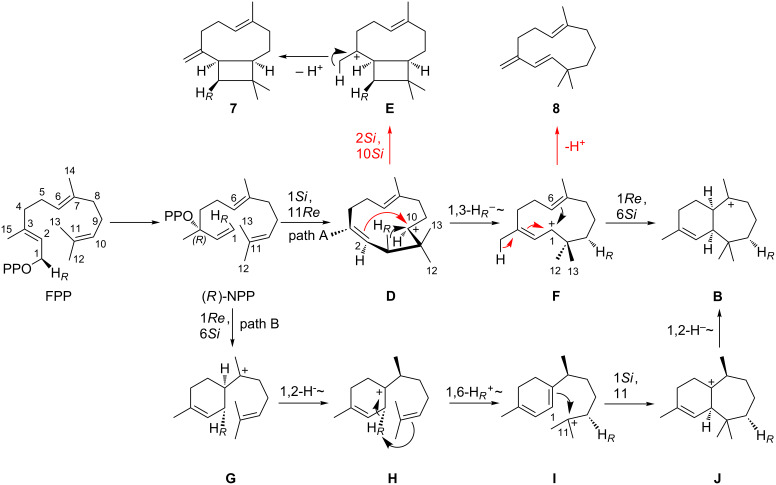
Proposed cyclisation mechanism towards cation **B** via an initial 1,11-cyclisation (path A) and an hypothetical alternative mechanism via an initial 1,6-cyclisation (path B). Alternative mechanistic and reaction arrows belonging to branching points are shown in red.

Intriguingly, all deprotonation steps leading to **1**, **3**, **5** and **6** proceed from the same face of **B**. Giving access to most products, cation **B** can be considered as the central branching point within the HcS catalysed cyclisation mechanism. To rationalise the formation of **B** starting from FPP, two different pathways were initially assumed (Scheme 3). Both start with a 1,3-*syn*-allylic rearrangement of OPP to (*R*)-nerolidyl diphosphate (NPP). This step is usually proposed to generate a (*Z*)-configured C-2,C-3 double bond after cyclisation [40]. Following the first mechanism (path A), a 1,11-cyclisation can yield secondary cation **D**, which either stabilises by 2,10-ring closure to give the caryophyllenyl cation **E** that can be deprotonated at the methyl group to yield 9-*epi*-(*E*)-β-caryophyllene (**7**), or **D** undergoes a 1,3-hydride shift to the allylic cation **F**. Deprotonation leads to γ-humulene (**8**), but a 1,6-ring closure gives access to **B**.

The second shown option, path B, assumes a 1,6-ring closure of (*R*)-NPP to the bisabolyl cation **G**. Proceeding with a 1,2-hydride shift to **H**, the key step is a 1,6-proton shift to give the tertiary cation **I**. This idea is derived from a very similar proton transfer starting from the bisabolyl cation, which occurs in the cyclisation mechanism to trichodiene [41]. A 1,11-cyclisation yields tertiary cation **J**, which can undergo a 1,2-hydride shift to **B**. While path B circumvents the secondary cation intermediate **D**, the HcS products **7** and **8** are hard to explain from path B. Together with the absence of any 1,6-cyclised bisabolene derived molecules in the product mixture their occurrence represent experimental evidence in favour of path A.

### Incubation experiments enlighten the stereochemical course of the 1,11-cyclisation and the 1,3-hydride shift

**Figure 5:**
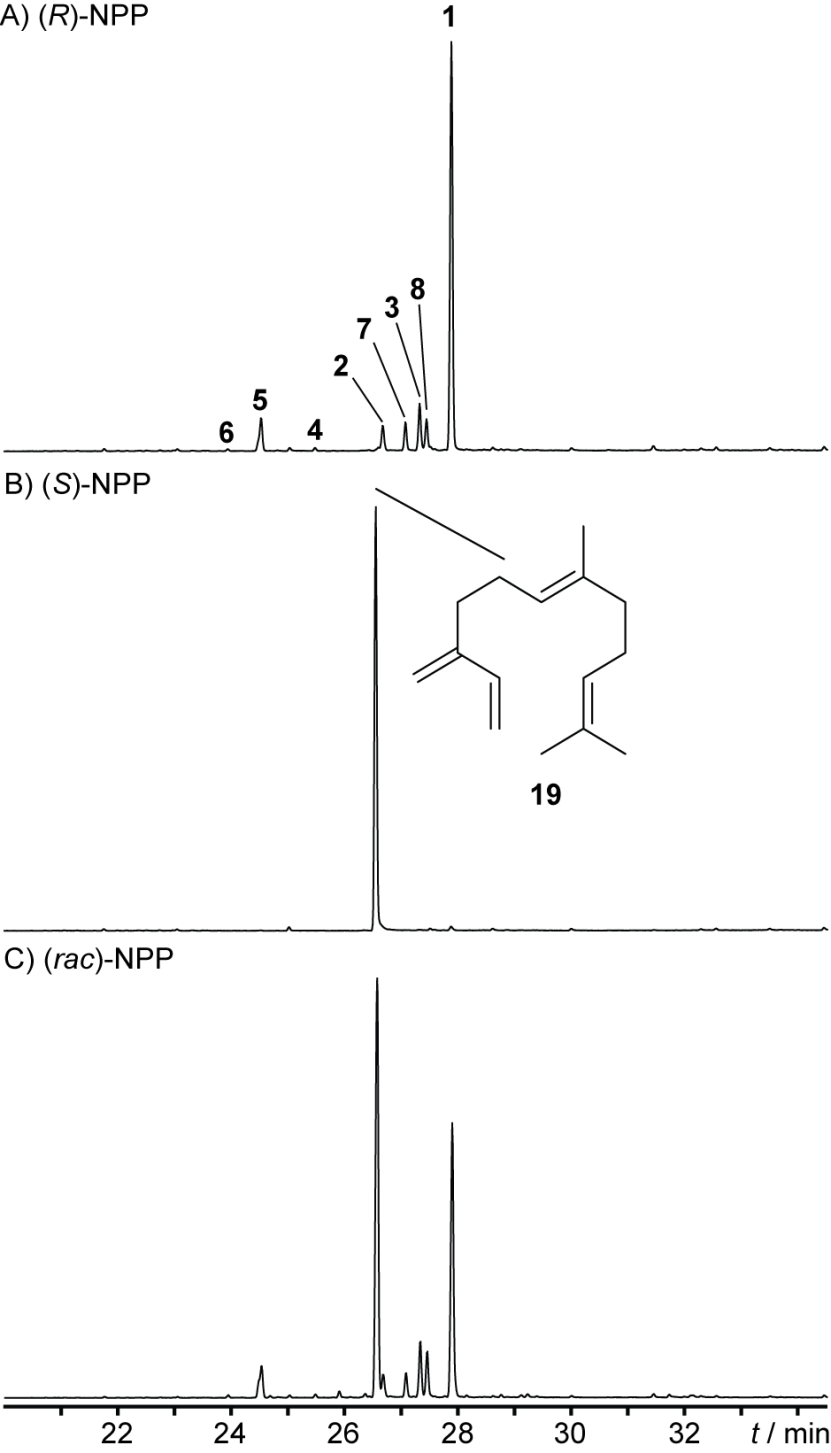
Total ion chromatogram of hexane extracts from HcS incubations with A) (*R*)-NPP, B) (*S*)-NPP and C) (*rac*)-NPP.

Although the general idea of path A appears to be straightforward at first sight, the details proofed to be challenging as deeper insights for the stereochemistry of each step were obtained by incubation experiments. The question, whether (*R*)-NPP or its enantiomer (*S*)-NPP is involved in the HcS catalysed cyclisation cascade, was addressed by incubation of both enantiomerically pure substrates and (*rac*)-NPP [13] with HcS. The resulting chromatograms (Figure 5) clearly demonstrate (*R*)-NPP as an intermediate, which is a substrate for the production of **1**–**8** in approximately the same ratio as with FPP. In contrast, (*S*)-NPP is selectively converted into (*E*)-β-farnesene (**19**, *I* = 1460 (HP-5MS), Lit: *I* = 1459 (HP-5MS) [42]). The same outcome regarding the formation of (*Z*)-γ-bisabolene from (*R*)-NPP and FPP, but of **19** from (*S*)-NPP was recently also observed for BbS [13].

**Figure 6:**
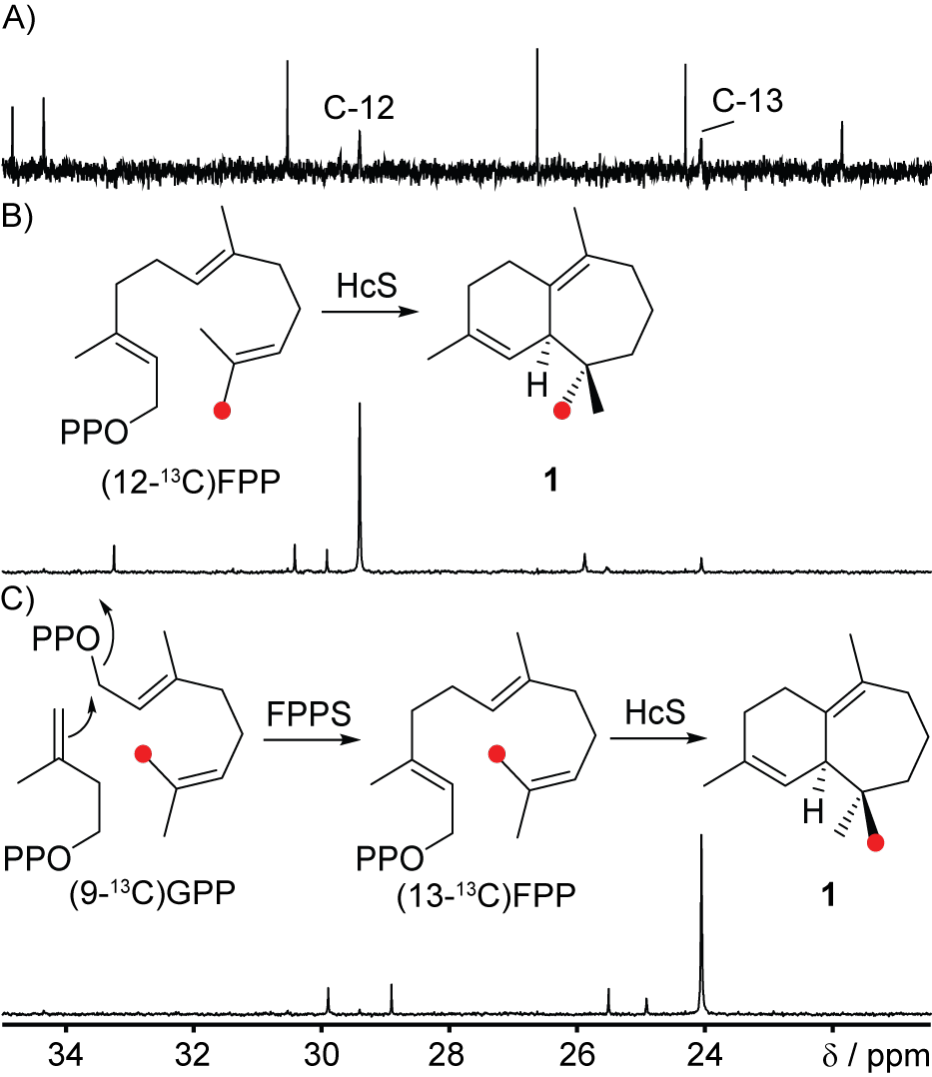
The origin of the two diastereotopic methyl groups in **1**. Partial ^13^C NMR spectrum of A) unlabelled **1**, B) an extract obtained from the incubation of HcS with (12-^13^C)FPP and C) an extract obtained from the incubation of HcS with (9-^13^C)GPP, which is enzymatically converted to (13-^13^C)FPP. Red dots indicate ^13^C labelled carbon atoms.

Targeting the stereochemical course of the 1,11-cyclisation of (*R*)-NPP to cation **D**, (12-^13^C)- [43] and enzymatically prepared (13-^13^C)FPP from (9-^13^C)GPP [39] and IPP with FPPS were incubated with HcS to follow the fate of the geminal methyl groups for **1** (Figure 6). Combined with the relative orientation of each methyl group deduced by NOESY, these experiments showed an 11*Re* attack preceding the formation of **D**. The observed absolute configurations of the monoterpenes **10**, **11** and **13** and of the diterpene **17** support this finding, because their formation requires involvement of the same face of the terminal isoprenoid double bond (6*Si* from GPP and 14*Si* from GGPP). Therefore, a similar binding conformation for the terminal C_5_-unit is reasonable for the three substrates.

**Figure 7:**
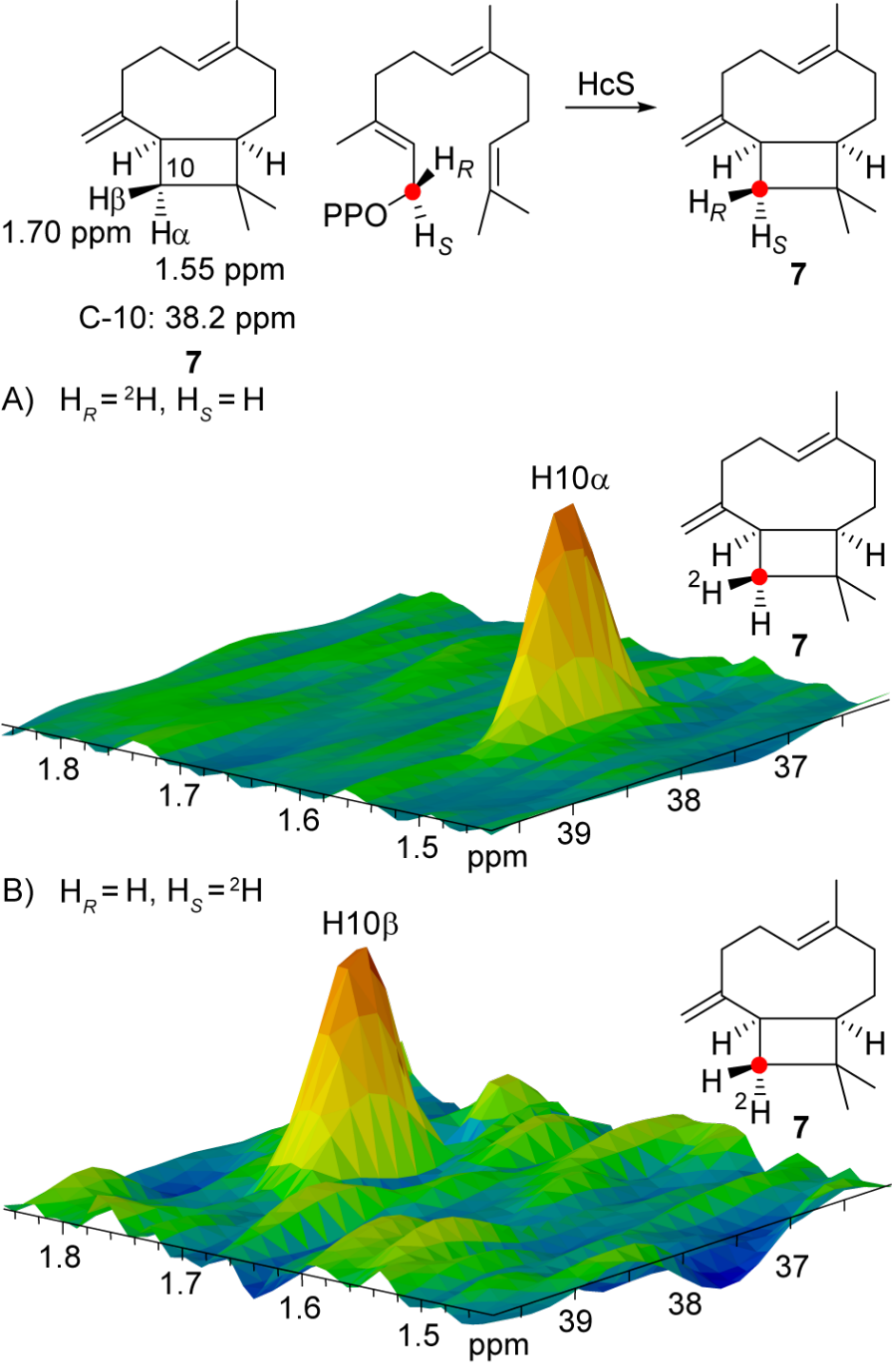
Stereochemical course of the 1,11-cyclisation at C-1 for **7**. Partial HSQC spectra of HcS incubation with A) (1*R*)-(1-^13^C,1-^2^H)FPP and B) (1*S*)-(1-^13^C,1-^2^H)FPP. The reference chemical shifts for **7** are taken from ref. [44]. Red dots represent ^13^C-labelled carbon atoms.

To complete the mechanistic picture of the initial 1,11-cyclisation, also the stereochemical course at C-1 was investigated. Unfortunately, this position is disturbed by the follow-up 1,3-hydride shift in **1** and most products. However, in the side product **7** C-1 remains untouched after 1,11-cyclisation, which allows to investigate the stereochemical course of the first cyclisation step for this compound. First, the absolute configuration of **7** was assigned as shown in Figure 2 from the incubation experiments with (*E*)- and (*Z*)-(4-^13^C,4-^2^H)IPP, DMAPP, FPPS and HcS targeting the positions C-3 and C-7 (Figure S11, Supporting Information File 1), using published NMR data for **7** [44]. The stereochemical fate for the hydrogens at C-1 was then targeted by the incubation of (1*R*)- and (1*S*)-(1-^13^C,1-^2^H)FPP [28] with HcS (Figure 7).

The selective incorporation of deuterium into the diastereotopic positions of **7** is explainable by a 1*Si*,11*Re*-cyclisation of (*R*)-NPP. Given the absolute configuration of NPP and its formation via a 1,3-*syn*-allylic rearrangement from FPP, this ring closure represents an example of a formal *syn*-S_N_2’ reaction. This is an intriguing observation, since for other TSs a NPP-cyclisation by *anti*-S_N_2’ is usually described [40,45–47]. This cyclisation mechanism is thought to be the predominant case, giving rise to a more energetically favoured transition state, but occasionally also the *syn*-stereochemistry was observed [48]. The rather unexpected stereochemical course of the HcS-catalysed cyclisation of NPP found herein therefore shows, that this step has to be investigated for *anti*- versus *syn*-attack experimentally for every single case, especially for a conformationally flexible situation like a 1,11-cyclisation. Intriguingly, the stereochemical course of the initial cyclisation step can even be substrate dependent. The 1,6-cyclisation towards the monoterpenes **10**, **11** and **13** as investigated by the incubation of (1*S*)- and (1*R*)-(1-^13^C,1-^2^H)GPP with HcS and comparison to the NMR data of the commercial available products (Table S4–S8, Supporting Information File 1) clearly obeys the *anti*-S_N_2’ case (Figures S12–S14, Supporting Information File 1). The observation that **15** was obtained as a nearly racemic mixture contrasts the far more selective incorporation of deuterium into the olefinic positions at C-1 of **15** (Figure S15, Supporting Information File 1). This result supports (*R*)-LPP as an intermediate, formed by a 1,3-*syn*-allylic rearrangement to determine the observed stereochemical course at C-1, while the tertiary diphosphate might then undergo a non-enzymatic degradation to explain the high loss of stereoinformation in **15**. Also for the achiral β-myrcene (**14**), an imbalanced incorporation of deuterium is found at C-1 (Figure S16, Supporting Information File 1). With the opposite stereochemical course than for **15**, **14** is likely derived from the minor enantiomer (*S*)-LPP in analogy to **19** observed from (*S*)-NPP. For the diterpene **17** (Table S9, Supporting Information File 1), similar investigations using (1*S*)- and (1*R*)-(1-^13^C,1-^2^H)GGPP [49] with HcS resulted in the expected outcome for a direct 1,14-cyclisation of GGPP (Figure S17, Supporting Information File 1) in line with the results obtained with CAS from *A. albata* for *ent*-**17** [27]. Assuming similar chemical shifts at C-1 for **14** and **18**, the analogous signals for C-1 of **18** gave comparable results with the same stereochemical course as observed for **14**, although with lower preservation of stereoinformation (Figure S18, Supporting Information File 1).

**Figure 8:**
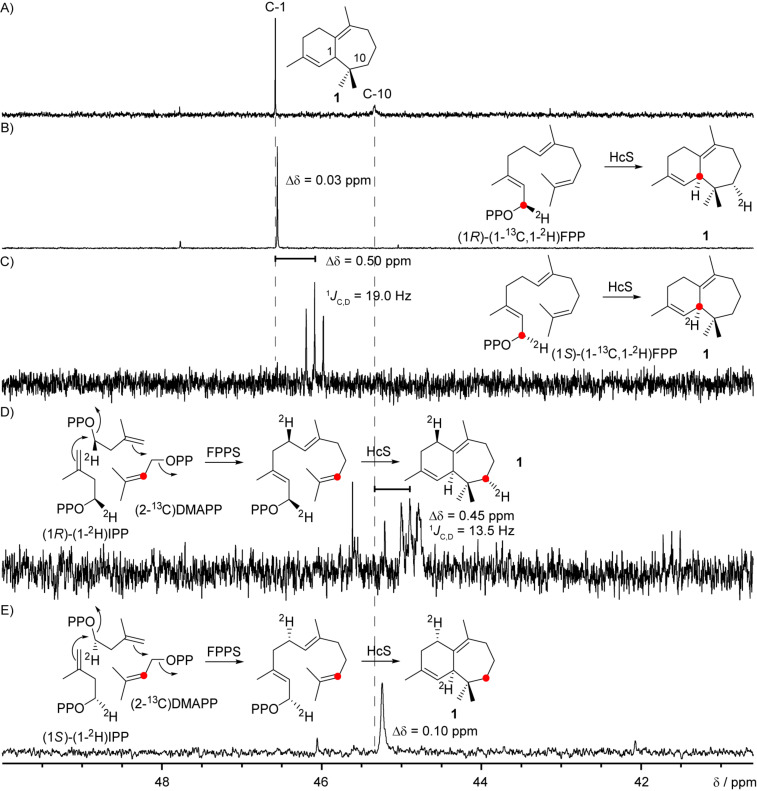
Investigation of the 1,3-hydride shift in the cyclisation towards **1**. Partial ^13^C NMR spectra of A) unlabelled **1**, and incubations with HcS and B) (1*R*)-(1-^13^C,1-^2^H)FPP or C) (1*S*)-(1-^13^C,1-^2^H)FPP compared to incubations of HcS with FPPS, (2-^13^C)DMAPP and D) (1*R*)-(1-^2^H)IPP or E) (1*S*)-(1-^2^H)IPP showing a movement of H*_R_* from C-1 to C-10. The singlets for B) and E) are slightly shifted to higher field (designated as Δδ) because of the deuterium location nearby. ^13^C-Labelled carbon atoms are indicated by red dots. Dashed grey lines show the chemical shifts of the carbon atoms for unlabelled **1**.

To shed light on the stereochemical course of the 1,3-hydride shift connecting cations **D** and **F**, a series of labelling experiments were conducted to determine the origin of the shifting hydrogen (C-1) and its destination (C-10) for **1** (Figure 8). A comparison of the ^13^C NMR spectra from the incubations of HcS with (1*R*)- and (1*S*)-(1-^13^C,1-^2^H)FPP, resulting in a singlet for the (*R*)-case and a triplet in the (*S*)-case indicating a direct C–D bond, clearly demonstrated the stereospecific migration of H*_R_* from C-1. To complete the observations also for C-10, (2-^13^C)DMAPP was synthesised from (2-^13^C)-3-methylbut-2-en-1-ol [43] and incubated with (1*R*)- or (1*S*)-(1-^2^H)IPP [50], FPPS and HcS resulting in the expected opposite outcome than stated above, namely a triplet in the (*R*)-case and a singlet for the (*S*)-sample.

**Figure 9:**
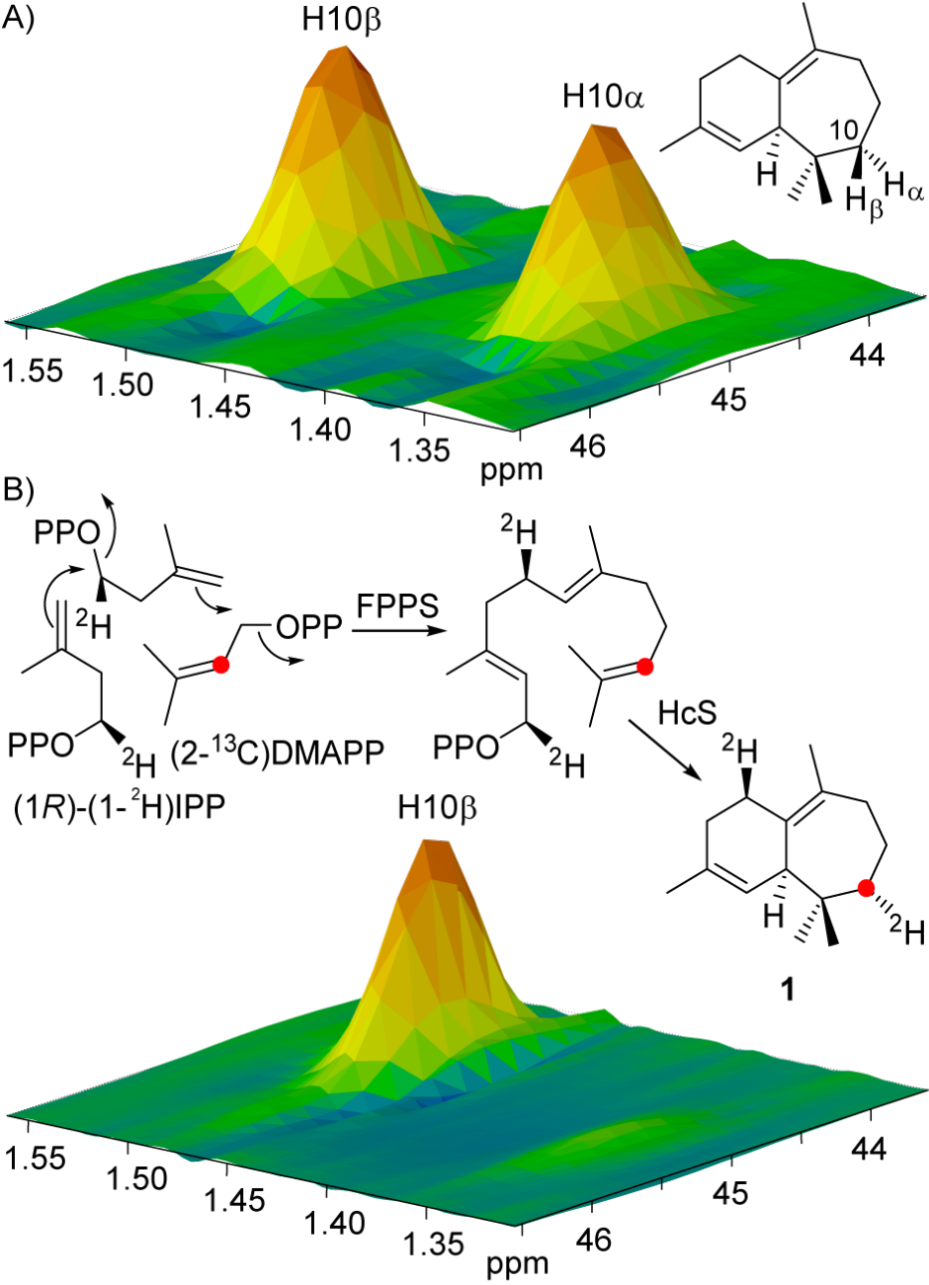
Stereochemical course of the 1,3-hydride shift at C-10 in **1**. Partial HSQC spectra of A) unlabelled **1** and B) labelled **1** arising from the incubation of (1*R*)-(1-^2^H)IPP and (2-^13^C)DMAPP with FPPS and HcS. Red dots indicate ^13^C-labelled carbon atoms.

HSQC analysis of the material obtained from the incubation of (1*R*)-(1-^2^H)IPP and (2-^13^C)DMAPP with FPPS and HcS also allowed for the assignment of the newly introduced diastereotopic position at C-10 (Figure 9). Together with the assignment of the hydrogens by NOESY in **1**, these data show a stereoselective incorporation of H*_R_*-1 into the H_α_-position at C-10 by a vanished crosspeak.

Combining the information deduced from the extensive incubation experiments stated above, a structural model for the reactive conformation of cation **D** is proposed (Figure S19, Supporting Information File 1). This intermediate, or structurally related transition states for the corresponding concerted reactions to avoid its secondary nature, are of central importance in understanding the initial HcS catalysed cyclisation towards cation **B**. The discussed conformation is imprinted by the structure of **7** with its relative conformation at the four-membered ring system allowing for a 2*Si*,10*Si*-cyclisation to **E** without major rotational changes and also reflects the short distance between H*_R_* and C-10 for the 1,3-hydride shift towards the 10*Si* face leading to **F**. Intriguingly, the unusual *syn*-S_N_2’ ring closure from (*R*)-NPP leads to the diphosphate moiety (OPP^−^) being located close to the “backside” of the cyclising molecule, which may give rise to an explanation of the multiproduct nature of HcS. At this location, OPP^−^ can easily abstract “backwards” pointing hydrogen atoms from different positions which reflects the observation of the regio- and stereochemistry of the deprotonations.

### HcS provides access to labelled sesquiterpenes for EIMS fragmentation studies

**Figure 10:**
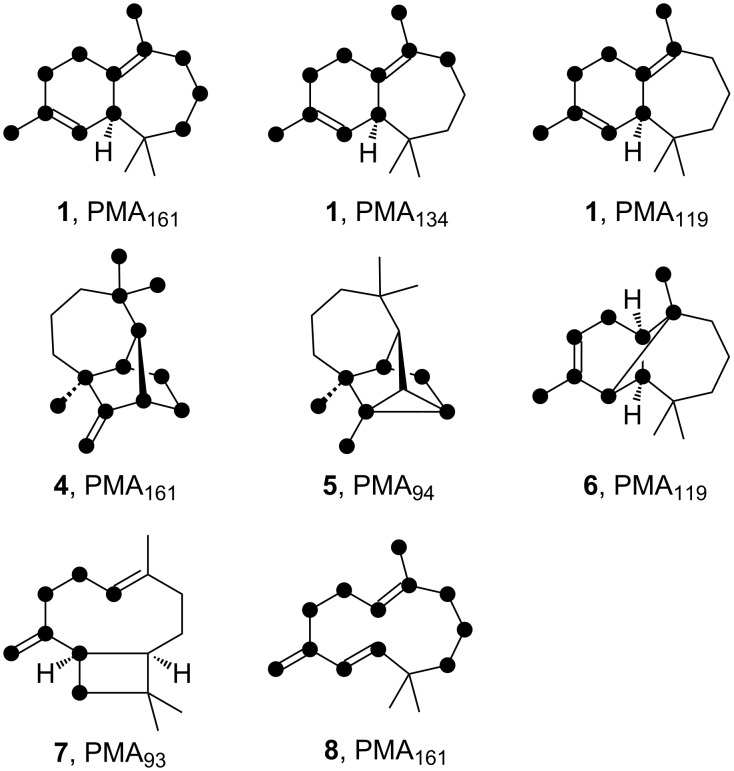
Position specific mass shift analysis for selected EIMS ions of HcS products. Black dots represent an increased mass of the ion (*m*/*z* = +1) in case of a ^13^C-labelling in this position. Proposed fragmentation mechanisms for these ions are presented in Schemes S1–S3 (Supporting Information File 1).

Since HcS produces a mixture of structurally interesting sesquiterpenes, its synthetic abilities were also exploited to study EIMS fragmentation mechanisms. Therefore, all fifteen singly-^13^C labelled FPP isotopomers, either obtained by synthesis or enzymatically [39,43,51], were converted with HcS to result in mixtures of specifically labelled **1**–**8**. The incorporation of label into **1** was checked by ^13^C NMR (Figure S20, Supporting Information File 1) and all samples were analysed by GC–MS. This allowed for the assignment of carbon positions to specific EI-fragments of the corresponding mass spectrum by observing an increase of +1 Da, if the labelled position is part of the fragment (position specific mass shift analysis, PMA [41,52–53]). Although for many fragments multiple overlaying fragmentation pathways were observed, some of them showed clear position dependent results, which are summarised in Figure 10. The EI mass spectra for each position and molecule laying the basis for the presented three fragments for **1** together with one fragment each for **4**–**8** are depicted in Figures S21–S26 (Supporting Information File 1). Possible EI-fragmentation mechanisms connected to them are discussed in Schemes S1–S3 (Supporting Information File 1).

## Conclusion

In summary, a new terpene synthase from *C. arvum* was characterised as a multiproduct (+)-β-himachalene synthase. Accepting GPP, FPP and GGPP, HcS is a promiscuous enzyme, whose catalysis suffers from poor selectivity. Nevertheless, the formation of multiple sesquiterpene products demands for a challenging mechanistic model, which was refined by extensive labelling experiments. Several interesting details were disclosed including the stereochemical course of a 1,3-hydride migration from C-1 to C-10 and the 1,11-cyclisation featuring the unusual *syn*-S_N_2’ attack. Combining various aspects of the initial cyclisation, the proposed conformer of cation **D** may also rationalise the reduced selectivity of HcS by its positioning of OPP^−^. Providing access to labelled isotopomers of its products, including structurally demanding polycyclic terpenes, HcS also served as a platform for investigating selected aspects of their EIMS fragmentation mechanisms. The labelling experiments performed with HcS described in this study therefore represent an encouragement to experimentally explore and elucidate every stereochemical detail of a terpene cyclisation mechanism for a comprehensive picture of the complex reactions, these amazing enzymes are able to catalyse.

## Supporting Information

Experimental details for gene cloning, gene expression, protein purification, incubation experiments with isotopically labelled precursors, preparative scale incubation and synthesis of (2-^13^C)DMAPP. The amino acid sequence of HcS, a phylogenetic tree of bacterial terpene synthases, SDS-PAGE analysis of the recombinant protein, listed NMR data for **1** and **9**, labelling experiments for the determination of the absolute configurations of **1** and **7**, chiral phase GC analysis of **10**, **11**, **13**, **15** and **17**, labelling experiments for the stereochemical course at C-1 of the monoterpenes and diterpenes, a graphical model for cation **D**, NMR spectra for the incubations of singly labelled FPPs with HcS, EIMS data for compounds **1** and **4**-**8** arising from these incubations and discussion of fragmentation mechanisms for selected ions.

File 1 Additional material.
