# Supplementary material for: Mechanistic investigations on multiproduct β-himachalene synthase from Cryptosporangium arvum
Source: Beilstein J Org Chem. 2019 May 2;15:1008–19. doi: 10.3762/bjoc.15.99 (PMC6541374; doi:10.3762/bjoc.15.99)
Supplement: File 1 — Additional material. [file Beilstein_J_Org_Chem-15-1008-s001.pdf]

BEILSTEIN JOURNAL OF ORGANIC CHEMISTRY

Supporting Information

for

Mechanistic investigations on multiproduct β-himachalene synthase from *Cryptosporangium arvum*

Jan Rinkel and Jeroen S. Dickschat

Beilstein J. Org. Chem. **2019,** *15,* 1008–1019. doi:10.3762/bjoc.15.99

Additional material

License and Terms: This is a supporting information file under the terms of the Creative Commons Attribution License (http://creativecommons.org/licenses/by/4.0). Please note that the reuse, redistribution and reproduction in particular requires that the authors and source are credited.
The license is subject to the *Beilstein Journal of Organic Chemistry* terms and conditions: (https://www.beilstein-journals.org/bjoc)

```
VPRDPVPALYCPLPTRMHPESGPIGRRSVAWLERFGLLDDDRTRERIRATRPAEWACRIAPGGTGAGL
QLVSDWTHLGFVIDDTRFDAGPIADRPGRLIPLMMRLMHALDHPEAPADDDPFAAAFRNLSARARAGA
PAAVVRRWADGNLEWFFSVACLTAHRVAGTMPTLTEYLNLGPRDRAMRLTGSLIELAEGSVLTDADRE
EPTVRAVTQAANLLVTIGNDLFSFRKEADDDVLESNIVGVIRHRDGGTAEAALARTVELHDRVMCLYL
GLRAQLDEDGSSTVRRHLTRLDHLIRGNLEWSVRVPRYGTGSASPWAETPADGRAAAPHLVPIDWWWD
QLRTA
```

Figure S1: Amino acid sequence of HcS (accession number WP_035852539, gene locus tag CRYAR_RS18680). Conserved residues and motifs are highlighted in yellow.

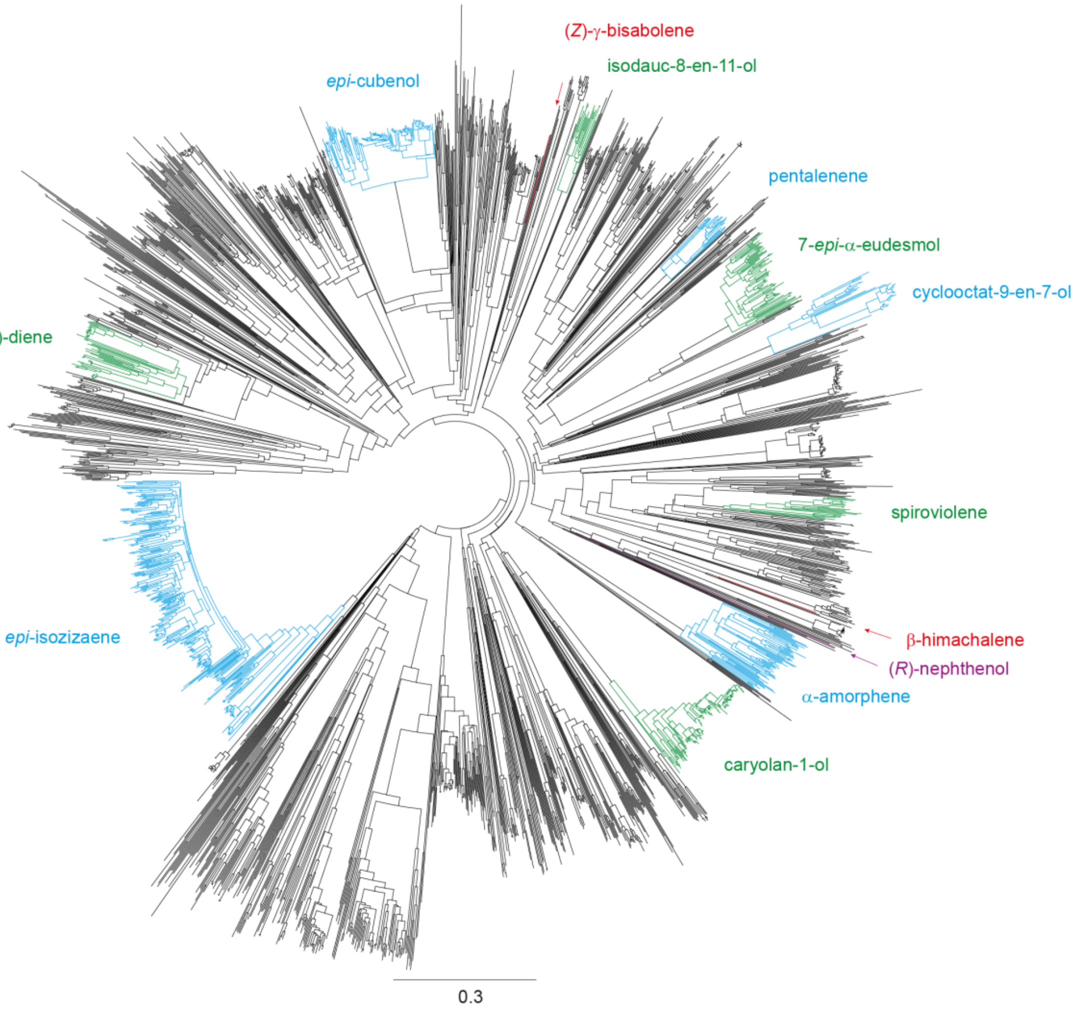

Figure S2: The locations of HcS and BbS [1] from *Cryptosporangium arvum* (coloured in red) in a phylogenetic tree of bacterial terpene synthases (synthases for geosmin and 2-methylisoborneol are omitted). The closest characterised enzyme to HcS is (*R*)-nepthenol synthase [2] (coloured in purple) from *Streptomyces* sp. SANK 60404 with 27% identity.

Strains and culture conditions

Cryptosporangium arvum DSM 44712 was obtained from the DSMZ (Braunschweig, Germany) and was grown in gym 65 medium (10.0 g malt extract, 4.0 g glucose, 4.0 g yeast extract, 1 L water, pH 7.2) at 28 °C. *Escherichia coli* BL21(DE3) was grown in LB medium (10.0 g tryptone, 5.0 g yeast extract, 5.0 g NaCl, 1 L H_2O, pH 7.2) at 37 °C.

Gene cloning

Gene cloning of the β-himachalene synthase (HcS) gene (WP_035852539) from genomic DNA of *C. arvum* into pYE-Express [3] was done as previously described for the β-bisabolene synthase (BbS) from the same organism [1] using the primers JR128f and JR128r (Table S1) for the first PCR and a second set of primers JR129f and JR129r for the addition of homology arms.

Table S1: Primers used for gene cloning.

Primer	Sequence[a]
JR128f	GTGCCGCGTGATCCGGTAC
JR128r	CTAAGCCGTACGCAGCTGGTC
JR129f	<u>GGCAGCCATATGGCTAGCATGACTGGTGGA</u>GTGCCGCGTGATCCGGTAC
JR129r	<u>TCTCAGTGGTGGTGGTGGTGGTGCTCGAGT</u>CTAAGCCGTACGCAGCTGGTC

[a]: Homology arms to pYE-express are underlined.

Gene expression and protein purification

The *E. coli* BL21(DE3) strain harbouring the plasmid pYE-HcS were grown in LB precultures (5 mL) with kanamycin (50 μg/mL final concentration) at 37 °C overnight. The preculture (1/1000) was then transferred to main cultures in LB medium with kanamycin at the same concentration and the cells were grown at 37 °C with shaking until OD_{600} = 0.4–0.6 was reached. After cooling the cultures to 18 °C, IPTG (0.4 mM final concentration) was added to induce expression. The cultures were shaken at 18 °C overnight and harvested by centrifugation (8000*g*, 35 min, 4 °C). After the medium was discarded and the cell pellets were suspended in binding buffer (10 mL/L culture; 20 mM Na_2HPO_4, 500 mM NaCl, 20 mM imidazole, 1 mM $MgCl_2$, pH = 7.4, 4 °C), the cells were lysed by ultra sonification on ice (50% power, 5 × 1 min). The resulting suspension was centrifuged (14600*g*, 3 × 7 min, 4 °C) and the soluble protein fractions were filtrated and transferred to a Ni^{2+}-NTA affinity chromatography column (Super Ni-NTA Resin, Generon, Slough, UK). The protein was washed with binding buffer (2 × 10 mL/L culture) and desorbed from the material with elution buffer (2 × 6.25 mL/L culture; 20 mM Na_2HPO_4, 500 mM NaCl, 500 mM imidazole, 1 mM $MgCl_2$, pH = 7.4, 4 °C). SDS-PAGE was used for analysis of the protein containing fractions (Figure S3), which were used for incubation experiments. Typical protein concentrations in the complete elution fraction using this procedure were 3.1 mg/mL, as determined by Bradford assay [4] calibrated to bovine serum albumin.

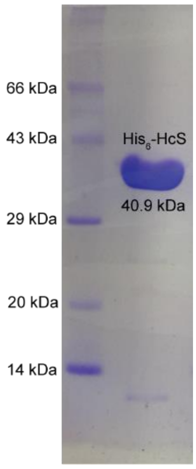

Figure S3: SDS-PAGE analysis of purified recombinant HcS with its predicted molecular weight.

GC–MS analysis

An Agilent (Santa Clara, CA, USA) 7890B GC, which was connected to a 5977A mass detector using a HP5-MS fused silica capillary column (30 m, 0.25 mm i. d., 0.50 μm film) was used to record GC/MS data. GC parameters were 1) inlet pressure: 77.1 kPa, He at 23.3 mL min^{-1}, 2) injection volume: 2 μL or 1 μL, 3) temperature program: 5 min at 50 °C, then increasing at 5 °C min^{-1} or 10 °C min^{-1} to 320 °C, 4) 60 s valve time, and 5) carrier gas: He at 1.2 mL min^{-1}. MS parameters were 1) source: 230 °C, 2) transfer line: 250 °C, 3) quadrupole: 150 °C and 4) electron energy: 70 eV.

Chiral GC analysis

To record GC data on a homochiral stationary phase, an Agilent GC 7820A GC system equipped with an FID detector and an Agilent Cyclosil-B capillary column (30 m, 0.25 mm inner diameter, 0.25 µm film) was utilised. Inlet temperature: 250 °C, injection volume: 1 μL, carrier gas: H_2 @ 2.3 mL/min. For analysis of limonene (**10**) and for α-pinene (**13**), the GC was programmed as follows: starting from 50 °C, increasing with 1 °C/min to 75 °C, then further increasing with 40 °C/min to 245 °C while holding this temperature for 1 min. For analysis of α-terpineol (**11**) and for linalool (**15**), the GC was programmed as follows: starting from 80 °C, increasing with 1 °C/min to 110 °C, then further increasing with 40 °C/min to 245 °C while holding this temperature for 1 min. Finally, for analysis of cembrene A (**17**), the temperature program was changed to: starting from 120 °C, increasing by 0.1 °C to 132 °C, then further increasing with 40 °C/min to 245 °C while holding this temperature for 1 min. The commercially available reference materials (+)- and (–)-**10**, (–)-**11**, (+)- and (–)-**13** were obtained from Sigma-Aldrich (St. Louis, MO, USA). (–)-**15** was purchased from Acros Organics (Thermo Fisher Scientific, Geel, Belgium).

NMR spectroscopy

The instruments Bruker (Billerica, MA, USA) Avance I (500 MHz), Avance III HD Prodigy (500 MHz) or an Avance III HD Cryo (700 MHz) were used to record NMR data. The obtained spectra were referenced against solvent signals (^{1}H NMR, residual proton signals: C_6D_6 δ = 7.16 ppm, D_2O δ = 4.79 ppm; ^{13}C NMR: C_6D_6 δ = 128.06 ppm) [5].

Incubation experiments with recombinant HcS and terpenoid precursors

The terpene cyclase activity of HcS was tested in four experiments by dissolving the diphosphates GPP, FPP, GGPP and GFPP (1 mg each) in substrate buffer (1 mL; 25 mM NH_4HCO_3). Binding buffer (3.5 mL) and incubation buffer (5 mL; 50 mM Tris/HCl, 10 mM $MgCl_2$, 20% glycerol, pH 8.2) were added and the reaction was started by adding HcS elution fraction (0.5 mL). The incubations were shaken at 28 °C for 3 h and extracted with hexane (200 μL). The organic layer was dried with $MgSO_4$ and directly analysed by GC–MS. Whereas no product was observed from GFPP, the incubations with GPP, FPP and GGPP yielded cyclised terpene products (Figure 1 of main text).

Preparative scale incubation of HcS with FPP

FPP (150 mg) was dissolved in substrate buffer (20 mL) and the solution was dropped within 2 h to a slowly stirred mixture of incubation buffer (300 mL), water (150 mL) and HcS elution fraction (150 mL) arising from a 12 L *E. coli* expression culture. The reaction was incubated with shaking overnight at 28 °C and extracted with pentane (3 × 100 mL). The combined organic layers were dried with $MgSO_4$ and concentrated under reduced pressure to yield β-himachalene (**1**; 2.1 mg) and the auto-oxidation product γ-dehydro-*ar*-himachalene (**9**; 0.5 mg) as colourless oils after column chromatography on silica gel [pentane].

(+)-β-himachalene (**1**). $[\alpha]_D^{25}$ = +32.9 (c = 0.4, $CHCl_3$) [Lit: $[\alpha]_D^{25}$ = +213 (c = 0.05, $CHCl_3$)] [6]. TLC [pentane]: R_f = 0.91. GC (HP-5MS): I = 1507 [Lit: I = 1503 (HP-5)] [7]. MS (EI, 70 eV): *m*/*z* (%) = 204 (50), 189 (6), 161 (16), 147 (15), 134 (35), 121 (36), 119 (100), 105 (35), 93 (30), 91 (26), 79 (11), 69 (7), 55 (11), 41 (12), see Figure S21. NMR data are listed in Table S2.

γ-dehydro-*ar*-himachalene (**9**). TLC [pentane]: R_f = 0.69. GC (HP-5MS): I = 1535 [Lit: I = 1538 (HP-5)] [8]. MS (EI, 70 eV): *m*/*z* (%) = 200 (82), 185 (100), 171 (73), 157 (76), 143 (37), 128 (24), 115 (14), 105 (5), 91 (6), 77 (8). NMR data are listed in Table S3.

Table S2: NMR data of β-himachalene (**1**) in C_6D_6 recorded at 298 K, cf. literature data [6].

C[a]		^{1}H[b]	^{13}C[b]
1	CH	1.99 (s)	46.6
2	CH	5.56 – 5.53 (m)	123.0
3	C_q	–	134.9
4	CH_2	1.92 – 1.89 (m, 2H)	30.5
5	CH_2	2.64 (ddd, $^{2}J_{H,H}$ = 13.9, $^{3}J_{H,H}$ = 4.0, 4.0, H_α)	26.6
		2.02 – 1.95 (m, H_β)	
6	C_q	–	131.6
7	C_q	–	129.1
8	CH_2	2.47 – 2.42 (m, H_α)	34.3
		1.85 – 1.81 (m, H_β)	
9	CH_2	1.53 – 1.48 (m, H_α)	21.8
		1.46 – 1.40 (m, H_β)	
10	CH_2	1.53 – 1.48 (m, H_β)	45.3
		1.40 – 1.35 (m, H_α)	
11	C_q	–	34.8
12	CH_3	1.02 (s, 3H)	29.4
13	CH_3	0.86 (s, 3H)	24.0
14	CH_3	1.73 – 1.71 (m, 3H)	20.4
15	CH_3	1.73 – 1.71 (m, 3H)	24.3

[a] Carbon numbering as shown in Figure 2 of main text. [b] Chemical shifts δ in ppm, multiplicity: s = singlet, d = doublet, m = multiplet, coupling constants J are given in Hertz.

Table S3: NMR data of γ-dehydro-*ar*-himachalene (**9**) in C_6D_6 recorded at 298 K.

C[a]		^{1}H[b]	^{13}C[b]
1	C_q	–	147.6
2	CH	7.28 – 7.26 (m)	126.6
3	C_q	–	136.0
4	CH	6.99 (dm, $^{3}J_{H,H}$ = 7.8)	126.7
5	CH	7.22 (d, $^{3}J_{H,H}$ = 7.8)	128.2
6	C_q	–	138.4
7	C_q	–	137.7
8	CH	5.78 (tq, $^{3}J_{H,H}$ = 6.7, $^{4}J_{H,H}$ = 1.5)	126.5
9	CH_2	1.97 – 1.92 (m, 2H)	26.6
10	CH_2	1.91 – 1.88 (m, 2H)	48.2
11	C_q	–	38.2
12,13	CH_3	1.32 (s, 6H)	31.6
14	CH_3	2.07 – 2.06 (m, 3H)	23.8
15	CH_3	2.23 (s, 3H)	21.6

[a] Carbon numbering as shown in Figure 2 of main text. [b] Chemical shifts δ in ppm, multiplicity: s = singlet, d = doublet, t = triplet, q = quartet, m = multiplet, coupling constants *J* are given in Hertz.

General procedure for incubation experiments with isotopically labelled substrates and recombinant HcS

For determination of the absolute configuration, (*E*)- and (*Z*)-(4-^{13}C,4-^{2}H)IPP [9] (1 mg) or (*R*)- and (*S*)-(1-^{13}C,1-^{2}H)GPP [10] (1 mg) were dissolved in substrate buffer (1 mL) and a solution of DMAPP (0.5 mg) in case of the IPPs or IPP (1 mg) in case of the GPPs in substrate buffer (1 mL) was added. The mixture was diluted with binding buffer (1 mL) and incubation buffer (5 mL). The reaction was started by adding FPP synthase [11] (FPPS, *S. coelicolor*) elution fraction (1 mL) and HcS elution fraction (1 mL). The mixture was incubated for 3 h at 28 °C with shaking and extracted with C_6D_6 (650 μL; 350 μL). The combined organic layers were analysed by GC–MS and NMR.

For $^{13}C_1$-labelling, the ^{13}C-labelled diphosphates (0.5 mg; (9-^{13}C)- [12] and (10-^{13}C)GPP [13]; (1-^{13}C)-, (2-^{13}C)-, (3-^{13}C)-, (4-^{13}C)-, (5-^{13}C)-, (6-^{13}C)-, (7-^{13}C)-, (8-^{13}C)-, (9-^{13}C)-, (10-^{13}C)-, (11-^{13}C)-, (12-^{13}C) and (15-^{13}C)FPP [14]) were dissolved in substrate buffer (1 mL). A solution of IPP in substrate buffer (1 mL; 2 mg/mL) in case of labelled GPPs or only substrate buffer (1 mL) in case of labelled FPPs was added. To this mixture, incubation buffer (5 mL) was added, FPPS elution fraction (1 mL, labelled GPPs) or elution buffer (1 mL, labelled FPPs) and HcS elution fraction (2 mL). The mixtures were incubated for 3 h at 28 °C and then directly extracted with C_6D_6 (650 μL, 350 μL). The combined organic layers were analysed by GC–MS and NMR.

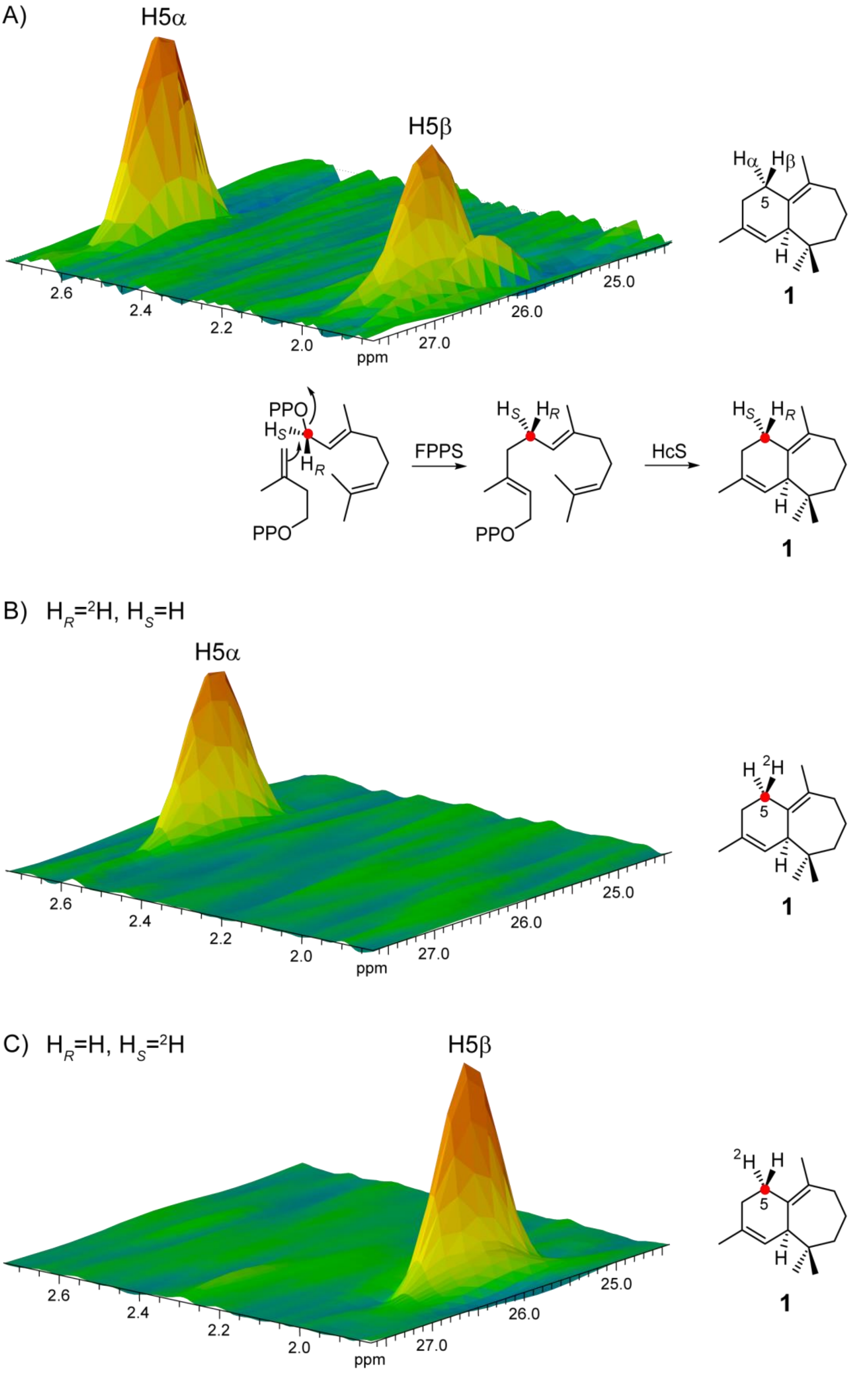

Figure S4: Determination of the absolute configuration of **1** by labelling experiments for C-5. Partial HSQC spectra of A) unlabelled **1**, and incubation of HcS, FPPS and IPP with B) (1*R*)-(1-^{13}C,1-^{2}H)GPP or C) (1*S*)-(1-^{13}C,1-^{2}H)GPP showing selective incorporation of deuterium into the diastereotopic hydrogen positions of the C-5 methylene group. With the NOE-based assignment of its relative configuration, these results are in line with the absolute configuration shown. Red dots represent ^{13}C-labelled carbon atoms.

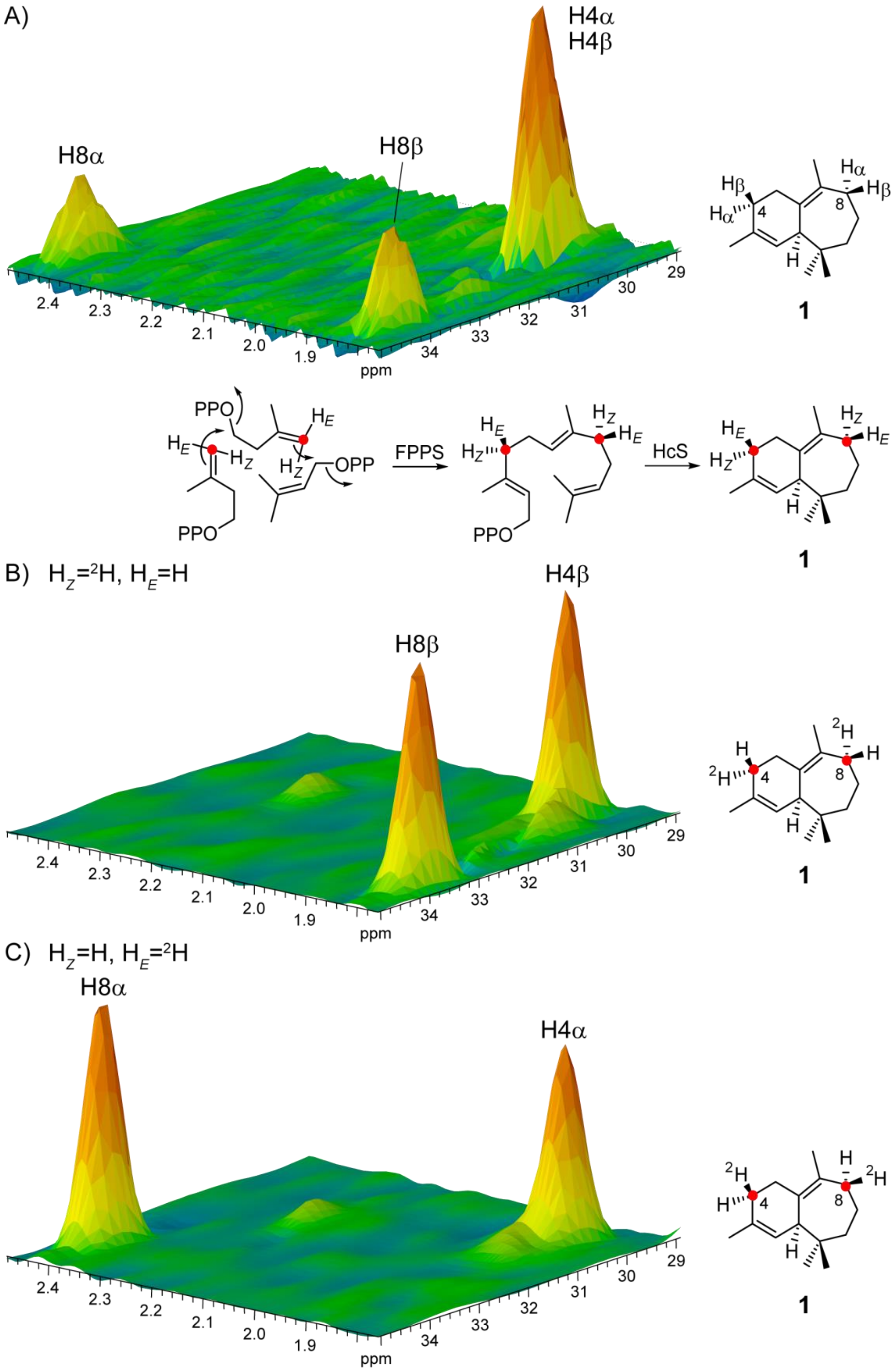

Figure S5: Determination of the absolute configuration of **1** by labelling experiments for C-4 and C-8. Partial HSQC spectra of A) unlabelled **1**, and incubation of HcS, FPPS and DMAPP with B) (*Z*)-(4-^{13}C,4-^{2}H)IPP or C) (*E*)-(1-^{13}C,1-^{2}H)IPP showing selective incorporation of deuterium into the diastereotopic hydrogen positions of the methylene groups. With the NOE-based assignment of their relative configurations, these results are in line with the absolute configuration shown. Red dots represent ^{13}C-labelled carbon atoms.

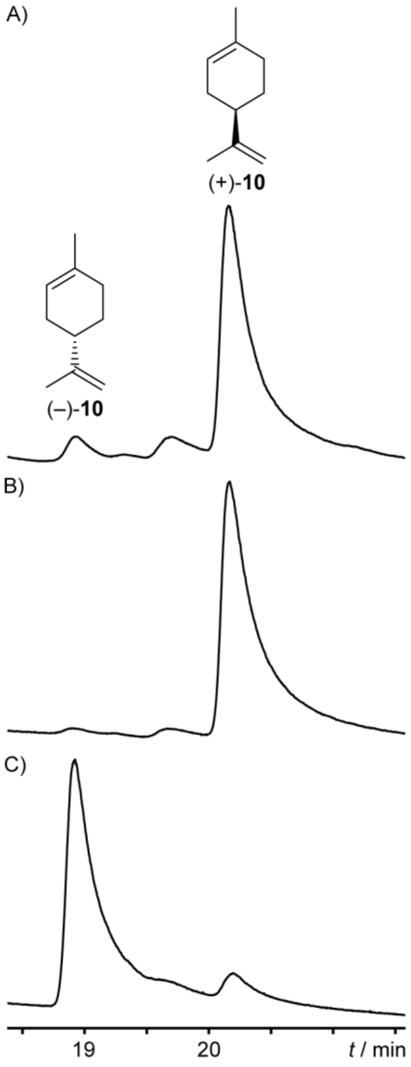

Figure S6: Determination of the absolute configuration of HcS produced limonene (**10**). Chiral GC chromatogram of A) HcS incubation with GPP, B) (+)-**10** and C) (–)-**10**. Integration of both peaks results in an enantiomeric excess of 88%.

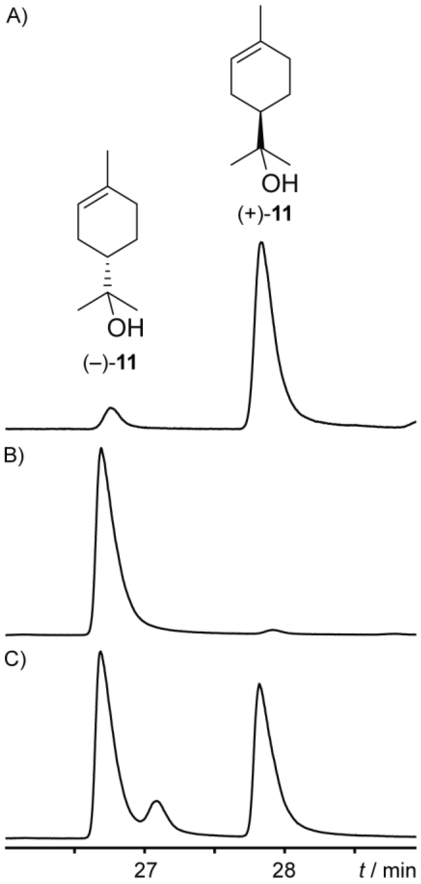

Figure S7: Determination of the absolute configuration of HcS produced α-terpineol (**11**). Chiral GC chromatogram of A) HcS incubation with GPP, B) (–)-**11** and C) commercially available mixture of α-terpineol isomers (unknown enantiomeric composition). Integration of both peaks results in an enantiomeric excess of 84%.

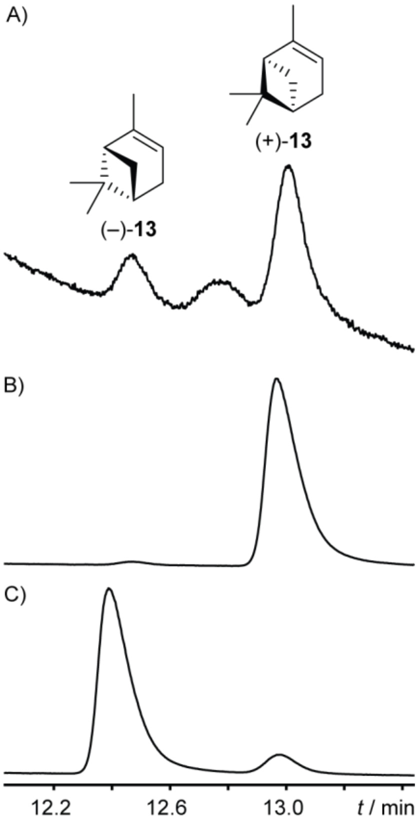

Figure S8: Determination of the absolute configuration of HcS produced α-pinene (**13**). Chiral GC chromatogram of A) HcS incubation with GPP, B) (+)-**13** and C) (–)-**13**. Integration of both peaks results in an enantiomeric excess of 64%.

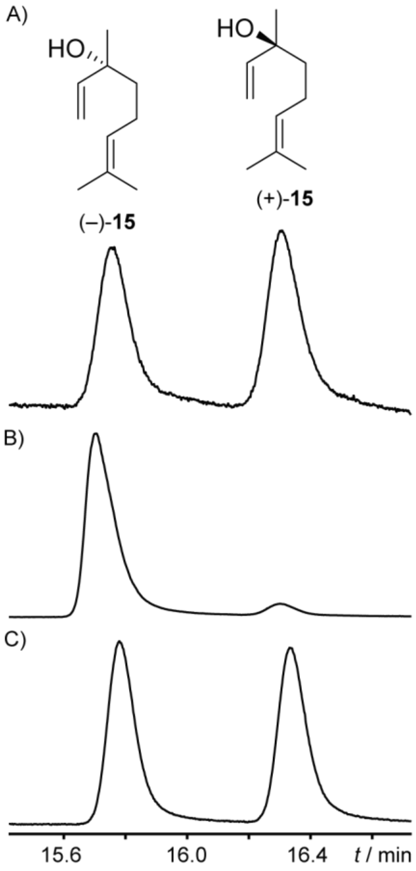

Figure S9: Determination of the absolute configuration of HcS produced linalool (**15**). Chiral GC chromatogram of A) HcS incubation with GPP, B) (–)-**15** and C) (*rac*)-**15** obtained by the literature known Grignard addition of vinylmagnesium bromide to sulcatone [15]. Integration of both peaks results in an enantiomeric excess of 7% in favour for (*S*)-(+)-**15**. **15** is also frequently observed as a non-enzymatic degradation product of GPP. As discussed in main text, the high loss of stereoinformation can be rationalised by the enzymatic formation of (*R*)-LPP with a defined stereochemical course (cf. Figure S15) and the subsequent non-enzymatic degradation of LPP to **15**.

Preparative scale incubation of HcS with GGPP

Comparable to the reaction with FPP described above, GGPP (110 mg) was dissolved in substrate buffer (20 mL) and the solution was dropped within 2 h to a slowly stirred mixture of incubation buffer (200 mL), water (120 mL) and HcS elution fraction (60 mL). The reaction was incubated with shaking at 28 °C for 4 h and extracted with pentane (3 × 100 mL). The combined organic layers were dried with $MgSO_4$ and concentrated under reduced pressure to yield cembrene A (**17**; 1.0 mg) as a colourless oil after column chromatography on silica gel [pentane].

(–)-(*R*)-cembrene A (**17**). $[\alpha]_D^{20}$ = –5.4 (*c* = 0.11, $CHCl_3$) [Lit: $[\alpha]_D^{20}$ = –12 (*c* = 0.85, $CHCl_3$)] [16], *ee* = 61% as approximated by chiral GC (Figure S10). NMR data were identical to those reported for **17** [17], which are listed in Table S8.

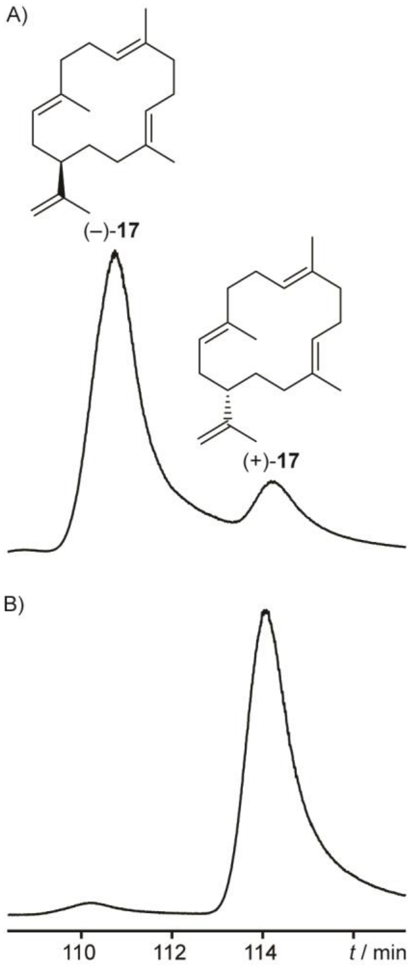

Figure S10: Determination of the absolute configuration of HcS produced cembrene A (**17**). Chiral GC chromatogram of A) **17** isolated from an incubation of HcS with GGPP, B) (+)-**17** isolated from an incubation of cembrene A synthase (CAS) from *Allokutzneria albata* [16]. Integration of both peaks results in an enantiomeric excess of 61%.

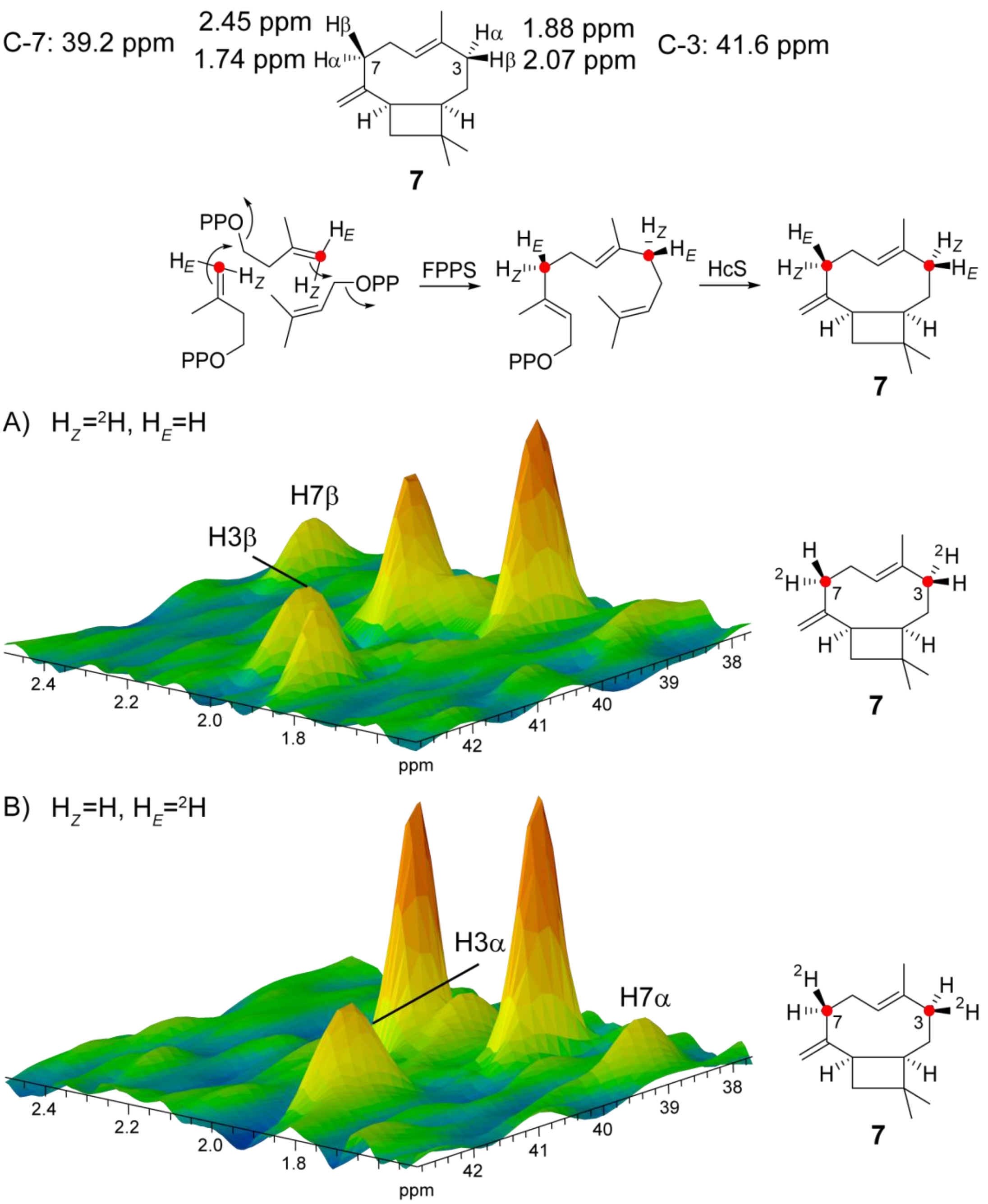

Figure S11: Determination of the absolute configuration of **7** by labelling experiments for C-3 and C-7. Partial HSQC spectra resulting from the incubation of HcS, FPPS and DMAPP with A) (*Z*)-(4-^{13}C,4-^{2}H)IPP or B) (*E*)-(1-^{13}C,1-^{2}H)IPP showing selective incorporation of deuterium into the diastereotopic hydrogen positions of the methylene groups. Together with the NOE-based assignment of their relative configurations with their chemical shifts shown above, which were taken from ref. [18], these results are in line with the absolute configuration shown. Red dots represent ^{13}C-labelled carbon atoms.

Incubation experiments using (*R*)-, (*S*)-, and (*rac*)-NPP with HcS

The NPPs [1] (approx. 0.05 mg each) were dissolved in substrate buffer (100 μL) and added to a mixture of HcS elution fraction (0.4 mL) and incubation buffer (0.5 mL). The reaction mixtures were incubated for 30 min at 28 °C and extracted with hexane (200 μL). The organic phases were dried with $MgSO_4$ and analysed by GC–MS (Figure 5).

Synthesis of (2-^{13}C)DMAPP

Following a literature known procedure [19], (2-^{13}C)-3-methylbut-2-en-1-ol [14] (65 mg, 0.71 mmol, 1.0 equiv) was dissolved in THF (2.4 mL). The solution was cooled to 0 °C and PBr_3 (96 mg, 0.36 mmol, 0.5 equiv) was added. Stirring was continued for 1 h at the same temperature, before ice-cold water (5 mL) was added and the mixture was quickly extracted with Et_2O (3 × 5 mL). The combined organic phases were dried with $MgSO_4$ and carefully concentrated under reduced pressure. The crude product was dissolved in MeCN (4.7 mL) and $(NBu_4)_3HP_2O_7$ (769 mg, 0.85 mmol, 1.2 equiv) was added. The reaction was stirred overnight at room temperature, the mixture was concentrated under reduced pressure and dissolved in ion exchange buffer (1 mL; 25 mM NH_4HCO_3, 2% iPrOH/H_2O). The solution was passed through a DOWEX 50WX8 cation exchange column (NH_4^+-form) with 1.5 column volumes of ion exchange buffer. The resulting solution was lyophilised to yield (2-^{13}C)DMAPP (162 mg, 0.66 mmol, 93%) as a white solid.

^{1}H-NMR (500 MHz, D_2O): δ = 5.41 (dm, $^1J_{H,C}$ = 155.9 Hz, 1H, CH), 4.44 – 4.38 (m, 2H, CH_2), 1.73 (d, $^3J_{H,C}$ = 5.7 Hz, 3H, CH_3), 1.69 (d, $^3J_{H,C}$ = 4.9 Hz, 3H, CH_3) ppm. ^{13}C-NMR (126 MHz, D_2O): δ = 139.9 (d, $^1J_{C,C}$ = 72.4 Hz, C_q), 119.8 (d, $^3J_{C,P}$ = 8.2 Hz, CH*), 62.5 (m, CH_2), 24.9 (d, $^2J_{C,C}$ = 3.3 Hz, CH_3), 17.3 (d, $^2J_{C,C}$ = 1.4 Hz, CH_3) ppm. ^{31}P-NMR (202 MHz, D_2O: δ = -6.77 (d, $^2J_{P,P}$ = 21.9 Hz), -10.24 (dd, $^2J_{P,P}$ = 21.9 Hz, $^3J_{P,C}$ = 8.2 Hz) ppm.

The following listed NMR data were recorded using commercial available materials for comparison of the results obtained by labelling experiments.

Table S4: NMR data of limonene (**10**) in C_6D_6 recorded at 298 K.

C[a]		^{1}H[b]	^{13}C[b]
1	CH_2	2.08 – 2.04 (m, H_α)	31.2
		1.97 – 1.90 (m, H_β)	
2	CH	5.41 – 5.39 (m)	121.2
3	C_q	–	133.5
4	CH_2	1.95 – 1.88 (m, H_α)	30.8
		1.85 – 1.80 (m, H_β)	
5	CH_2	1.74 – 1.70 (m, H_α)	28.3
		1.43 – 1.39 (m, H_β)	
6	CH	2.10 – 2.06 (m)	41.4
7	C_q	–	150.0
8[c]	CH_2	4.82 – 4.79 (m, 2H)	109.0
9[c]	CH_3	1.65 – 1.64 (m, 3H)	20.9
10	CH_3	1.62 – 1.60 (m, 3H)	23.7

[a] Carbon numbering as shown in Figure 2 of main text. [b] Chemical shifts δ in ppm, multiplicity: m = multiplet. [c] Positions may be interchanged.

Table S5: NMR data of α-terpineol (**11**) in C_6D_6 recorded at 298 K.

C[a]		^{1}H[b]	^{13}C[b]
1	CH_2	2.01 – 1.96 (m, H_α)	27.3
		1.77 – 1.71 (m, H_β)	
2	CH	5.42 – 5.40 (m)	121.4
3	C_q	–	133.6
4	CH_2	1.94 – 1.89 (m, H_α)	31.4
		1.87 – 1.83 (m, H_β)	
5	CH_2	1.81 – 1.77 (m, H_α)	24.3
		1.17 (dddd, J = 12.3, 12.3, 12.3, 5.4, H_β)	
6	CH	1.37 (dddd, J = 12.3, 12.3, 5.0, 2.4)	45.3
7	C_q	–	71.9
8[c]	CH_3	1.00 (s, 3H)	27.6
9[c]	CH_3	1.00 (s, 3H)	26.5
10	CH_3	1.64 – 1.63 (m, 3H)	23.6

[a] Carbon numbering in analogy to **10** as shown in Figure 2 of main text. [b] Chemical shifts δ in ppm, multiplicity: s = singlet, d = doublet, m = multiplet, coupling constants J are given in Hertz. [c] Positions may be interchanged.

Table S6: NMR data of α-pinene (**13**) in C_6D_6 recorded at 298 K.

C[a]		^{1}H[b]	^{13}C[b]
1	CH_2	2.32 (ddd, *J* = 8.5, 5.8, 5.5, H_β)	31.9
		1.27 (d, *J* = 8.5, H_α)	
2	CH	1.90 (ddd, *J* = 5.6, 5.6, 1.5)	47.4
3	C_q	–	144.6
4	CH	5.23 – 5.21 (m)	116.6
5	CH_2	2.26 – 2.21 (m, H_α)	31.6
		2.19 – 2.14 (m, H_β)	
6	CH	2.04 – 2.01 (m)	41.2
7	C_q	–	38.2
8[c]	CH_3	1.24 (s, 3H)	26.5
9[c]	CH_3	0.91 (s, 3H)	21.0
10	CH_3	1.64 – 1.63 (m, 3H)	23.2

[a] Carbon numbering in analogy to **10** as shown in Figure 2 of main text. [b] Chemical shifts δ in ppm, multiplicity: s = singlet, d = doublet, m = multiplet, coupling constants *J* are given in Hertz. [c] Positions may be interchanged.

Table S7: NMR data of β-myrcene (**14**) in C_6D_6 recorded at 298 K.

C[a]		^{1}H[b]	^{13}C[b]
1	CH_2	5.21 (d, *J* = 17.9, H_Z)	113.1
		4.97 (d, *J* = 9.7, 1.6, H_E)	
2	CH	6.37 (dd, *J* = 17.6, 10.9)	139.5
3	C_q	–	146.5
4	CH_2	2.26 – 2.19 (m, 2H)	31.9
5	CH_2	2.26 – 2.19 (m, 2H)	27.2
6	CH	5.21 – 5.17 (m)	124.7
7	C_q	–	131.6
8	CH_3	1.66 – 1.65 (m, 3H)	25.8
9	CH_3	1.51 (br s, 3H)	17.7
10	CH_2	4.99 – 4.97 (m, 2H)	116.0

[a] Carbon numbering in analogy to **10** as shown in Figure 2 of main text. [b] Chemical shifts δ in ppm, multiplicity: s = singlet, d = doublet, m = multiplet, coupling constants *J* are given in Hertz.

Table S8: NMR data of linalool (**15**) in C_6D_6 recorded at 298 K.

C[a]		^1H[b]	^{13}C[b]
1	CH_2	5.20 (dd, *J* = 17.3, 1.6, H_Z)	111.4
		4.95 (dd, *J* = 10.7, 1.6, H_E)	
2	CH	5.75 (dd, *J* = 17.3, 10.7)	145.7
3	C_q	–	73.0
4	CH_2	1.50 (ddd, *J* = 13.6, 10.8, 5.8)	42.6
		1.45 (ddd, *J* = 13.6, 10.9, 5.4)	
5	CH_2	2.14 – 2.08 (m)	23.2
		2.08 – 2.01 (m)	
6	CH	5.20 – 5.16 (m)	125.2
7	C_q	–	131.3
8	CH_3	1.65 – 1.64 (m, 3H)	25.8
9	CH_3	1.55 (br s, 3H)	17.7
10	CH_3	1.12 (s, 3H)	28.3

[a] Carbon numbering in analogy to **10** as shown in Figure 2 of main text. [b] Chemical shifts δ in ppm, multiplicity: s = singlet, d = doublet, m = multiplet, coupling constants *J* are given in Hertz.

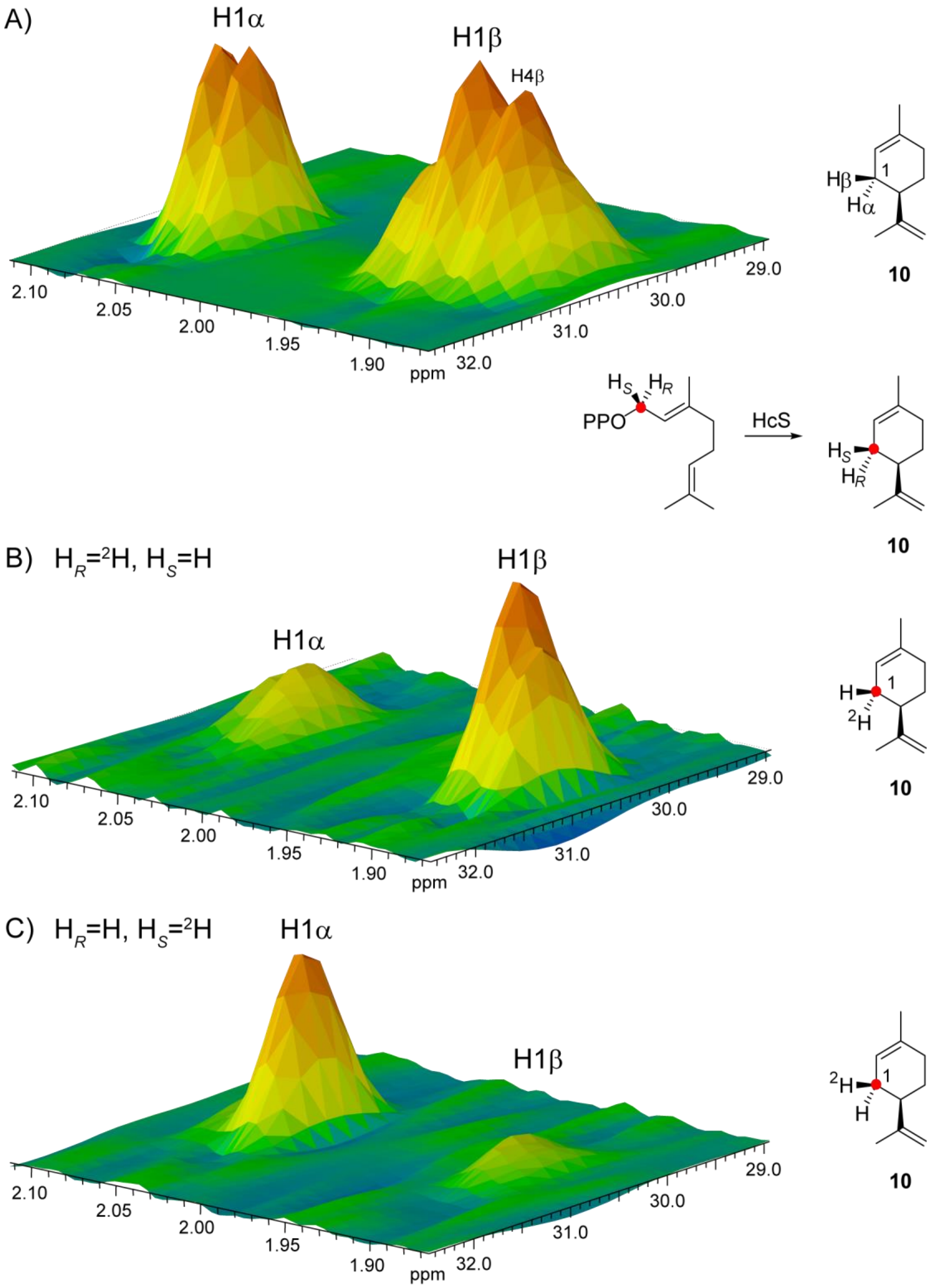

Figure S12: Investigation of the stereochemical course during the HcS catalysed formation of **10**. Partial HSQC of A) unlabelled **10**, B) an incubation of HcS with (1*R*)-(1-^{13}C,1-^{2}H)GPP and C) an incubation of HcS with (1*S*)-(1-^{13}C,1-^{2}H)GPP. The assignment of the α– and β–position is based on NOE correlations. HcS produces a mixture of enantiomers, which is reflected in significantly enlarged minor signals for B) and C). For clarity, only the major enantiomer is shown in the structures.

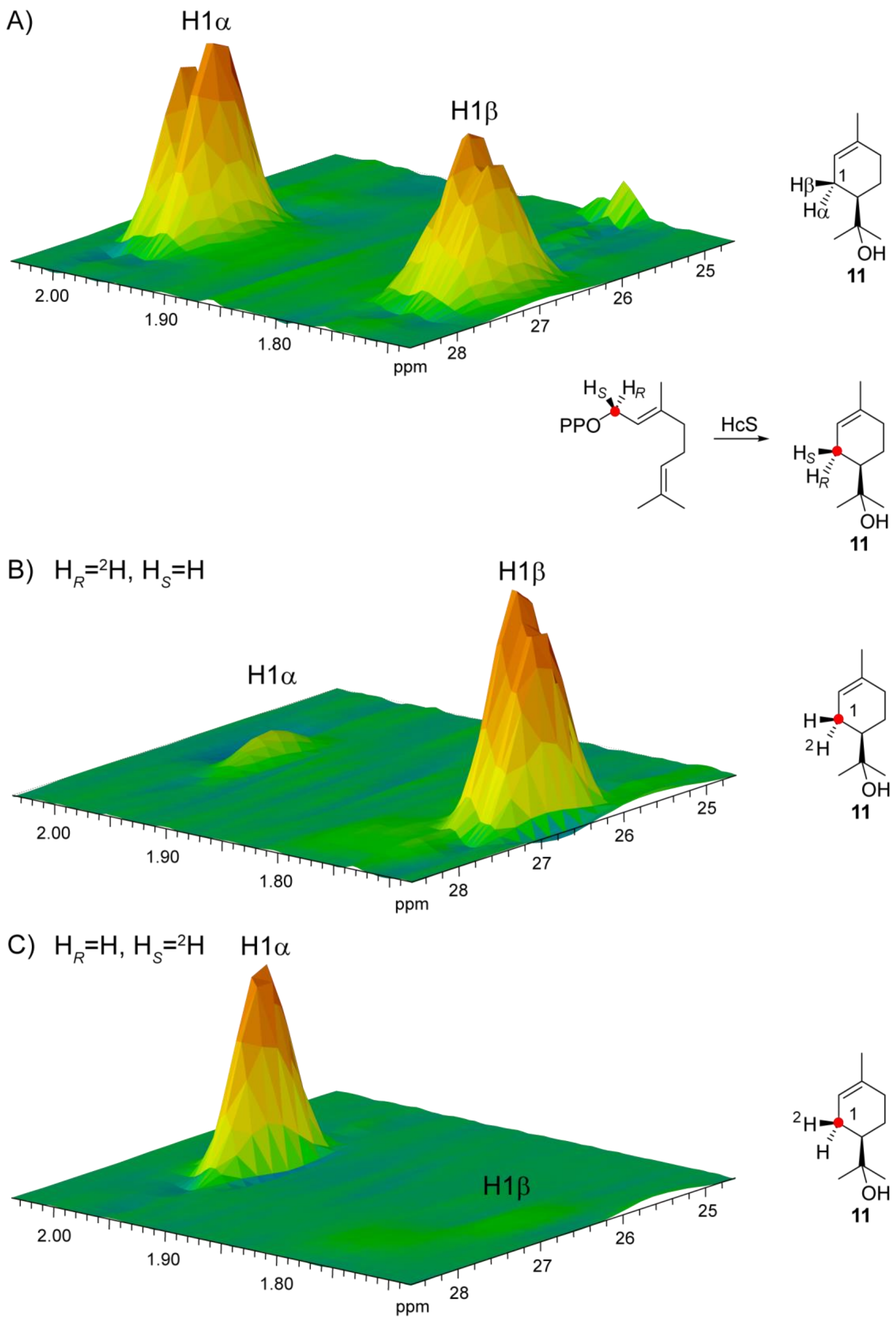

Figure S13: Investigation of the stereochemical course during the HcS catalysed formation of **11**. Partial HSQC of A) unlabelled **11**, B) an incubation of HcS with (1*R*)-(1-^{13}C,1-^{2}H)GPP and C) an incubation of HcS with (1*S*)-(1-^{13}C,1-^{2}H)GPP. The assignment of the α– and β–position is based on NOE correlations. HcS produces a mixture of enantiomers, which is reflected in significantly enlarged minor signals for B) and C). For clarity, only the major enantiomer is shown in the structures.

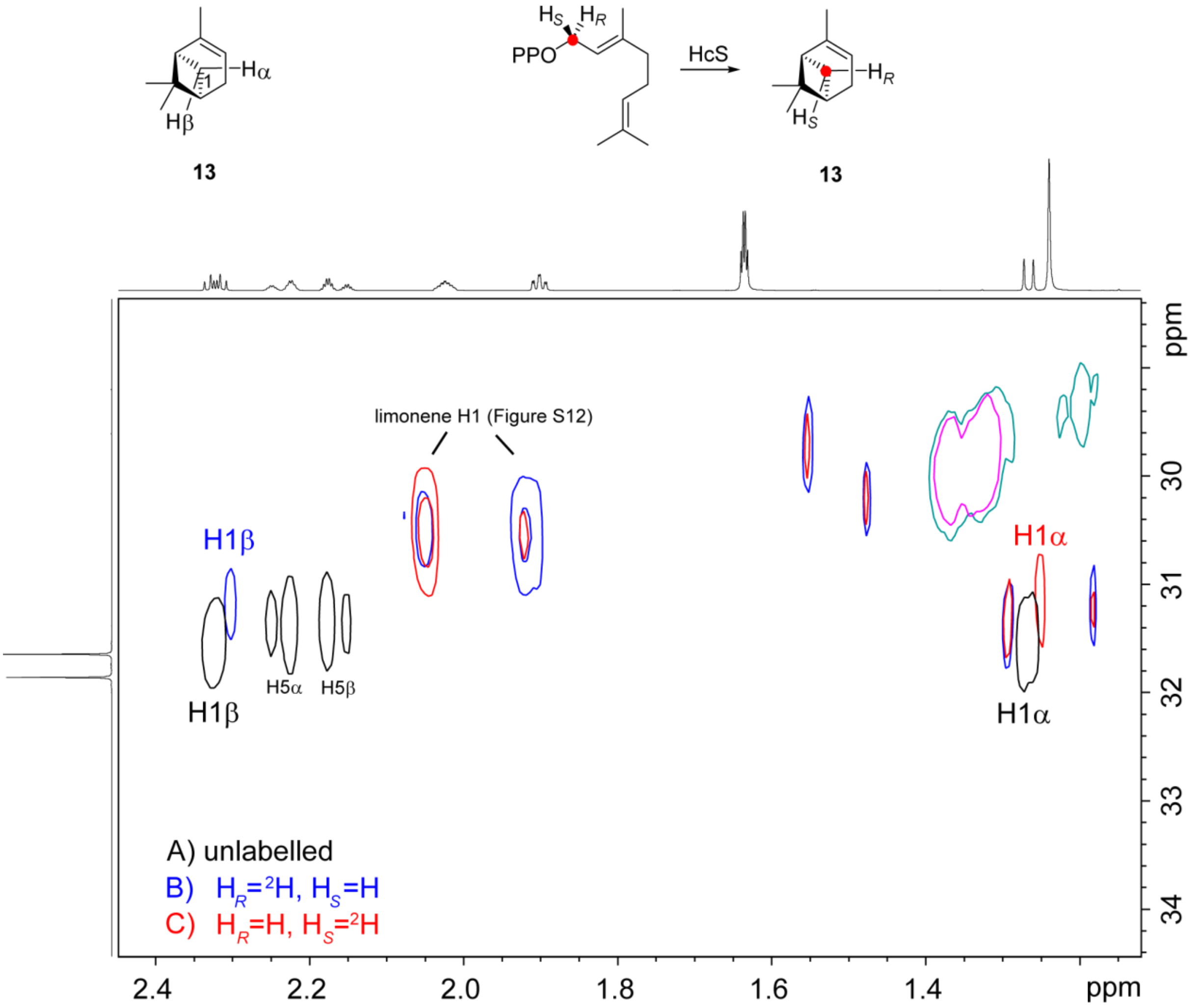

Figure S14: Investigation of the stereochemical course during the HcS catalysed formation of **13**. Overlaid partial HSQC of A) unlabelled **13** (shown in black), B) an incubation of HcS with (1*R*)-(1-^{13}C,1-^{2}H)GPP (shown in blue) and C) an incubation of HcS with (1*S*)-(1-^{13}C,1-^{2}H)GPP (shown in red). The contour representation was in chosen in this case, because **13** is only produced in trace amounts by HcS (compare Figure 1 in main text). The one-dimensional projections shown represent the unlabelled compound. The assignment of the α– and β–position is based on NOE correlations. HcS produces a mixture of enantiomers.

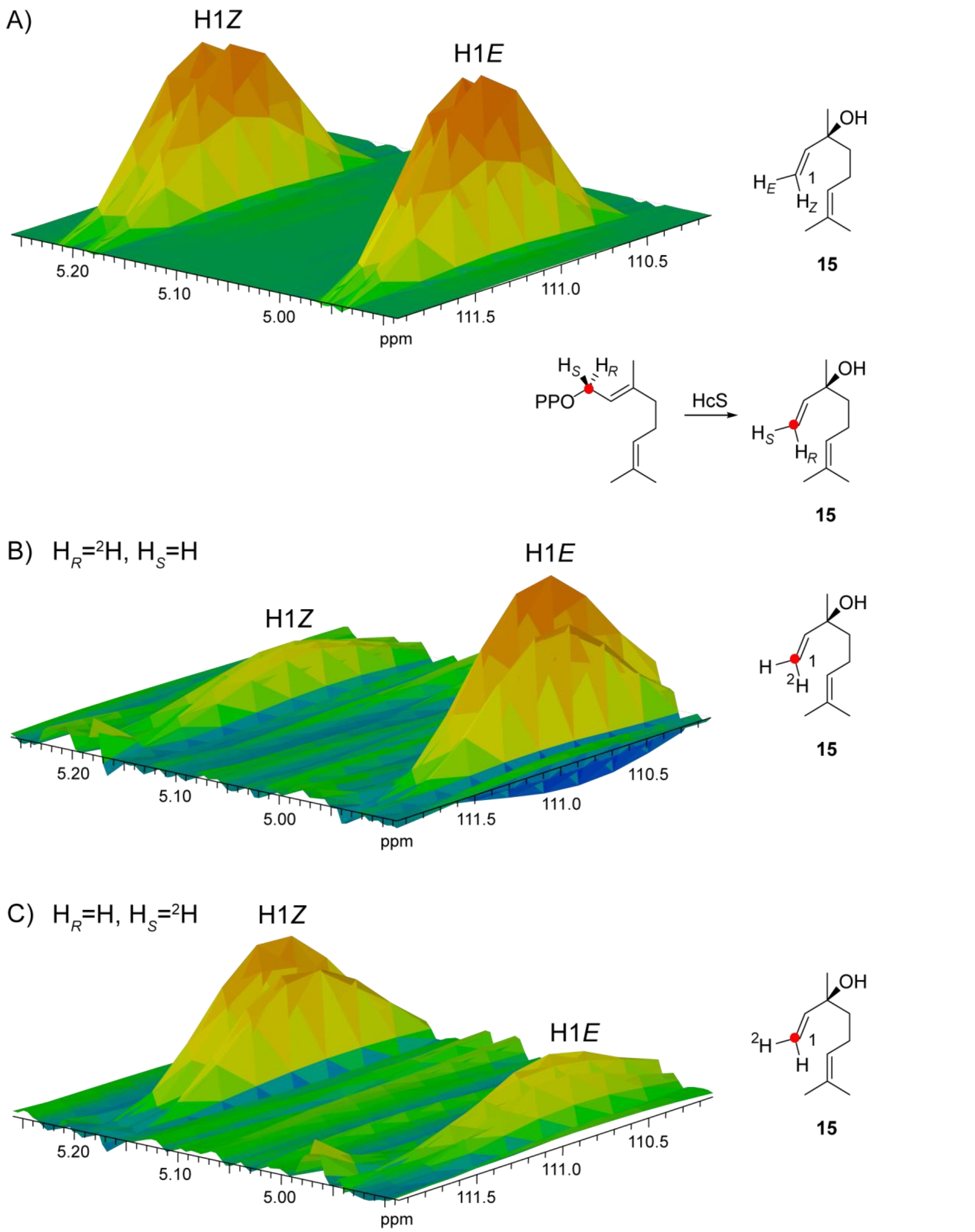

Figure S15: Investigation of the stereochemical course during the formation of **15**. Partial HSQC of A) unlabelled **15**, B) an incubation of HcS with (1*R*)-(1-^{13}C,1-^{2}H)GPP and C) an incubation of HcS with (1*S*)-(1-^{13}C,1-^{2}H)GPP. The assignment of the (*E*)– and (*Z*)–position is based on their different coupling constants. A mixture of enantiomers is observed in the incubation, which is reflected in significantly enlarged minor signals for B) and C). For clarity, only the major enantiomer is shown in the structures. Compound **15** is also known as a non-enzymatic degradation product of GPP.

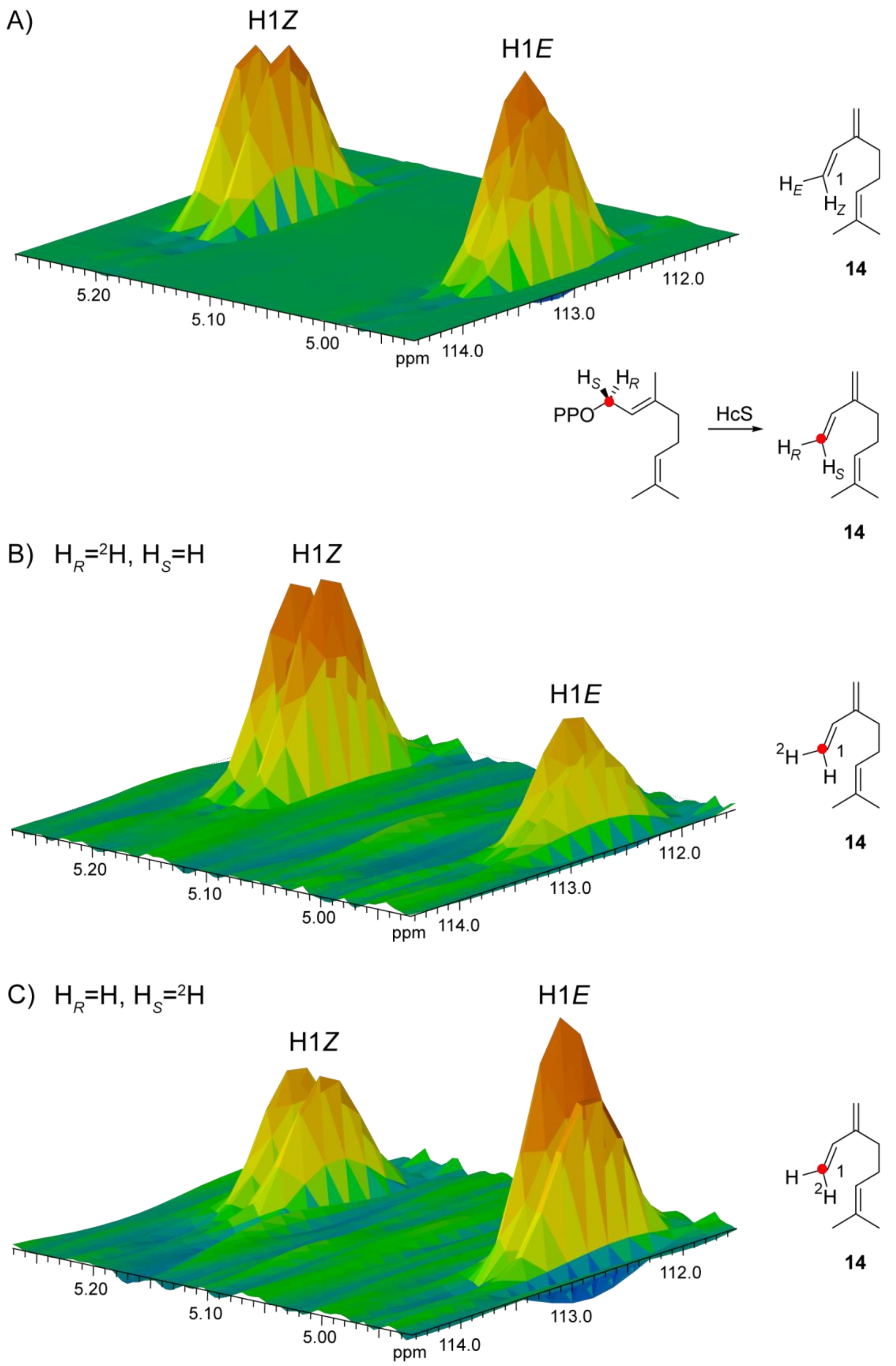

Figure S16: Investigation of the stereochemical course during the formation of **14**. Partial HSQC of A) unlabelled **14**, B) an incubation of HcS with (1*R*)-(1-^{13}C,1-^{2}H)GPP and C) an incubation of HcS with (1*S*)-(1-^{13}C,1-^{2}H)GPP. The assignment of the (*E*)– and (*Z*)–position is based on their different coupling constants. For clarity, only the major diastereomer is shown in the structures. Compound **14** is also known as a non-enzymatic degradation product of GPP.

Table S9: NMR data of cembrene A (**17**) in C_6D_6 recorded at 298 K taken from ref. [17].

C[a]		^{1}H[b]	^{13}C[b]
1	CH_2	2.17 – 2.12 (m, H_β)	33.0
		2.04 – 1.98 (m, H_α)	
2	CH	5.32 – 5.29 (m)	124.6
3	C_q	–	135.0
4	CH_2	2.17 – 2.06 (m, 2H)	39.4
5	CH_2	2.29 – 2.22 (m)	25.4
		2.16 – 2.09 (m)	
6	CH	5.09 – 5.06 (m)	126.5
7	C_q	–	133.5
8	CH_2	2.15 – 2.05 (m, 2H)	39.9
9	CH_2	2.17 – 2.06 (m, 2H)	24.2
10	CH	5.26 – 5.23 (m)	122.3
11	C_q	–	133.9
12	CH_2	2.05 – 2.00 (m)	34.4
		1.96 – 1.90 (m)	
13	CH_2	1.79 (dddd, ^{2}J=13.9, ^{3}J=10.0, 3.9, 3.9, H_β)	28.6
		1.40 (dddd, ^{2}J=13.8, ^{3}J=10.0, 6.7, 3.8, H_α)	
14	CH	2.21 (dddd, ^{3}J=9.9, 9.9, 3.5, 3.5)	46.6
15	C_q	–	149.2
16[c]	CH_3	1.62 (m)	19.4
17[c]	CH_2	4.82 (m)	110.9
18	CH_3	1.59 (s)	18.3
19	CH_3	1.53 (s)	15.4
20	CH_3	1.54 (s)	15.7

[a] Carbon numbering as shown in Figure 2 of main text. [b] Chemical shifts δ in ppm, multiplicity: s = singlet, d = doublet, m = multiplet, coupling constants *J* are given in Hertz. [c] Positions may be interchanged.

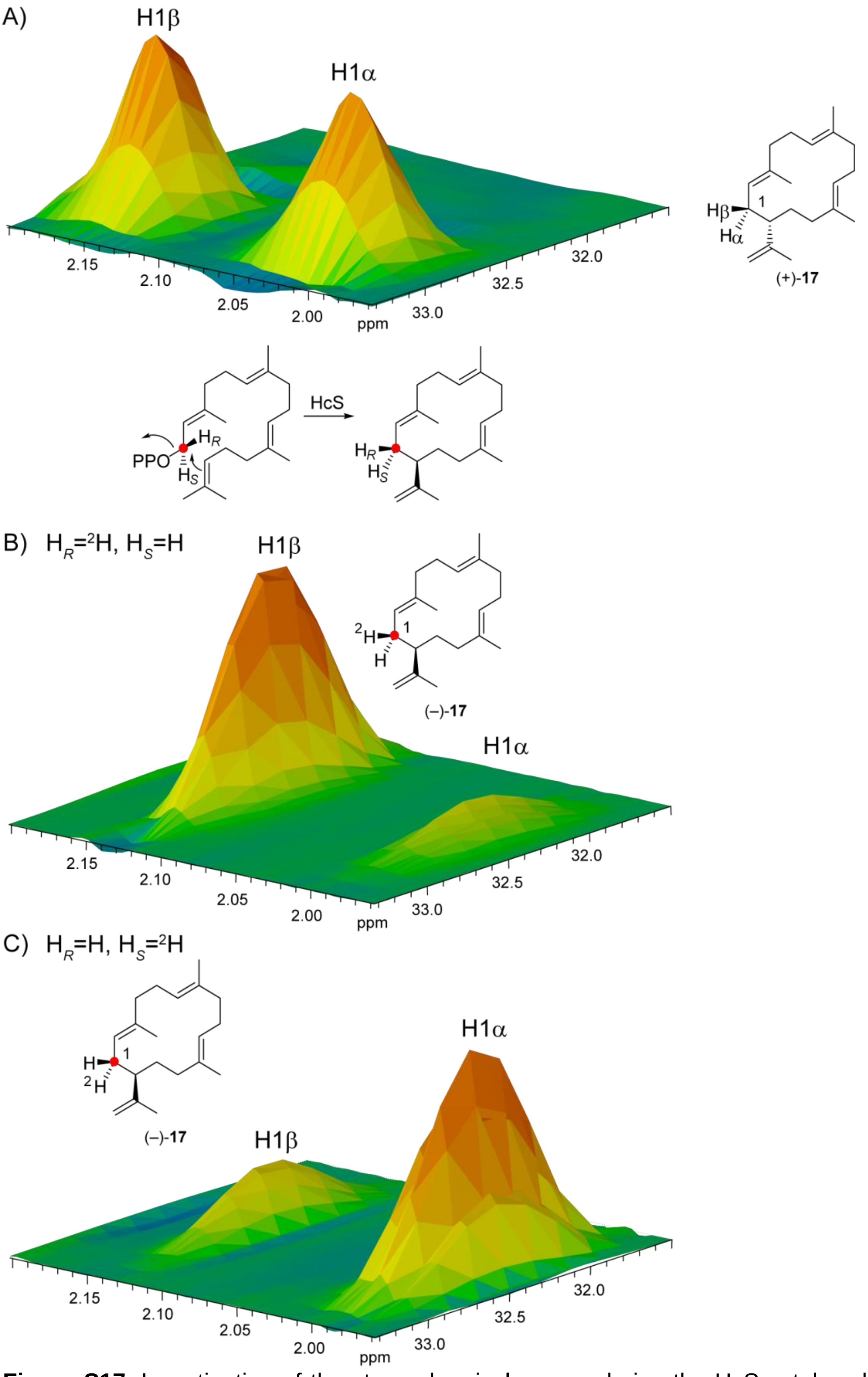

Figure S17: Investigation of the stereochemical course during the HcS catalysed formation of **17**. Partial HSQC of A) unlabelled **17**, B) an incubation of HcS with (1*R*)-(1-^{13}C,1-^{2}H)GGPP and C) an incubation of HcS with (1*S*)-(1-^{13}C,1-^{2}H)GGPP. The assignment of the α– and β–position is based on NOE correlations. HcS produces a mixture of enantiomers, which is reflected in significantly enlarged minor signals for B) and C). For clarity, only the major enantiomer is shown in the structures.

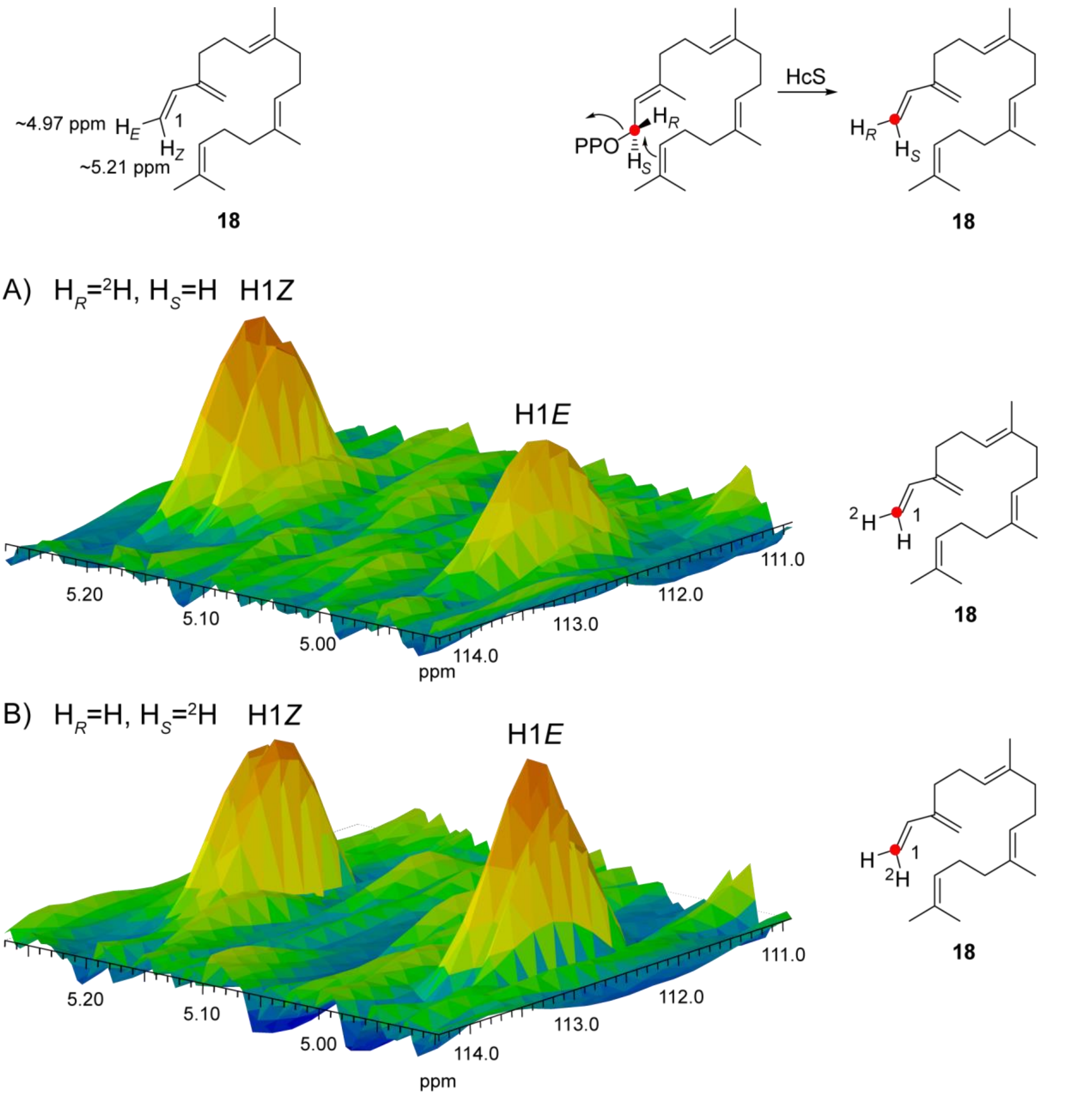

Figure S18: Investigation of the stereochemical course during the formation of **18**. Partial HSQC of A) an incubation of HcS with (1*R*)-(1-^{13}C,1-^{2}H)GGPP and B) an incubation of HcS with (1*S*)-(1-^{13}C,1-^{2}H)GGPP. Because no standard reference material was availibe, the assignment of the (*E*)– and (*Z*)–position is based on the observed chemical shifts for the homolog **14** (cf. Figure S16). For clarity, only the major diastereomer is shown in the structures. Compound **18** is also known as a non-enzymatic degradation product of GGPP.

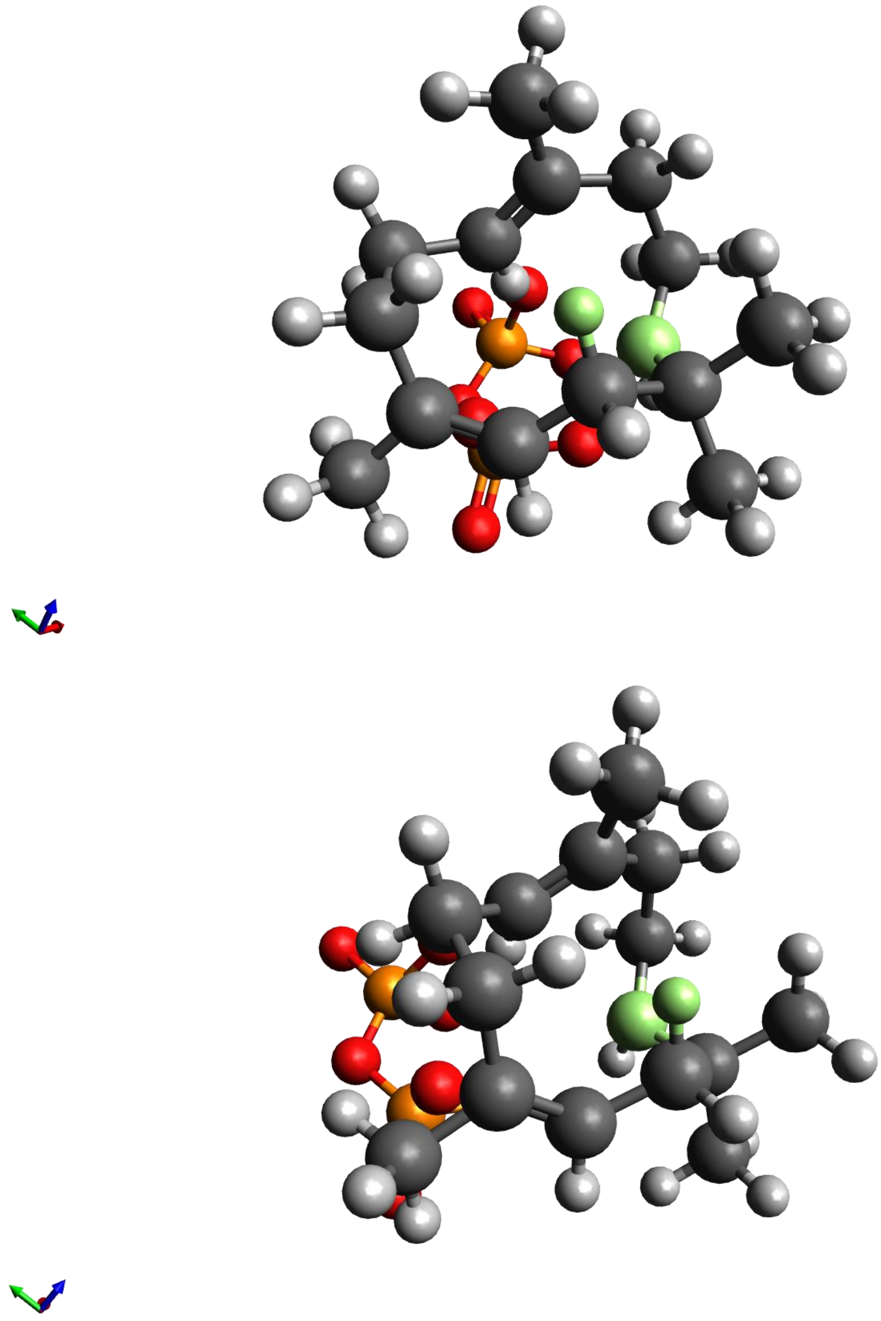

Figure S19: Ball-and-stick model of a proposed conformation of central cation **D** within the active site of HcS together with inorganic diphosphate (OPP^-) shown from two different perspectives. Both C-10 and H_R of C-1 are coloured in light green. This conformation rationalises A) the short distance of H_R and C-10 for a 1,3-hydride shift and the resulting stereochemistry at C-10 after the migration, B) the stereochemical course regarding the methyl groups C-12 and C-13, C) the proximity of C-2 and C-10 for bond formation, which gives rise to the side product 9-*epi*-β-caryophyllene (**7**) with correct stereochemistry at the bridgeheads and at C-1, D) the orientation of the C-6, C-7 double bond for follow up ring closure of the six-membered ring, E) the conformational imprint of (*R*)-NPP (proximity of C-3 to OPP^-) with the correct stereochemistry at C-1 for the 1,11 ring closure and F) the thereby resulting position of OPP^-, covering almost the complete backside of the cyclising cation explaining the low product selectivity because many backwards pointing hydrogens are prone to deprotonation by OPP^-. The structure is not optimised and does not aim for an exact representation, but is discussed for a better understanding the reactions catalysed by HcS. The images were generated using Avogadro modelling software version 1.1.1. Colouring: C (dark grey), H (light grey), O (red), P (orange).

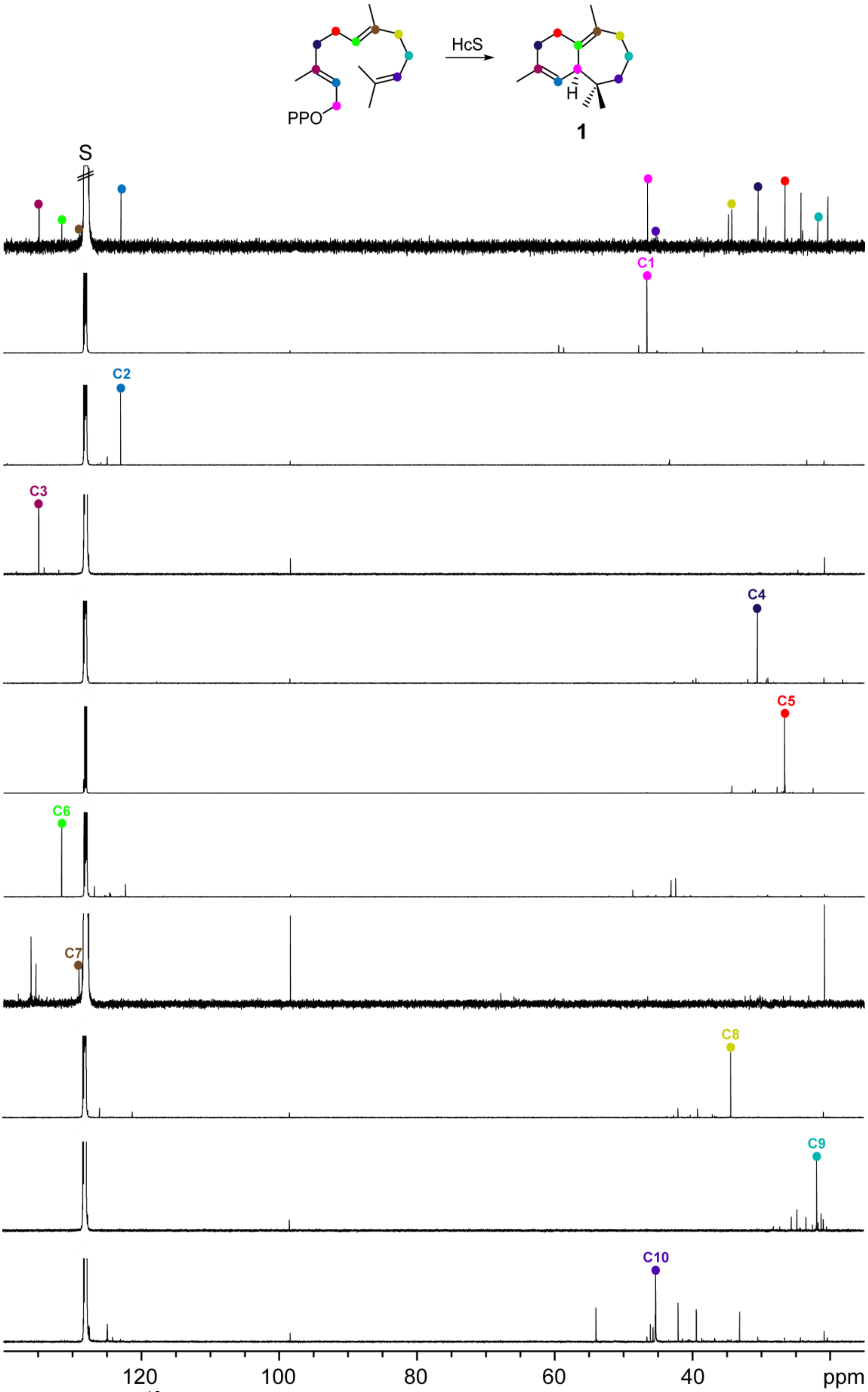

Figure S20: ^{13}C-NMR spectra of extracts obtained from incubations of all fifteen singly labelled FPP isotopomers (enzymatically or synthetically prepared) with HcS. Peaks assigned to the main product **1** by comparison with the unlabelled spectra shown on top are highlighted by coloured dots.

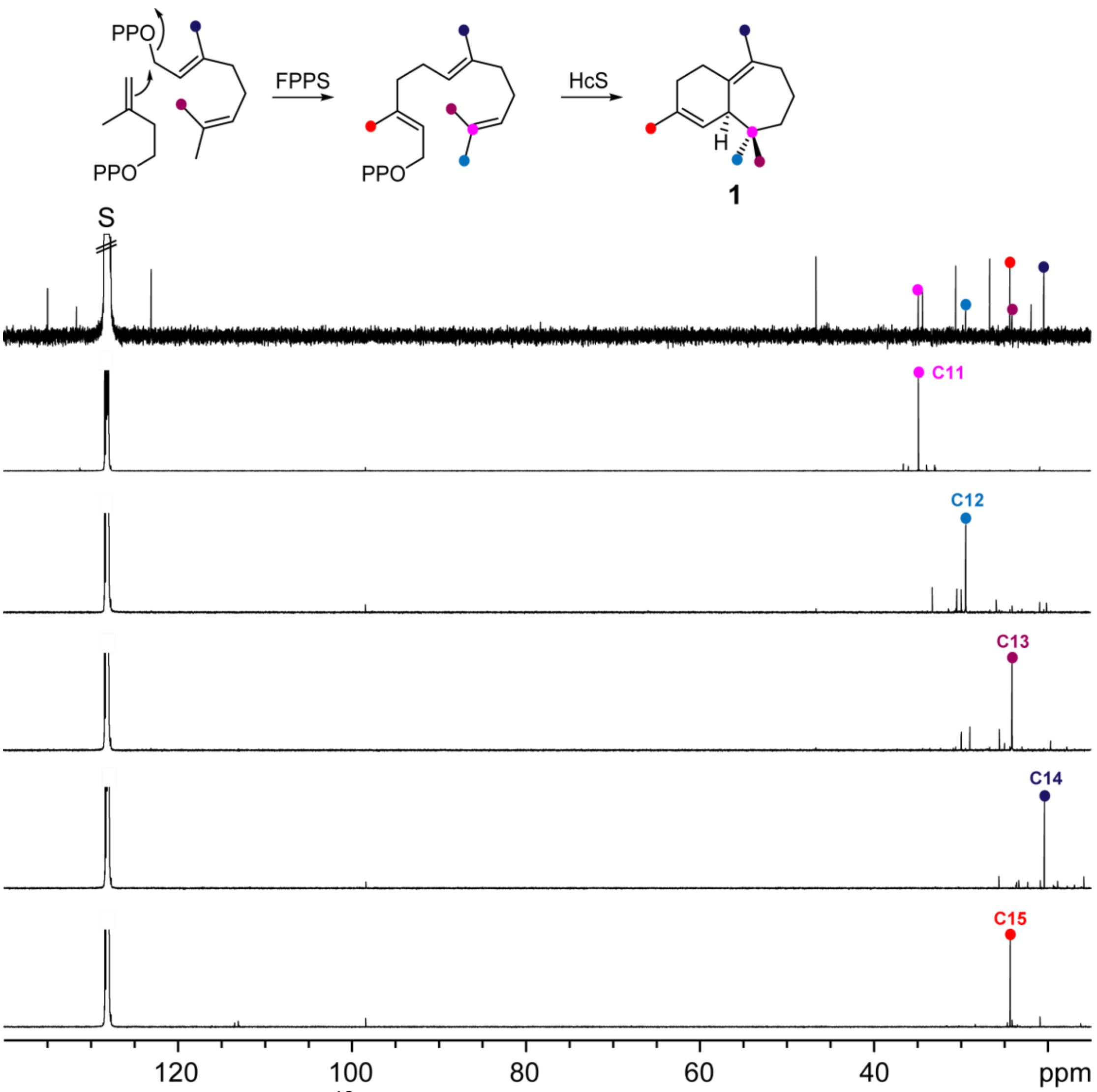

Figure S20 (continued): ^{13}C-NMR spectra of extracts obtained from incubations of all fifteen singly labelled FPP isotopomers (enzymatically or synthetically prepared) with HcS. Peaks assigned to the main product **1** by comparison with the unlabelled spectra shown on top are highlighted by coloured dots.

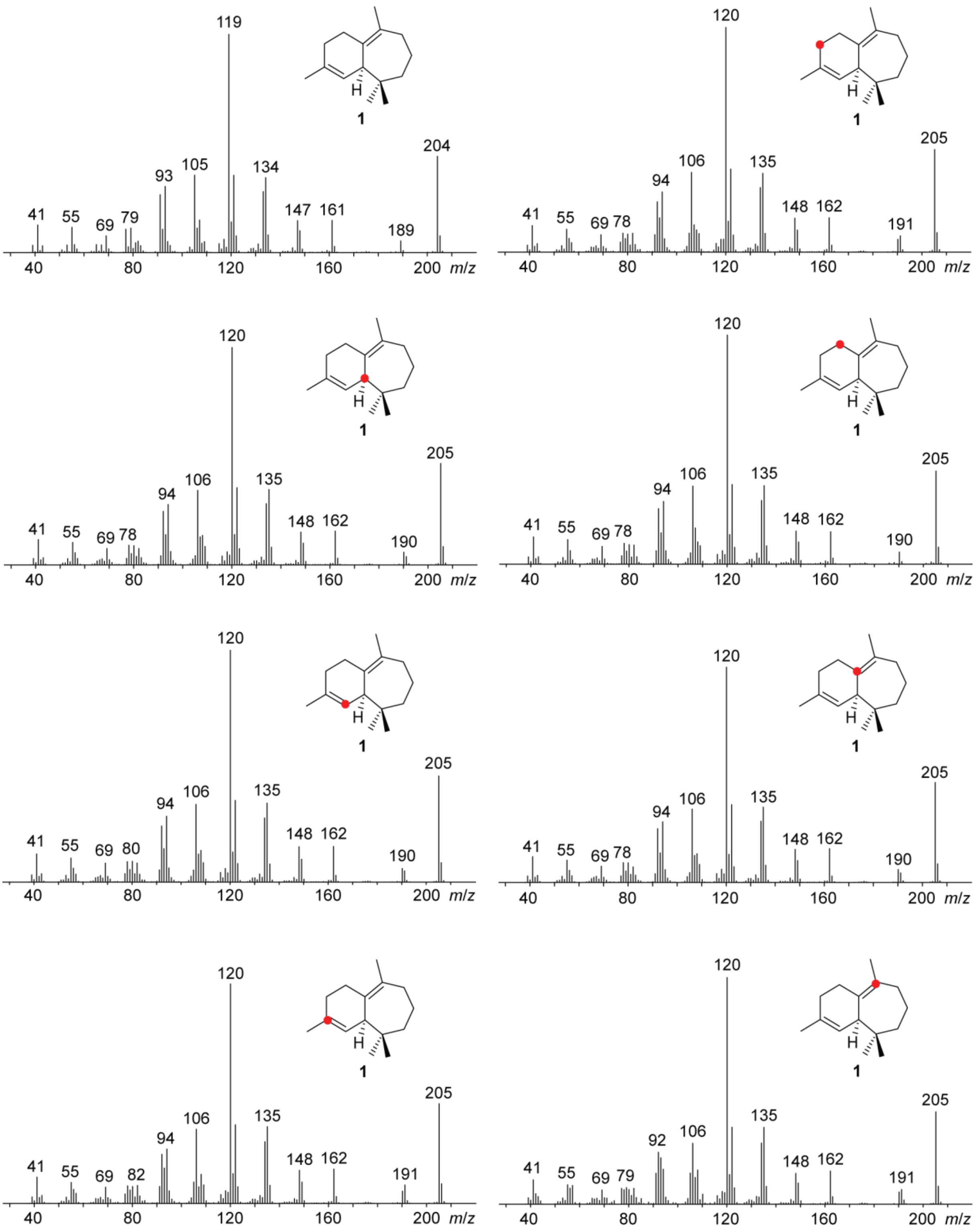

Figure S21: Position specific mass shift analysis [14,20] for **1**. EIMS spectra of all fifteen singly ^{13}C-labelled, enzymatically prepared isotopomers of **1** together with the unlabelled case as obtained by GC–MS analysis. The position of the ^{13}C-labelled carbon atom is shown by a red dot for each mass spectrum.

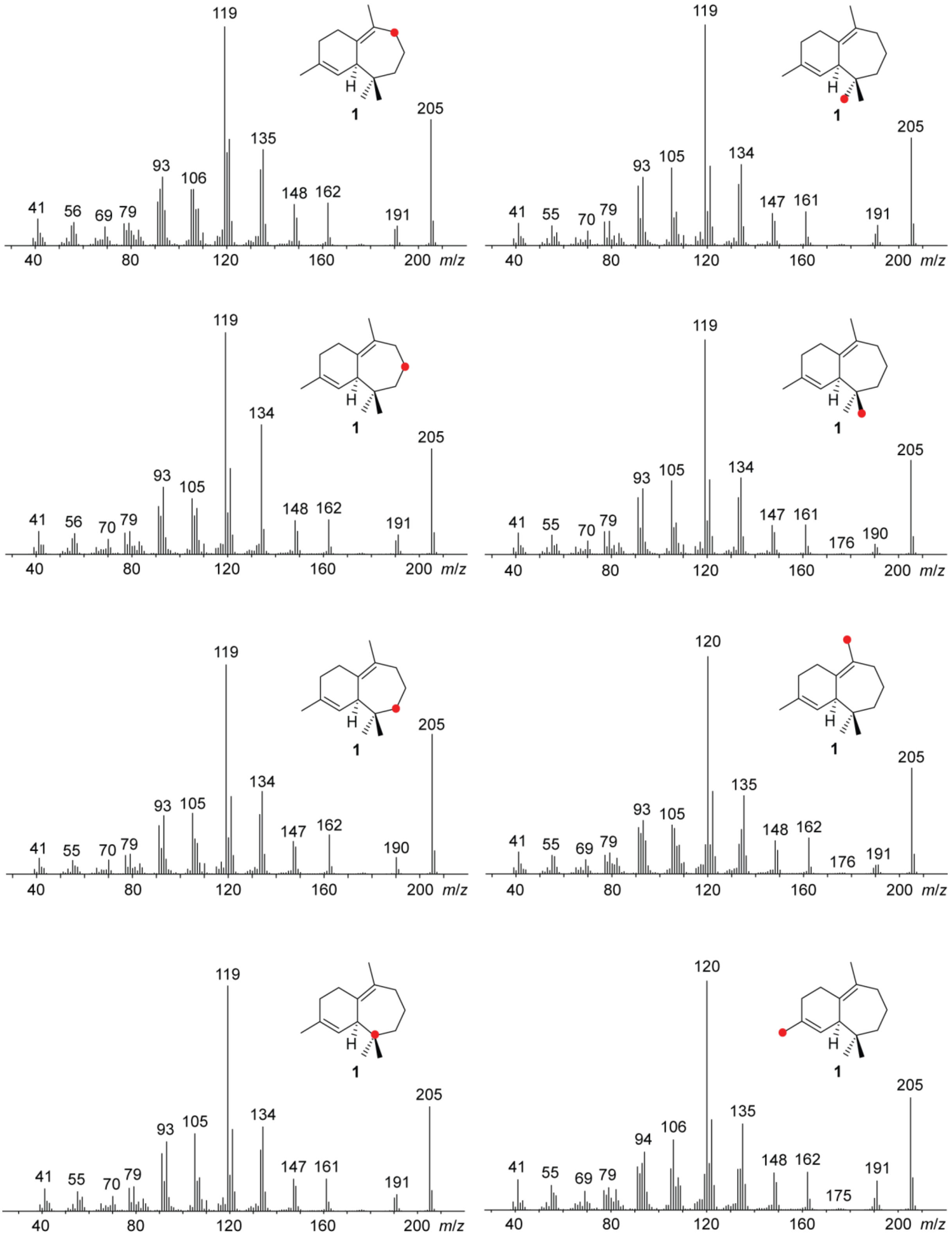

Figure S21 (continued): Position specific mass shift analysis for **1**. EIMS spectra of all fifteen singly ^{13}C-labelled, enzymatically prepared isotopomers of **1** together with the unlabelled case as obtained by GC–MS analysis. The position of the ^{13}C-labelled carbon atom is shown by a red dot for each mass spectrum.

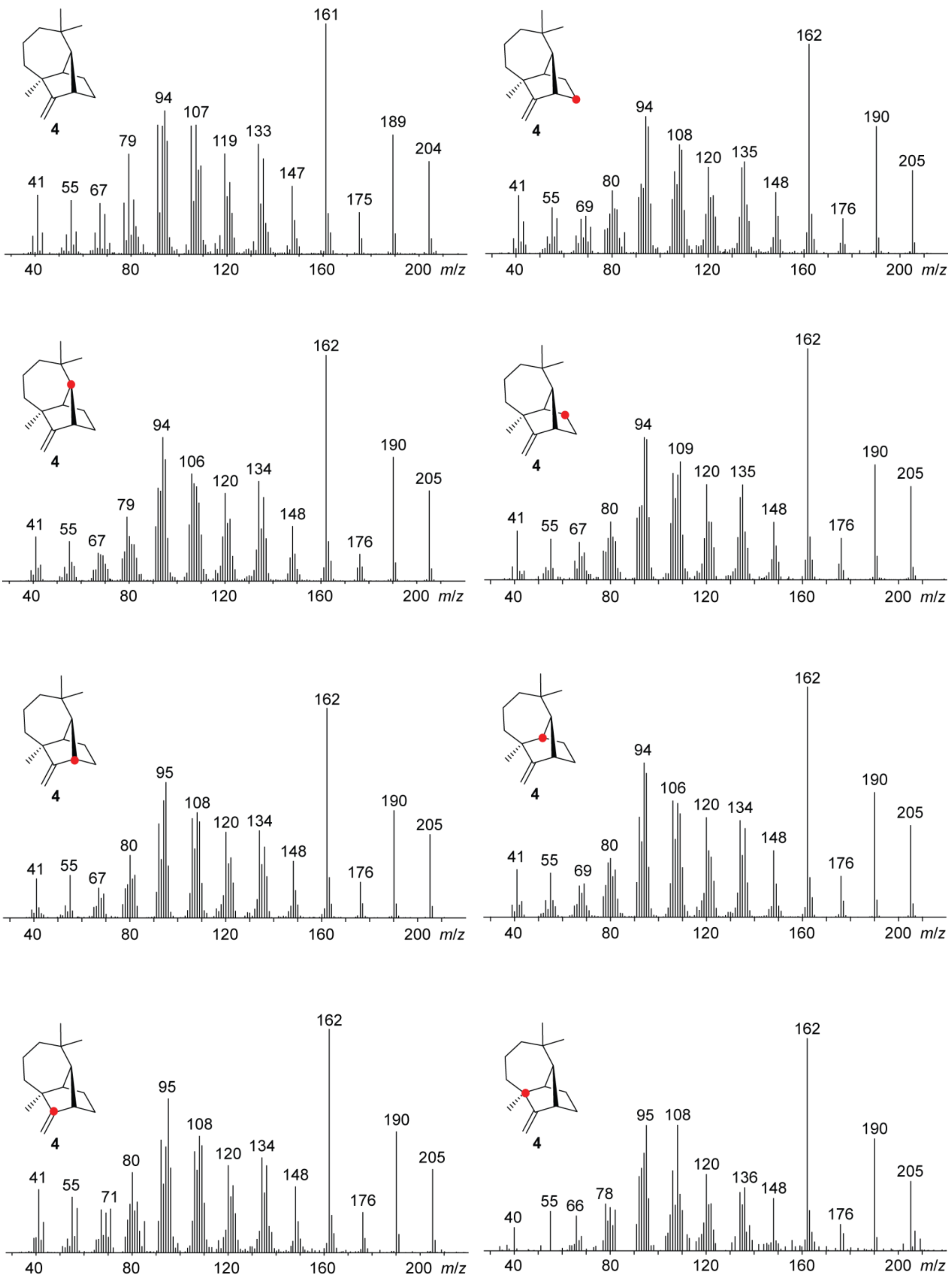

Figure S22: Position specific mass shift analysis for **4**. EIMS spectra of all fifteen singly ^{13}C-labelled, enzymatically prepared isotopomers of **4** together with the unlabelled case as obtained by GC–MS analysis. The position of the ^{13}C-labelled carbon atom is shown by a red dot for each mass spectrum.

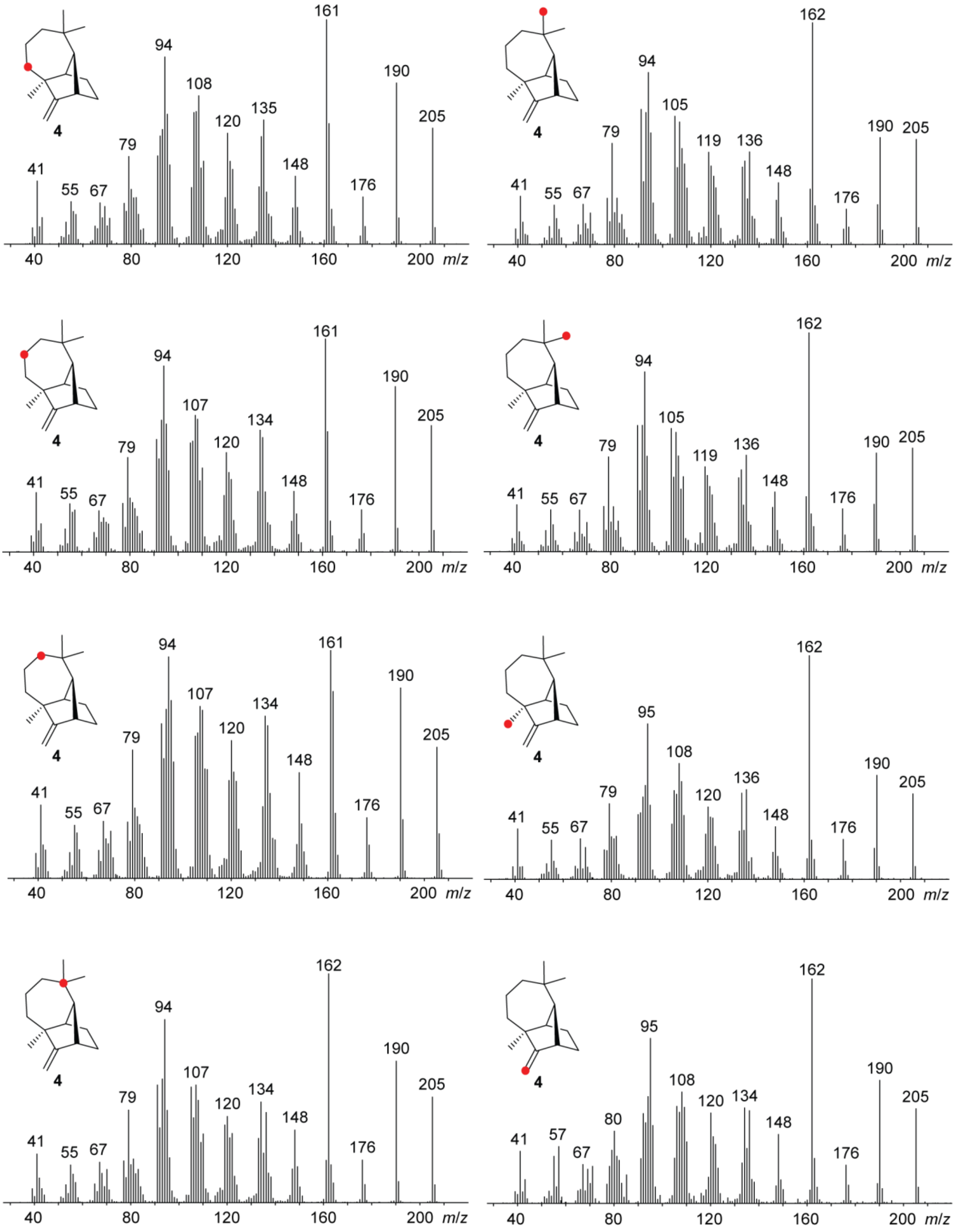

Figure S22 (continued): Position specific mass shift analysis for **4**. EIMS spectra of all fifteen singly ^{13}C-labelled, enzymatically prepared isotopomers of **4** together with the unlabelled case as obtained by GC–MS analysis. The position of the ^{13}C-labelled carbon atom is shown by a red dot for each mass spectrum.

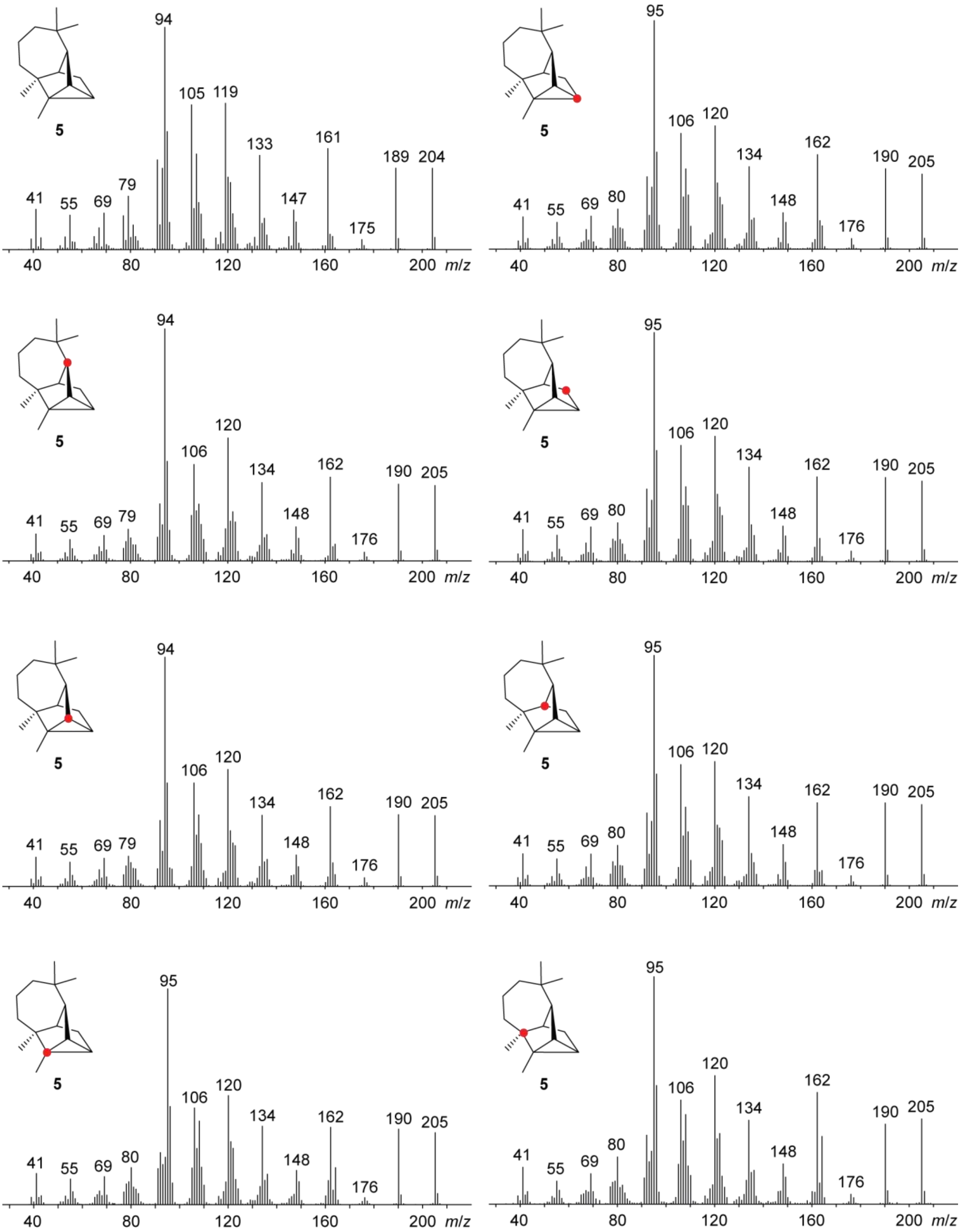

Figure S23: Position specific mass shift analysis for **5**. EIMS spectra of all fifteen singly ^{13}C-labelled, enzymatically prepared isotopomers of **5** together with the unlabelled case as obtained by GC–MS analysis. The position of the ^{13}C-labelled carbon atom is shown by a red dot for each mass spectrum.

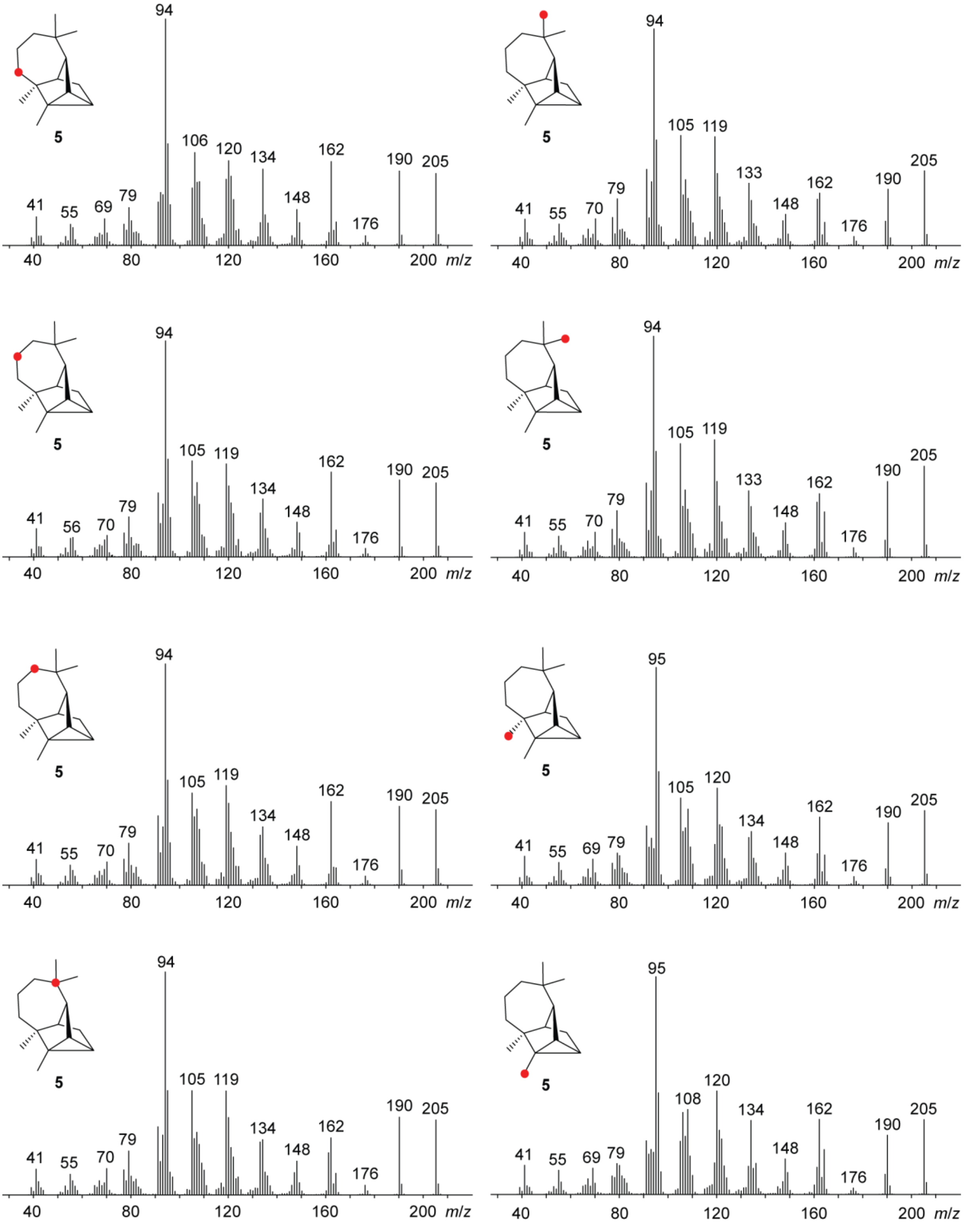

Figure S23 (continued): Position specific mass shift analysis for **5**. EIMS spectra of all fifteen singly ^{13}C-labelled, enzymatically prepared isotopomers of **5** together with the unlabelled case as obtained by GC–MS analysis. The position of the ^{13}C-labelled carbon atom is shown by a red dot for each mass spectrum.

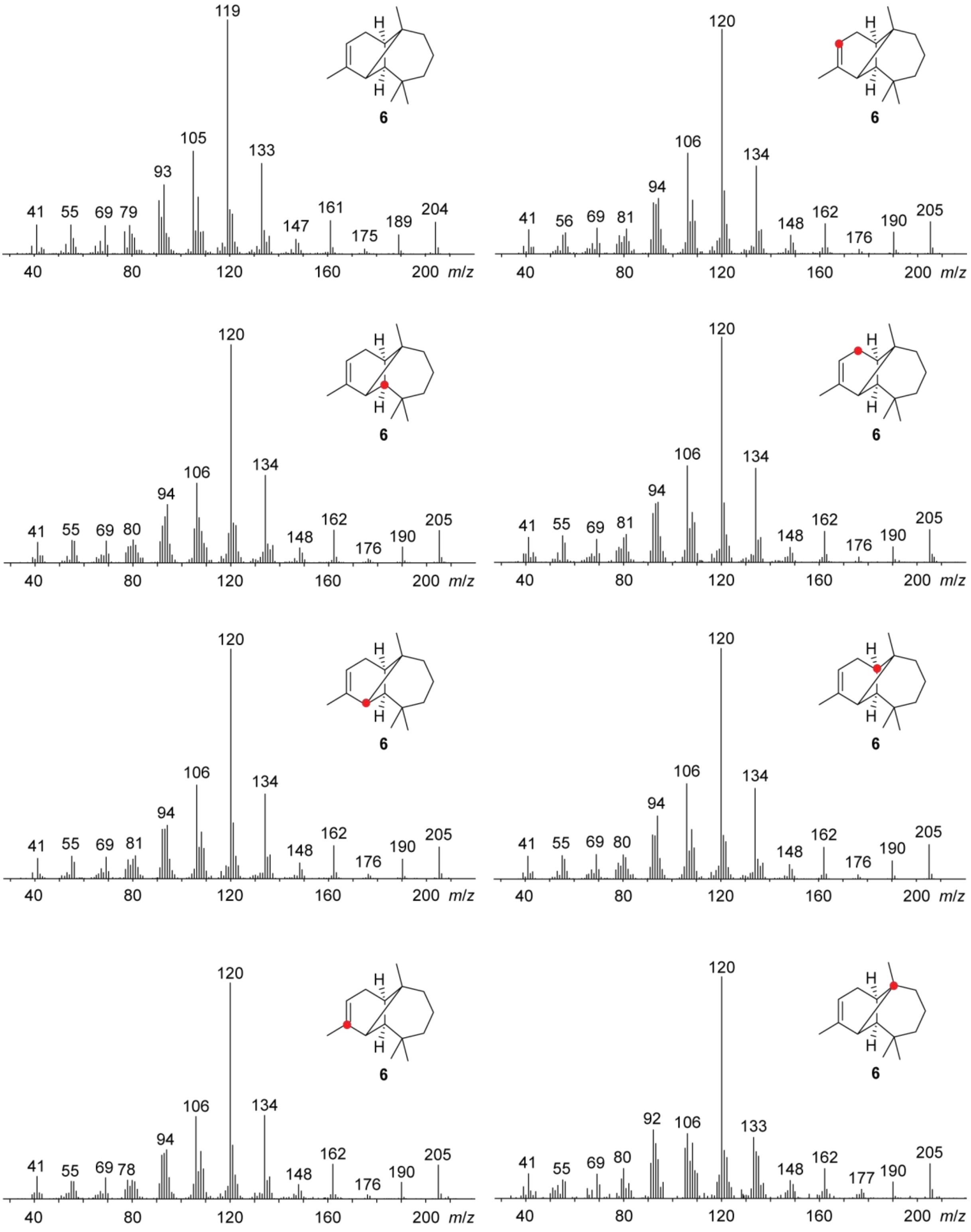

Figure S24: Position specific mass shift analysis for **6**. EIMS spectra of all fifteen singly ^{13}C-labelled, enzymatically prepared isotopomers of **6** together with the unlabelled case as obtained by GC–MS analysis. The position of the ^{13}C-labelled carbon atom is shown by a red dot for each mass spectrum.

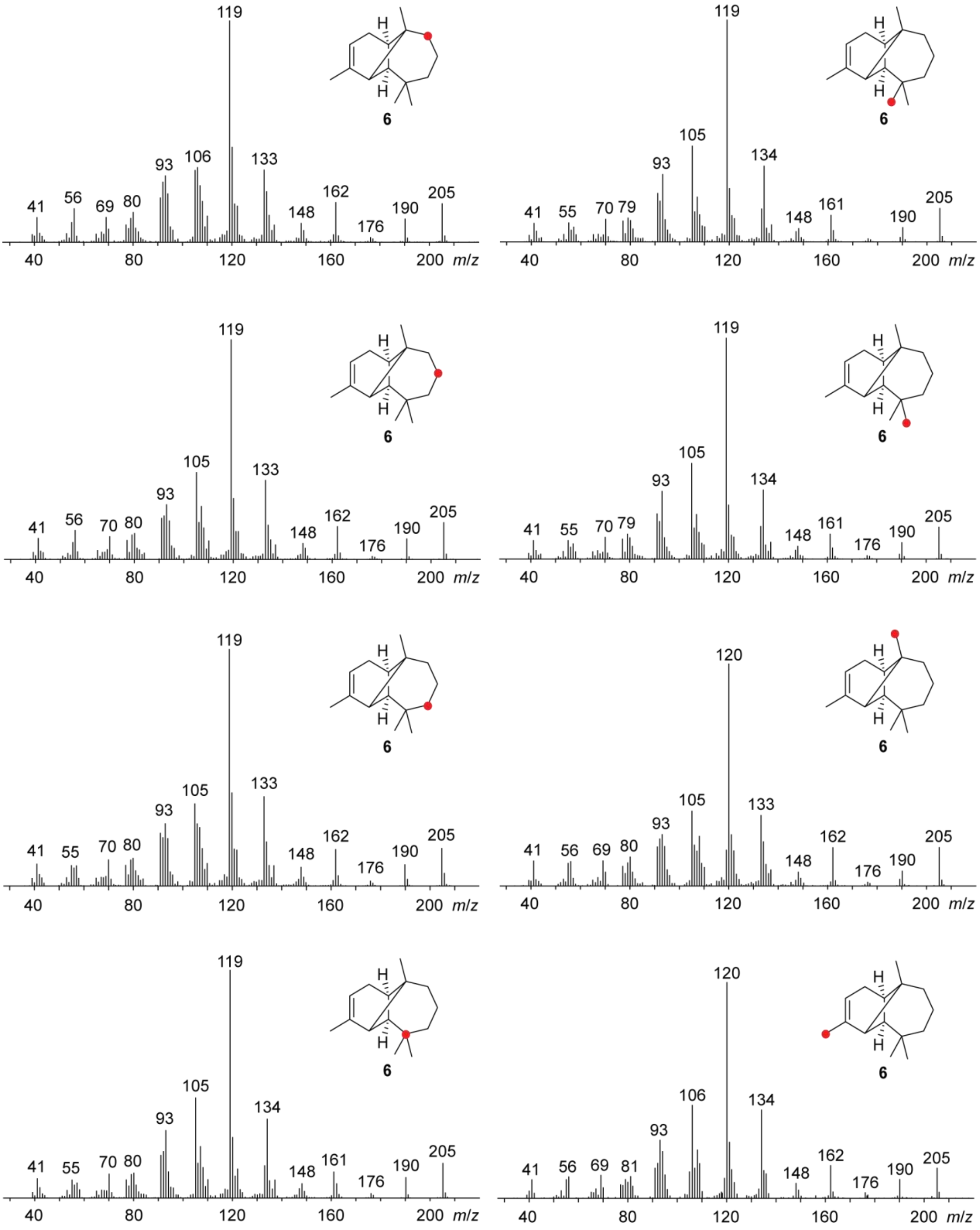

Figure S24 (continued): Position specific mass shift analysis for **6**. EIMS spectra of all fifteen singly ^{13}C-labelled, enzymatically prepared isotopomers of **6** together with the unlabelled case as obtained by GC–MS analysis. The position of the ^{13}C-labelled carbon atom is shown by a red dot for each mass spectrum.

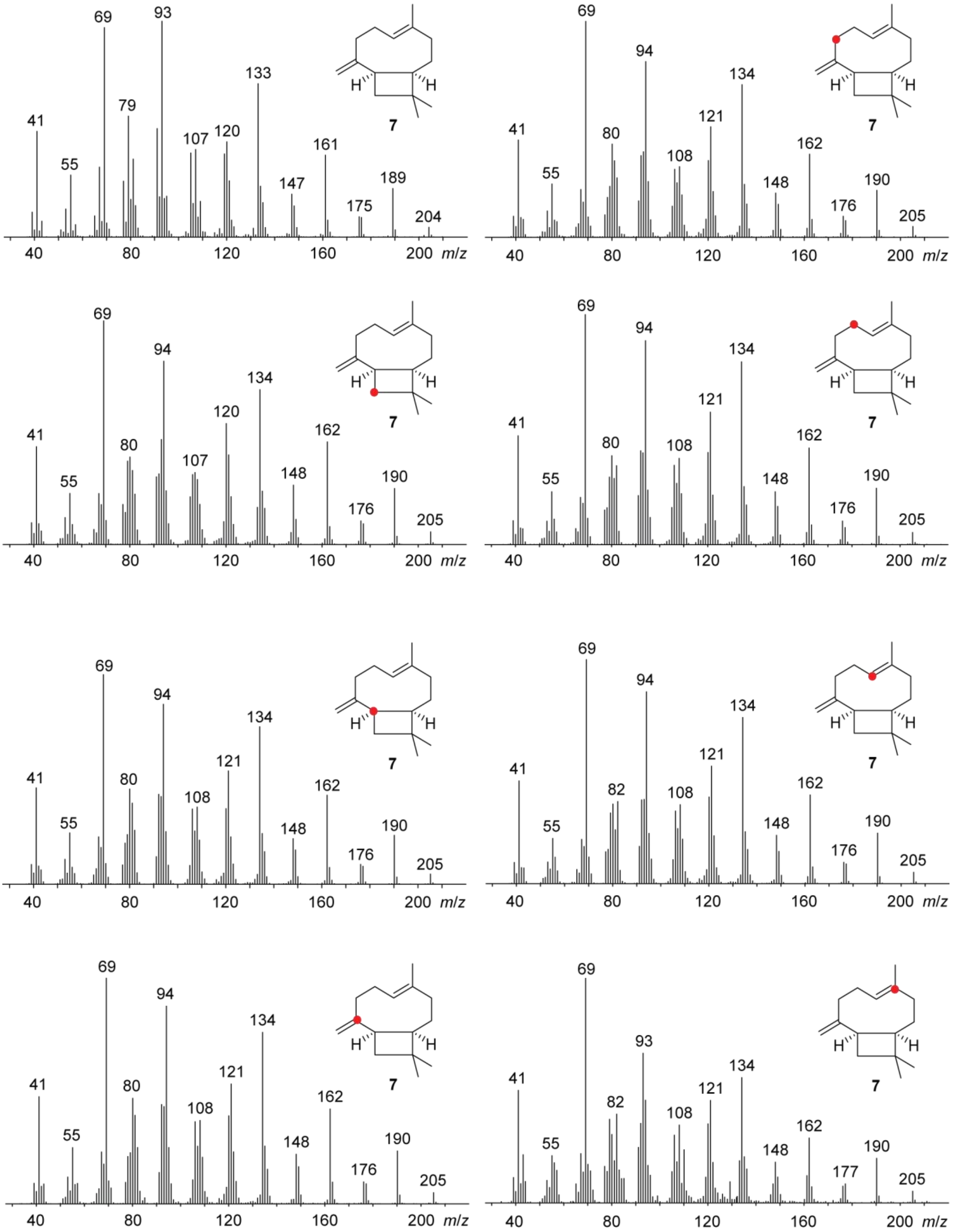

Figure S25: Position specific mass shift analysis for **7**. EIMS spectra of all fifteen singly ^{13}C-labelled, enzymatically prepared isotopomers of **7** together with the unlabelled case as obtained by GC–MS analysis. The position of the ^{13}C-labelled carbon atom is shown by a red dot for each mass spectrum.

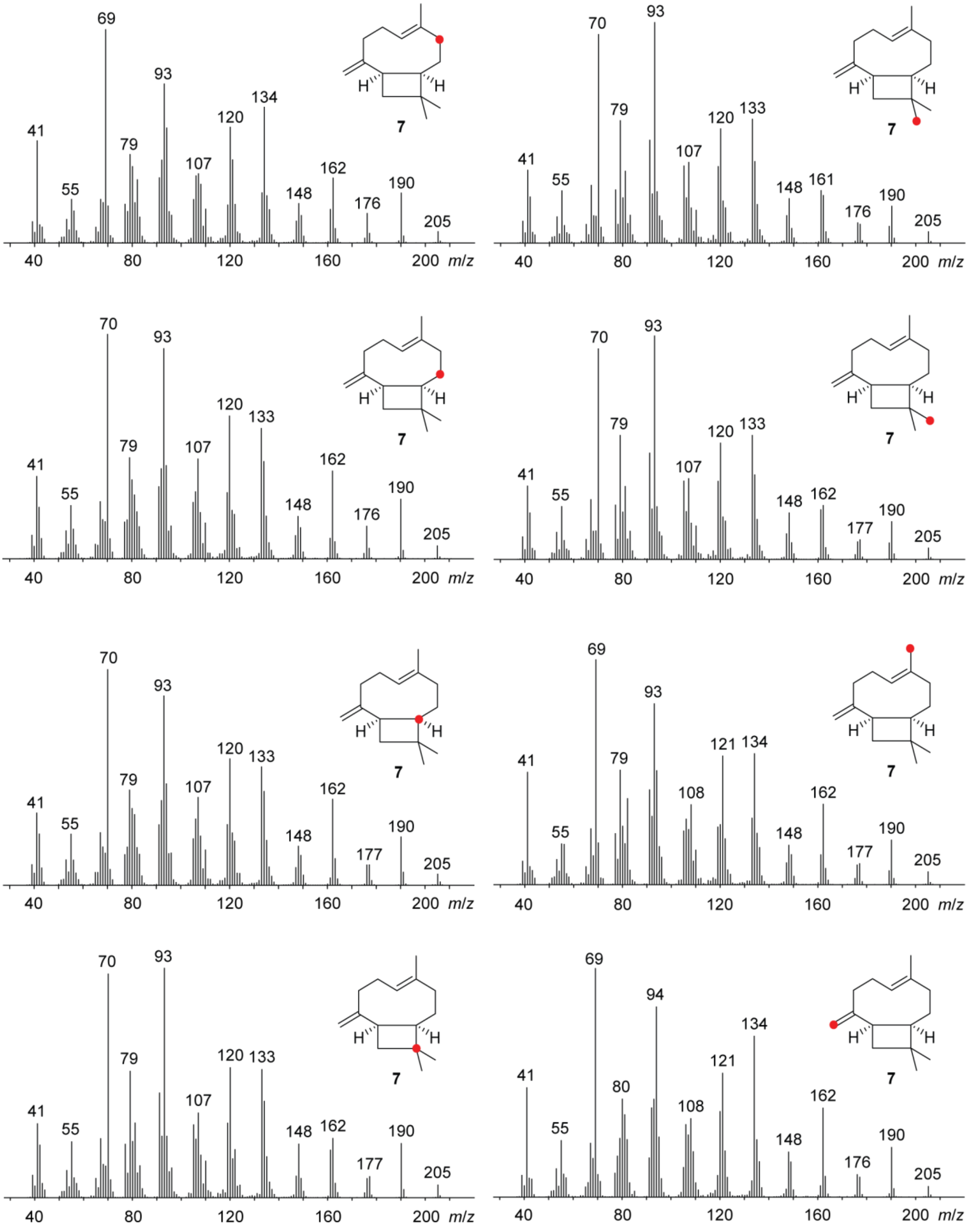

Figure S25 (continued): Position specific mass shift analysis for **7**. EIMS spectra of all fifteen singly ^{13}C-labelled, enzymatically prepared isotopomers of **7** together with the unlabelled case as obtained by GC–MS analysis. The position of the ^{13}C-labelled carbon atom is shown by a red dot for each mass spectrum.

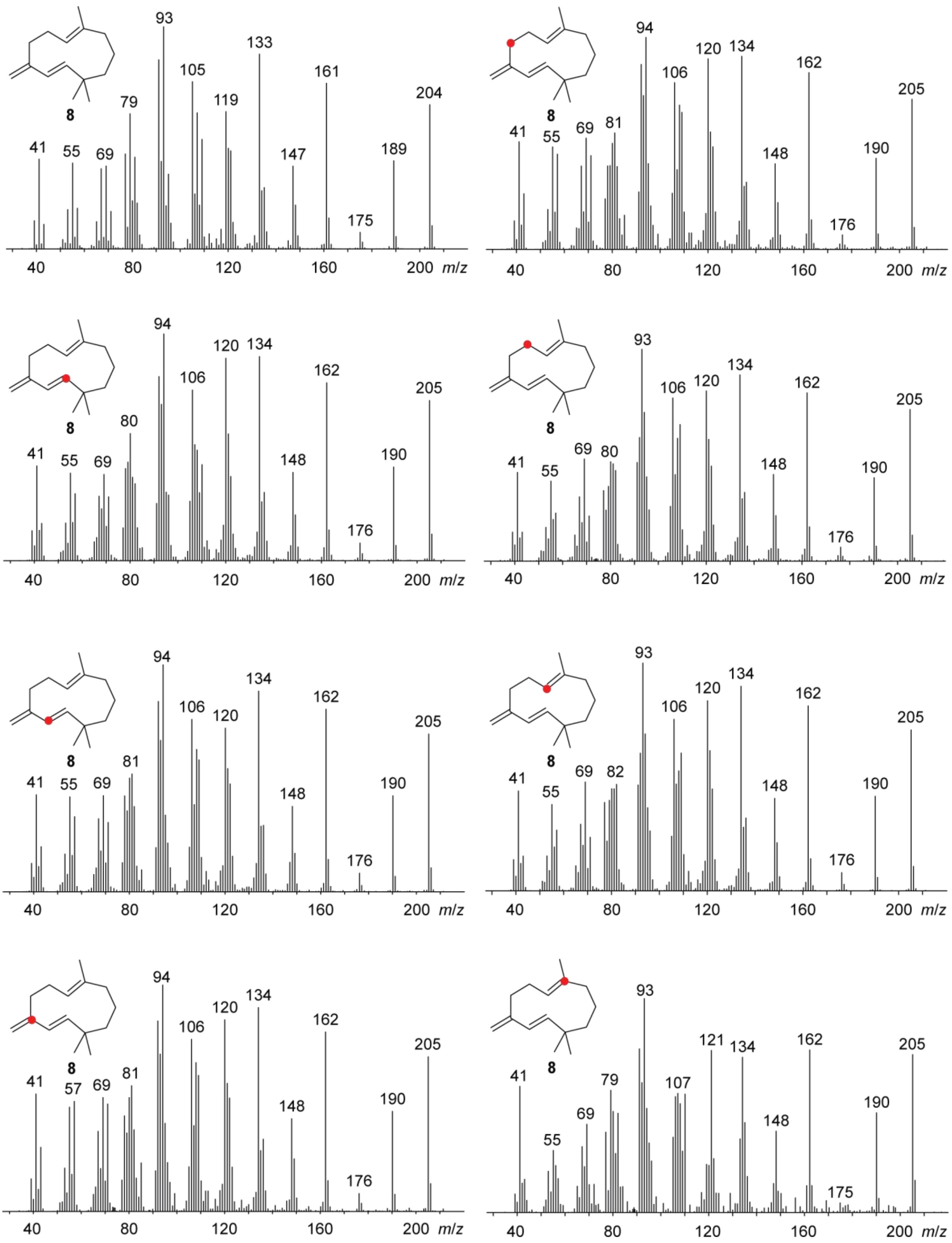

Figure S26: Position specific mass shift analysis for **8**. EIMS spectra of all fifteen singly ^{13}C-labelled, enzymatically prepared isotopomers of **8** together with the unlabelled case as obtained by GC–MS analysis. The position of the ^{13}C-labelled carbon atom is shown by a red dot for each mass spectrum.

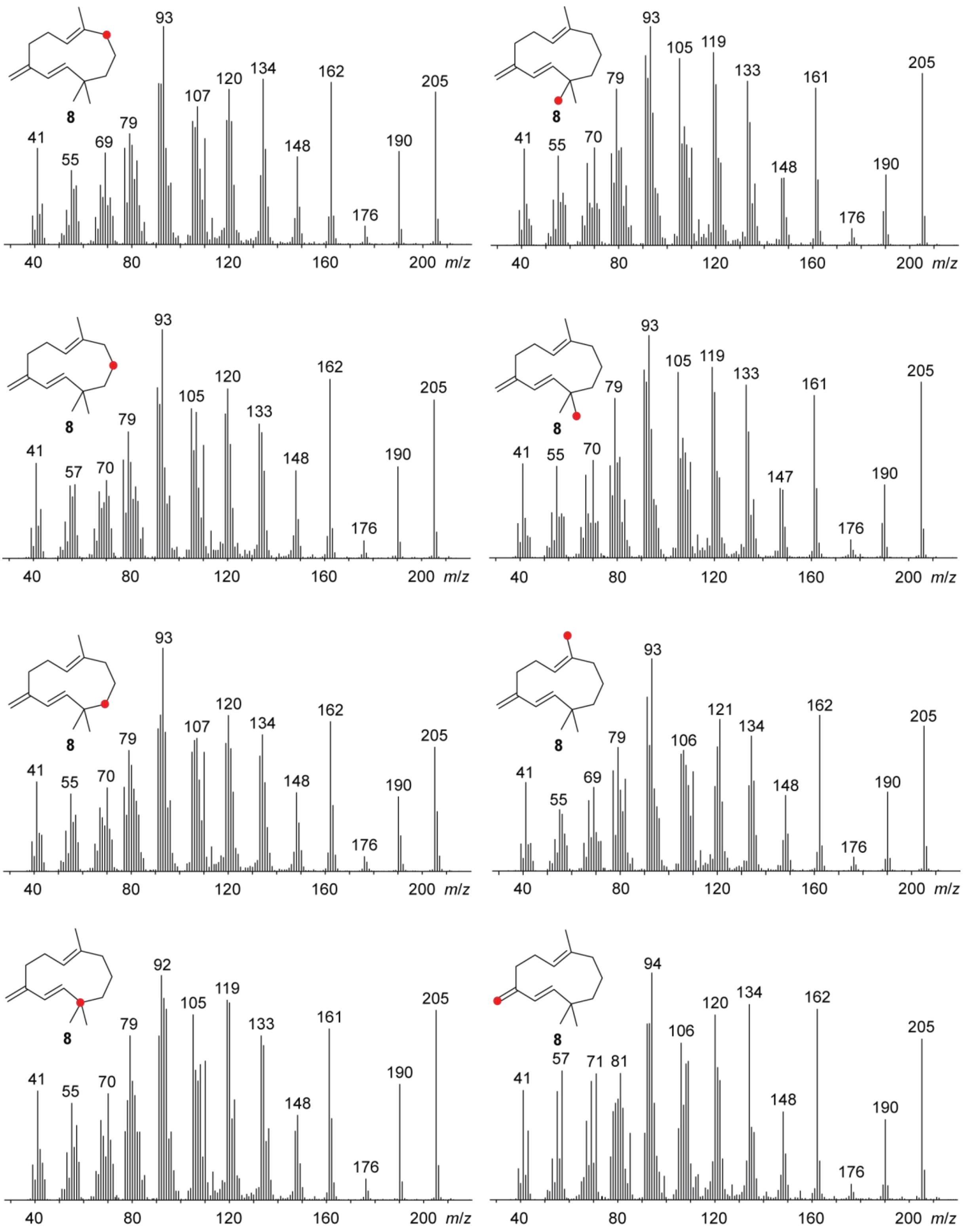

Figure S26 (continued): Position specific mass shift analysis for **8**. EIMS spectra of all fifteen singly ^{13}C-labelled, enzymatically prepared isotopomers of **8** together with the unlabelled case as obtained by GC–MS analysis. The position of the ^{13}C-labelled carbon atom is shown by a red dot for each mass spectrum.

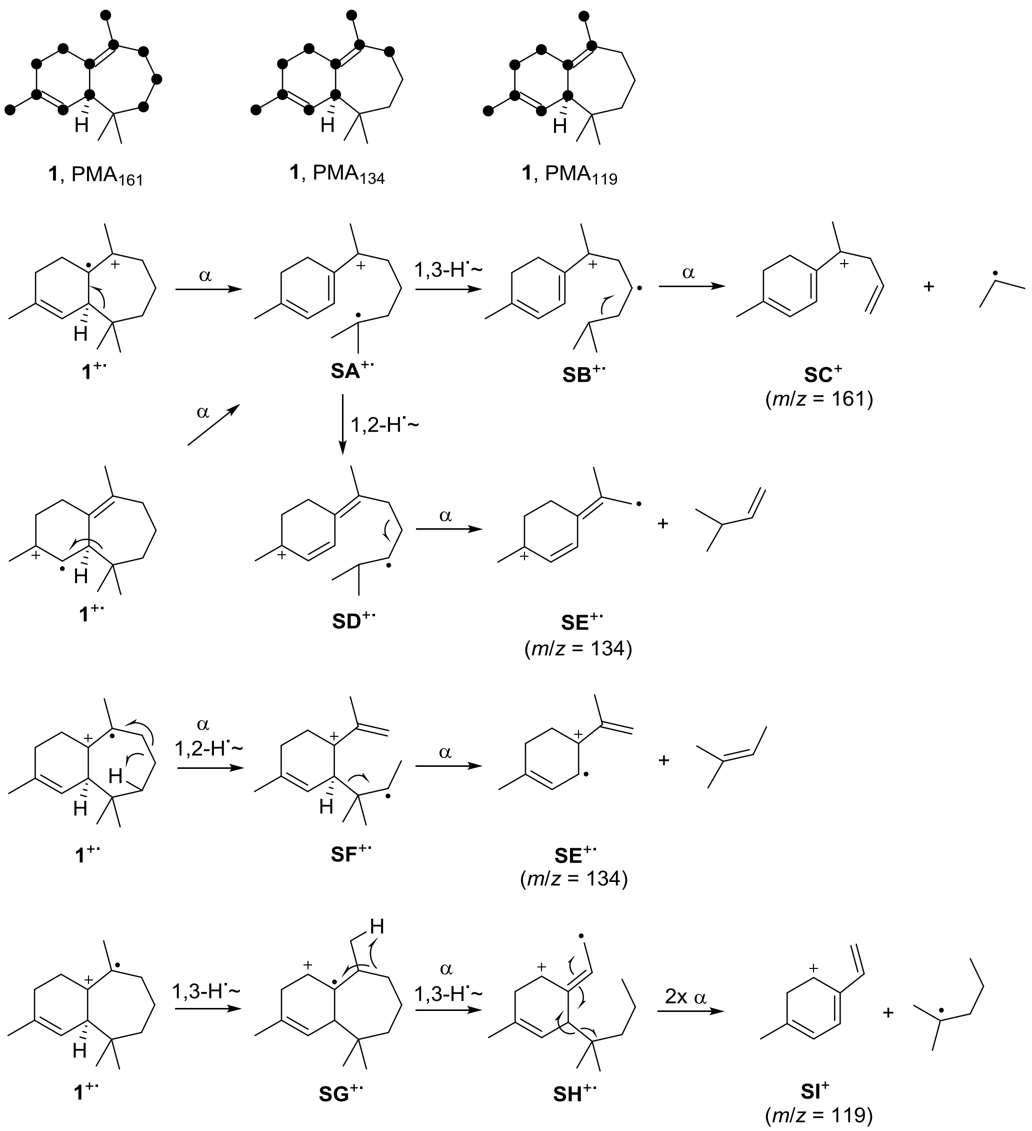

Scheme S1: Proposed EIMS fragmentation mechanisms for the fragments m/z = 161, m/z = 134 and m/z = 119 of **1**. α: α-cleavage, $H^{\bullet}$~: hydrogen rearrangement. Black dots represent carbon atoms that are part of the fragment (Figure S21).

Analysis of PMA_{161} shows a specific loss of C-11, C-12 and C-13. The ionisation of any of the two double bonds can lead to a cleavage of the C-1,C-11 bond, giving $\mathbf{SA}^{+\bullet}$, which after hydrogen rearrangement to $\mathbf{SB}^{+\bullet}$ gives rise to a loss of a propyl radical forming cation $\mathbf{SC}^{+}$ with m/z = 161. Radical cation $\mathbf{SA}^{+\bullet}$ may also rearrange to $\mathbf{SD}^{+\bullet}$, which allows a loss of 3-methylbut-1-ene yielding conjugated radical cation $\mathbf{SE}^{+\bullet}$ with m/z = 134 explaining PMA_{134}. The same fragment can also be obtained starting by a radical induced cleavage of the C-8, C-9 bond followed by hydrogen rearrangement to give $\mathbf{SF}^{+\bullet}$, which loses 2-methylbut-2-ene. Finally, PMA_{119} consists of one methylene group less and indicates a radical loss. A plausible mechanism starts with hydrogen rearrangement of $\mathbf{1}^{+\bullet}$ to $\mathbf{SG}^{+\bullet}$, followed by cleavage of the C-7,C-8 bond which is accompanied by the abstraction of a hydrogen radical from the neighbouring methyl group to $\mathbf{SH}^{+\bullet}$. Two α-fragmentations lead to the loss of a 2-methylpentyl radical and furnishes the conjugated cation $\mathbf{SI}^{+}$ with m/z = 119.

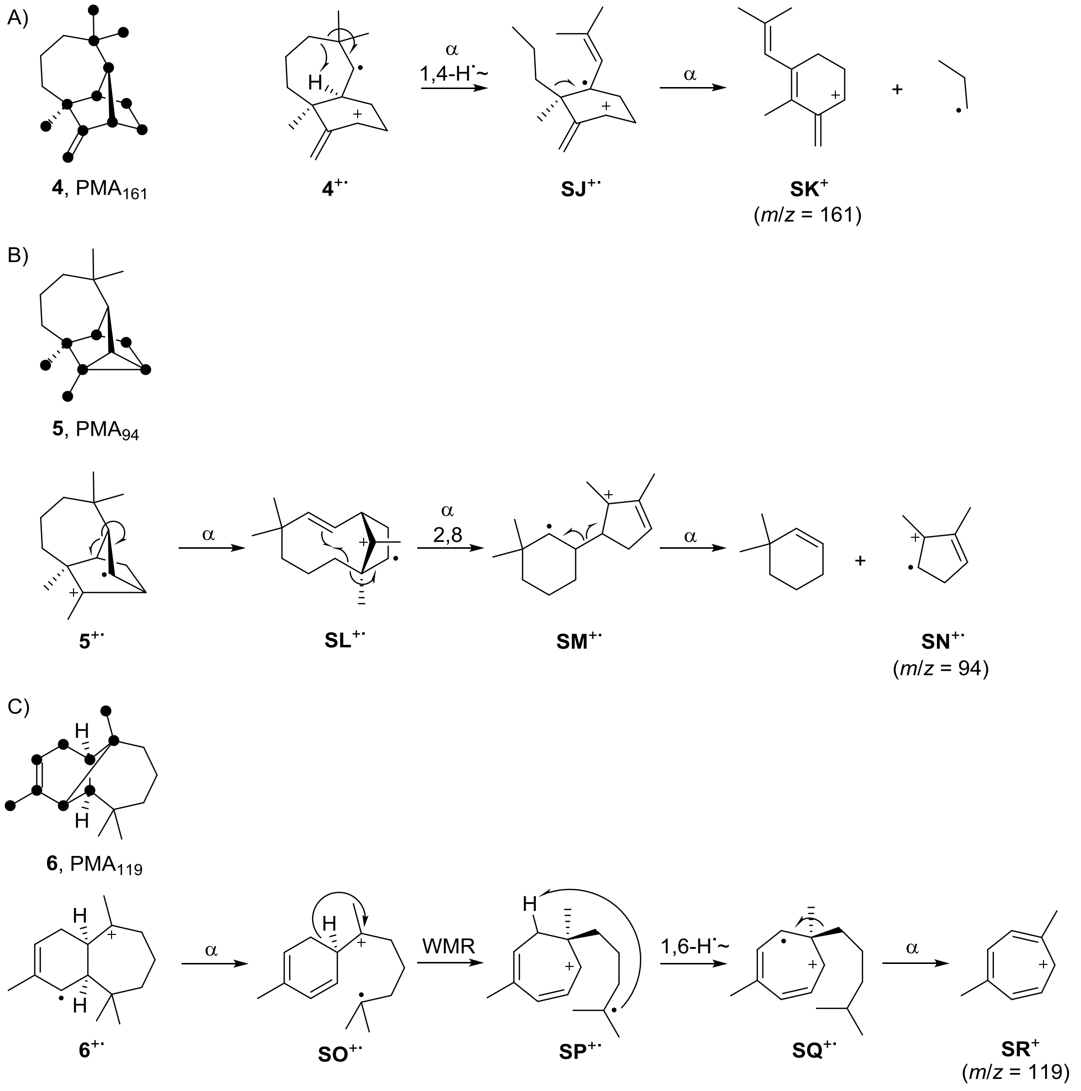

Scheme S2: Proposed EIMS fragmentation mechanisms for the fragments A) *m*/*z* = 161 of **4**, B) *m*/*z* = 94 of **5** and C) *m*/*z* = 119 of **6**. α: α-cleavage, $H^{\bullet}$~: hydrogen rearrangement. Black dots represent carbon atoms that are part of the fragment (Figures S22 – S24).

PMA_{161} of the tricyclic system **4** does not contain the geminal methyl groups in contrast to fragments with the same mass of **1** and **8** (Schemes S1 and S3). In fact, the methylene groups C-8, C-9 and C-10 are extruded. To achieve this, an ionisation of the C-1,C-2 bond to **4**$^{+\bullet}$ can be assumed, which induces cleavage next to the geminal methyl groups together with hydrogen rearrangement to yield **SJ**$^{+\bullet}$. This radical cation can easily lose a propyl radical to give the conjugated trienyl cation **SK**$^{+}$ with *m*/*z* = 161.
The intriguing fragment PMA_{94} of the tetracyclic sesquiterpene **5** requires a formal cleavage of four bonds. If ionisation at the three-membered ring to **5**$^{+\bullet}$ is assumed, an opening event to the bicyclic radical cation **SL**$^{+\bullet}$ may follow. This induces α-cleavage and ring closure to the six-membered ring in **SM**$^{+\bullet}$, which easily breaks up to 3,3-dimethylcyclohex-1-ene as a neutral loss and conjugated radical cation **SN**$^{+\bullet}$ with *m*/*z* = 94.
PMA_{119} of **6** is similar to the same fragment of **1**, but may arise through a different mechanism, which first starts with ionisation of the four-membered ring to **6**$^{+\bullet}$. A cleavage to **SO**$^{+\bullet}$ can induce ring expansion to the seven-membered ring in **SP**$^{+\bullet}$ followed by hydrogen rearrangement yielding **SQ**$^{+\bullet}$, which loses a 2-methylpentyl radical building up tropylium cation **SR**$^{+}$ with *m*/*z* = 119.

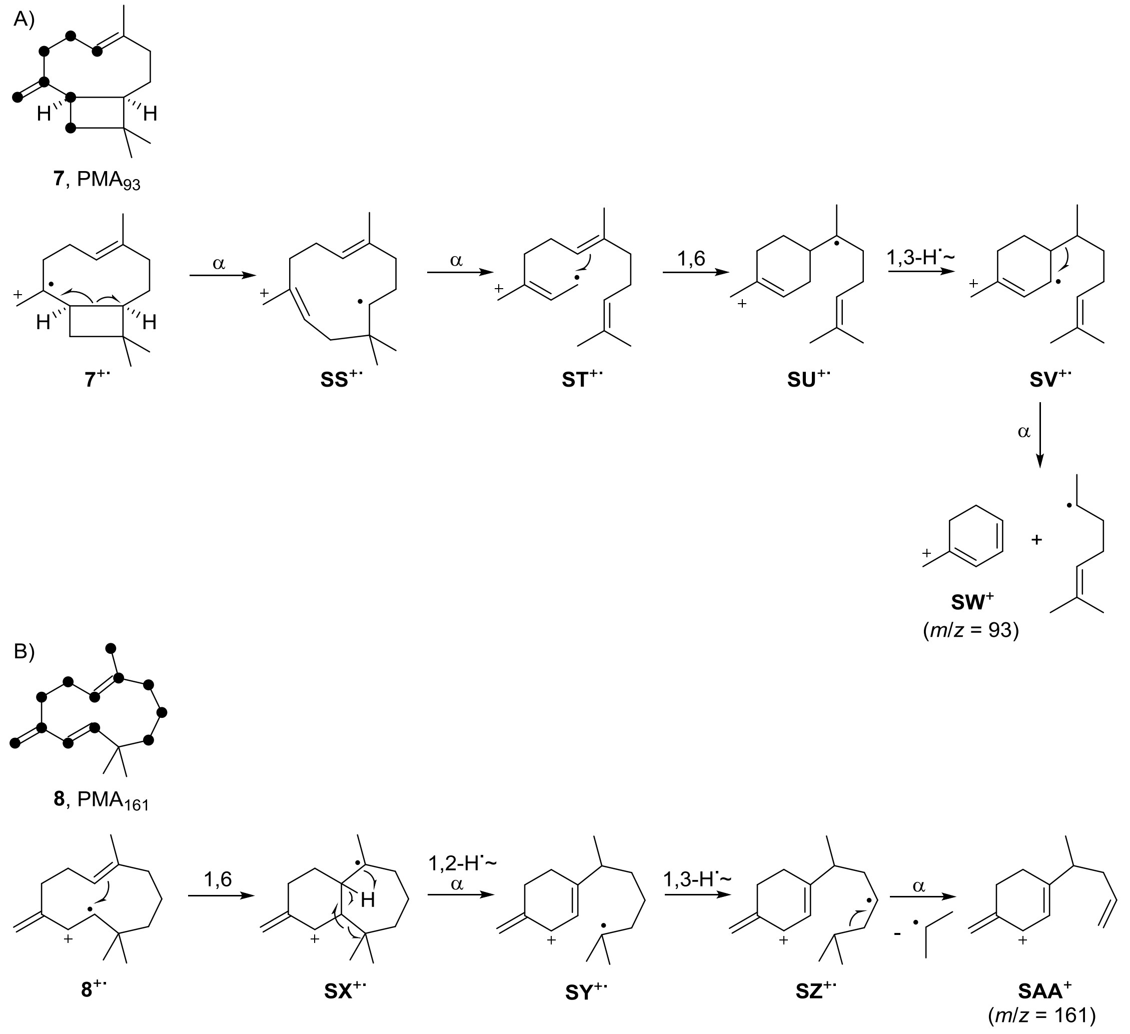

Scheme S3: Proposed EIMS fragmentation mechanisms for the fragments A) *m*/*z* = 93 of **7** and B) *m*/*z* = 161 of **8**. α: α-cleavage, $H^{\bullet}$~: hydrogen rearrangement. Black dots represent carbon atoms that are part of the fragment (Figures S25 – S26).

PMA_{93} of **7** requires three bond breaking events, including one double bond. Starting with its ionisation to $\mathbf{7}^{+\bullet}$, an α-cleavage of the four-membered ring can give access to $\mathbf{SS}^{+\bullet}$, followed by breaking the C1,C11-bond to the allylic radical cation $\mathbf{ST}^{+\bullet}$. A 1,6-ring closure furnishes $\mathbf{SU}^{+\bullet}$. Hydrogen rearrangement leads to $\mathbf{SV}^{+\bullet}$, which cleaves off a 2-methylhept-2-enyl radical yielding the conjugated cation $\mathbf{SW}^{+}$ with *m*/*z* = 93.
Similar to **1**, also **8** features a fragment PMA_{161} missing C-11, C-12 and C-13. After ionisation of the conjugated double bonds to $\mathbf{8}^{+\bullet}$, a 1,6-ring closure to $\mathbf{SX}^{+\bullet}$ can occur, followed by hydrogen rearrangement and α-cleavage to $\mathbf{SY}^{+\bullet}$. Another hydrogen movement places the radical centre in the correct position to induce cleavage of a propyl radical in $\mathbf{SZ}^{+\bullet}$ and furnishes the conjugated cation $\mathbf{SAA}^{+}$ with *m*/*z* = 161.

References

1. Rinkel, J.; Dickschat, J. S. *Beilstein J. Org. Chem.,* **2019,** *15,* 789–794.
2. Meguro, A.; Tomita, T.; Nishiyama, M.; Kuzuyama, T. *ChemBioChem,* **2013,** *14,* 316–321.
3. Dickschat, J. S.; Pahirulzaman, K. A. K.; Rabe P.; Klapschinski, T. A. *ChemBioChem,* **2014,** *15,* 810–814.
4. Bradford, M. M. *Anal. Biochem.,* **1976,** *72,* 248–254.
5. Fulmer, G. R.; Miller, A. J. M.; Sherden, N. H.; Gottlieb, H. E.; Nudelman, A.; Stoltz, B. M.; Bercaw, J. E.; Goldberg, K. I. *Organometallics,* **2010,** *29,* 2176–2179.
6. Ho, T.-L.; Chein, R.-J. *Helv. Chim. Acta,* **2006,** *89,* 231–239.
7. Quijano, C. E.; Salamanca, G.; Pino, J. A. *Flavour Fragr. J.,* **2007,** *22,* 401–406.
8. Andriamaharavo, N. R., Retention Data. *NIST Mass Spectrometry Data Center,* **2014**.
9. Lauterbach, L.; Rinkel, J.; Dickschat, J. S. *Angew. Chem. Int. Ed.,* **2018,** *57,* 8280–8283.
10. Rabe, P.; Rinkel, J.; Dolja, E.; Schmitz, T.; Nubbemeyer, B., Luu, T. H.; Dickschat, J. S. *Angew. Chem. Int. Ed.,* **2017,** *56,* 2776–2779.
11. Rabe, P.; Rinkel, J.; Nubbemeyer, B.; Köllner, T. G.; Chen, F.; Dickschat, J. S. *Angew. Chem. Int. Ed.,* **2016,** *55,* 15420–15423.
12. Bian, G.; Rinkel, J.; Wang, Z.; Lauterbach, L; Hou, A.; Yuan, Y.; Deng, Z.; Liu, T.; Dickschat, J. S. *Angew. Chem. Int. Ed.,* **2018,** *57,* 15887–15890.
13. Mitsuhashi, T.; Rinkel, J.; Okada, M.; Abe, I.; Dickschat, J. S. *Chem. Eur. J.,* **2017,** *23,* 10053–10057.
14. Rabe, P.; Barra, L.; Rinkel, J.; Riclea, R.; Citron, C. A.; Klapschinski, T. A.; Janusko, A.; Dickschat, J. S. *Angew. Chem. Int. Ed.,* **2015,** *54,* 13448–13451.
15. Raphael, R. A; Taylor, E. C.; Wynberg, H.; Eds.; Alkenylmagnesium halides. In *Advances in Organic Chemistry Methods Results*; Interscience Publishers: New York, 1960; Vol. 2, pp 1–65.
16. Schwabe, R.; Farkas, I.; Pfander, H. *Helv. Chim. Acta,* **1988,** *71,* 292–297.
17. Rinkel, J.; Lauterbach, L.; Rabe, P.; Dickschat, J. S. *Angew. Chem. Int. Ed.,* **2018,** *57,* 3238–3241.
18. Hinkley, S. F. R.; Perry, N. B.; Weavers, R. T. *Phytochemistry,* **1994,** *35,* 1489–1494.
19. Davisson, V. J.; Woodside, A. B.; Neal, T. R.; Stremler, K. E.; Muehlbacher, M; Poulter, C. D. *J. Org. Chem.,* **1986,** *51,* 4768–4779.
20. Rabe, P.; Klapschinski, T. A.; Dickschat, J. S. *ChemBioChem,* **2016,** *17,* 1333–1337.
